# Climbing the Oxidase Phase Ladder by Using Dioxygen as the Sole Oxidant: The Case Study of Costunolide

**DOI:** 10.1021/acs.orglett.4c00406

**Published:** 2024-03-29

**Authors:** Kyriaki Gennaiou, Antonis Kelesidis, Alexandros L. Zografos

**Affiliations:** Department of Chemistry, Aristotle University of Thessaloniki, Main University Campus, Thessaloniki, 54124, Greece

## Abstract

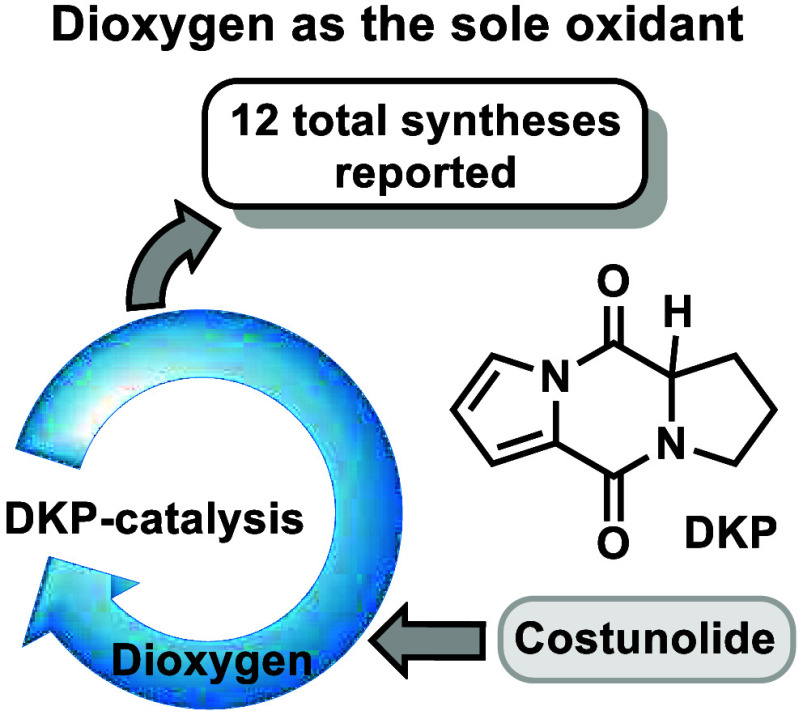

Natural sesquiterpenoid lactones are prominent scaffolds in drug discovery. Despite the progress made in their synthesis, their extensive oxidative decoration makes their chemo- and stereoselective syntheses highly challenging. Herein, we report our effort to mimic part of the oxidase phase used in the costunolide pathway to achieve the protecting-group-free total synthesis of santamarine, dehydrocostus lactone, estafiatin, and nine more related natural sesquiterpenoid lactones by using dioxygen as the sole oxidant.

Thousands of terpenoids are constantly prepared by Nature and evolve to achieve better biological responses under ever-changing life conditions. The two-phase biosynthetic logic lies in the heart of Nature’s evolution especially in terpenoid biosynthesis.^1^ Despite Nature’s apparent simplicity that builds on “common” macrocycles to access the rich carbocyclic diversity of this class of natural products (cyclase phase),^2^ it is the powerful, yet synthetically underdeveloped, machinery of monooxygenases that determines their natural complexity (oxidase phase) (Scheme 1).^3^ From the synthetic standpoint, great progress has been made over the years to replicate Nature’s efficiency in the cyclase phase.^4^ Conversely, mimicking the oxidase phase has proven to be considerably more challenging, with only a limited array of methods yielding suboptimal results in laboratory settings.^5^ Lately, the progress made in developing C–H activation processes has provided powerful alternatives that allow shorter syntheses by minimizing the functional group interconversion steps.^6^ A closer examination of Nature’s oxidative processes reveals the diverse array of monooxygenase cofactors as determinants to achieve a range of oxidation potentials, enabling the epoxidation and allylic oxidation of alkenes, but also the most challenging C–H oxidation of alkanes (Scheme 1).^7^

**Scheme 1:**
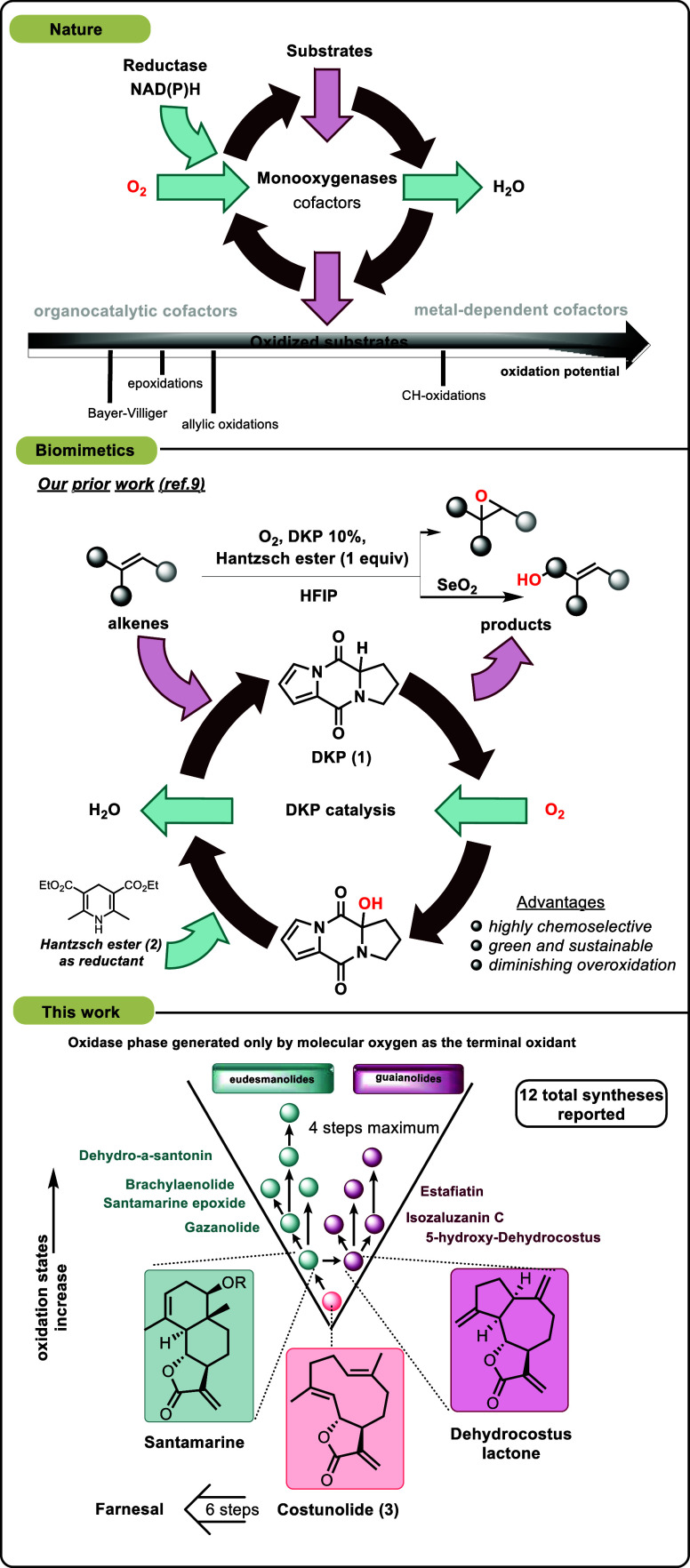
Natural, Biomimetic Processes to Access the Oxidase Phase of Sesquiterpenoids and Current Work

To address the limitations stemming from the common laboratory oxidants, including issues related to selectivity, toxicity, and low yields, researchers have devised organocatalysts and metal catalysts that emulate the monooxygenase cofactors in their capacity to activate dioxygen. However, it is worth noting that their utilization in complex settings is still rather scarce.^8^ As a result, our capacity to climb the oxidative phase ladder within a given biosynthetic pathway, relying solely on dioxygen as the primary oxidizing agent, remains uncertain. Recently, our group reported the use of a pyrrole-proline diketopiperazine (DKP) (**1**) as an efficient catalyst to activate dioxygen, which allowed the aerobic oxidation of heteroatoms,^9a^ the epoxidation and the allylic oxidation of alkenes,^9b^ and the oxidative coupling of phenols (Scheme 1).^9c^ The success of the method lay on the capacity of DKP (**1**) to form the corresponding peroxy-DKP in the presence of dioxygen, which acts as the primary oxidant, while the use of Hantzsch ester (**2**) allows for the regeneration of the catalyst effectively mimicking the function of a reductase (Scheme 1).^9^

Willing to test the efficiency of our method in a more complex setting, we considered the oxidase phase of sesquiterpenoid costunolide (**3**), a well-established precursor to 6,12-sesquiterpenoid lactones, as a case study (Scheme 1).^10^ Our objective was to mimic the performance of monooxygenases and enable the total synthesis of several natural 6,12-sesquiterpenoid lactones using dioxygen as the sole oxidant. We report here the results of our endeavor.

Costunolide was readily prepared following a modification of Corey’s cyclase protocol^11^ to obtain a gram scale quantity in just seven steps from farnesol (**4**) with an overall yield of 7% (Scheme 2). The requisite bromide **7** for Corey’s cyclization was prepared from **6** by the sequential aerobic oxidation of farnesol (**4**) to farnesal (**5**) using Ma’s procedure,^12^ and then to the corresponding allylic alcohol **6** using DKP in the presence of Hantzsch ester and SeO_2_ (Scheme 2).^9^

**Scheme 2:**
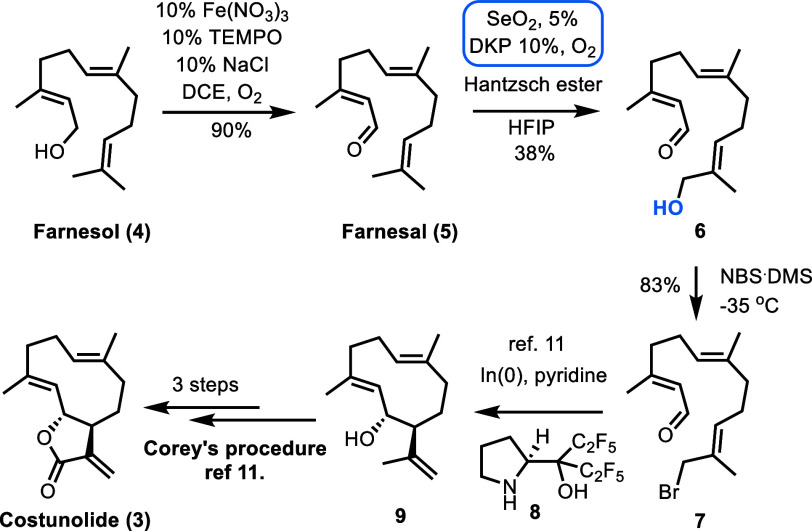
Total Synthesis of Costunolide (**3**) Based on Modified Corey’s Cyclase Phase of Farnesol

When costunolide (**3**) was subjected to our original DKP-mediated organocatalytic conditions, using 10 mol % of DKP and 1 equiv of Hantzsch ester in HFIP, under dioxygen, we were able to isolate both reynosin (**11**)^13^ and santamarine (**12**)^14^ in 75% combined yield in a **12**:**11** = 5:1 ratio (Scheme 3). The latter was attributed to a chemo- and stereoselective aerobic epoxidation of the C1 and C10 alkenes of costunolide to **10**, followed by its spontaneous cyclization to form **11** and **12**. To our delight, the use of a 3:1 mixture of DCM and HFIP to run the reaction afforded costunolide epoxide (**10**)^15^ and the first total synthesis of 1β-hydroxyarbusculin A (**13**),^16^ in 47% and 9% yield, respectively.^17^ The isolation of hydroxy-arbusculin A (**13**) suggests either a peroxy radical tandem addition and cyclization process or, to some extent, a radical cleavage of the epoxide. To confirm this hypothesis, costunolide epoxide (**10**) was allowed to stir in HFIP. This produced the same 5:1 mixture of santamarine (**12**) and reynosin (**11**) but this time without any trace of 1β-hydroxyarbusculin A (**13**). In sharp contrast, when costunolide epoxide (**10**) was stirred in the presence of Hantzsch ester and dioxygen in acetone under blue light irradiation, a radical pathway was initiated producing 1β-hydroxy-arbusculin (**13**) in 44% yield along with a 1:1 mixture of santamarine (**12**) and reynosin (**11**) (55% combined yield). The latter is believed to be formed through radical cleavage of the epoxide followed by intramolecular cyclization quenched by dioxygen (Scheme 3). Further transformation of costunolide (**3**) involved the first total synthesis of melambolide aristolochin (**14**) by *E*- to *Z*-isomerization of the C1–C10 alkene.^18^ The latter was obtained by applying aerobic DKP-catalysis conditions with a catalytic amount of SeO_2_ (Scheme 3). This result was particularly surprising and counterintuitive as no hydroxylation product was observed in the process compared to the SeO_2_/*t*-BuOOH conditions previously reported in the literature.^19^ This outcome ultimately excludes the classic ene-[2,3]-sigmatropic rearrangement sequence and supports instead the potential intermediacy of a peroxometal pathway.^9,20^

**Scheme 3:**
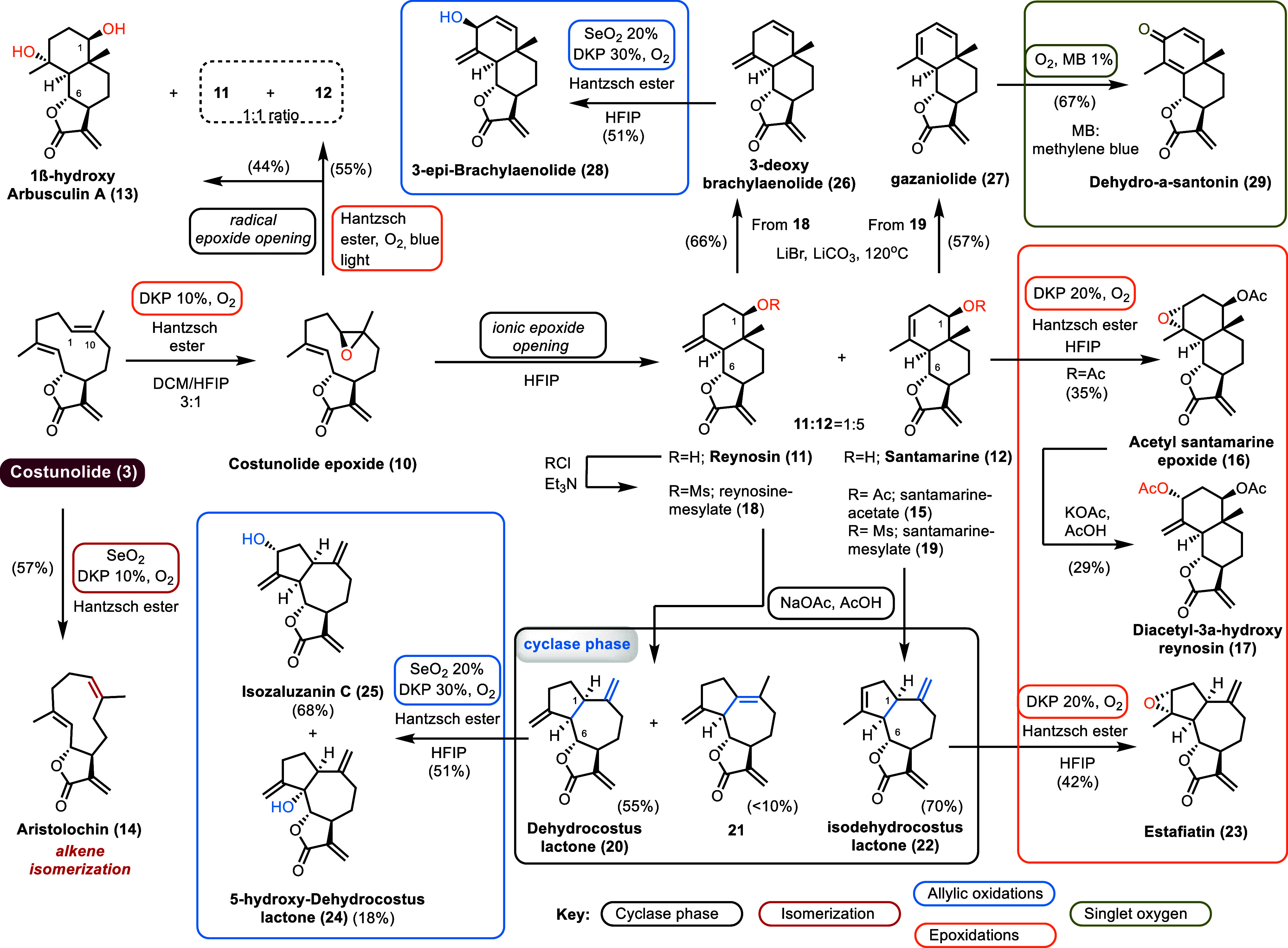
DKP-Based Aerobic Oxidation for Costunolide (**3**) and Generation of Further Complexity by the Cyclase Phase of Reynosin (**11**) and Santamarine (**12**)

The inability of our aerobic process to perform an epoxidation was first observed when the DKP aerobic oxidation of santamarine (**12**) was investigated. This was not surprising considering the lack of reactivity we had previously observed with allylic and homoallylic alcohols.^9b^ The same lack of reactivity was also observed when subjecting reynosin (**11**) to our aerobic allylic oxidation conditions using DKP, SeO_2_, and Hantzsch ester.^9b^ Nonetheless, the epoxidation of santamarine acetate (**15**),^14c^ readily prepared by acetyl chloride on **12**, can be obtained if an excess of DKP catalyst (20 mol %) and Hantzsch ester (2 equiv) is added. This led to the stereoselective synthesis of acetyl-santamarine epoxide (**16**),^21,14a^ albeit in moderate yields (35%) (Scheme 3; orange box). To further enrich its decoration, the treatment of epoxide **16** with potassium acetate in acetic acid resulted in the synthesis of diacetyl hydroxy santamarine **17** (Scheme 3; orange box).^22^

With ample quantities of santamarine (**12**) and reynosin (**11**) at our disposal, we considered a cyclase phase to gain access to the biologically intriguing 6,12-*Asteraceae* lactone guaiane cores (Scheme 3; black box). Hence, activation of the secondary alcohol at the C1 position of both reynosin and santamarine by mesylation to obtain derivatives **18** and **19** [MsCl (3 equiv), Et_3_N (6 equiv), THF], followed by solvolytic rearrangement with potassium acetate in acetic acid, resulted in the synthesis of dehydrocostus lactone (**20**) (55% yield from reynosin) and isodehydrococtus lactone (**22**) (70% yield from santamarine) as the major products, along with the isomerized alkene product **21** in less than 10% yield for the reaction of reynosin (Scheme 3; black box).^23^

The epoxidation of isodehydrocostus lactone (**22**) using higher loadings of DKP (20–25 mol %) demonstrated remarkable chemoselectivity and stereoselectivity leading to the exclusive epoxidation of the more substituted alkene leading to the total synthesis of yet another natural product, namely, estafiatin (**23**) (Scheme 3; orange box).^24^ Furthermore, allylic oxidation of dehydrocostus lactone (**20**) using DKP, SeO_2_, dioxygen, and Hantzsch ester provided a mixture of 5-hydroxy-dehydrocostus lactone (**24**) and isozaluzanin C (**25**) in 18% and 68% yields respectively (Scheme 3; blue box).^25^

Finally, reynosin and santamarine mesylates **18** and **19** were also used to prepare 3-deoxy-brachylaenolide (**26**) (66% yield from **18**) and gazanolide (**27**) (57% yield from **19**) with the aid of lithium bromide and lithium carbonate at 120 °C according to previously known procedures (Scheme 3).^26^

In contrast to the nonselective hydroxylation process of dehydrocostus lactone, allylic oxidation of 3-deoxy-brachylaenolide (**26**) returned stereoselectively 3-epi-brachylaenolide (**28**)^27^ in 51% yield (Scheme 3; blue box).

Combining DKP aerobic oxidations with the established synthetic utility of singlet oxygen chemistry^28^ further enriches its synthetic potential. To highlight this goal, gazaniolide (**27**) delivered by the DKP process described above was treated with dioxygen and methylene blue under regular light to cleanly provide dehydro-α-santonin (**29**)^29^ in 67% yield (Scheme 3; green box).

In conclusion, the current manuscript pinpoints the ability of DKP-based aerobic oxidations to mimic monooxygenase behavior in the oxidase phase of costunolide to selectively deliver 12 total syntheses of 6,12-sesquiterpenoid lactones. The utilized method due to its simplicity, low cost, and toxicity enables libraries of natural products from appropriate common carbocyclic scaffolds to be easily delivered.

## Data Availability

The data underlying this study are available in the published article and its Supporting Information.

## Supporting Information Available

Synthetic protocols and spectroscopic characterization; ^1^H NMR and ^13^C NMR spectra of new compounds; comparison of spectral data with isolated natural compounds (PDF)
