# Supplementary material for: Climbing the Oxidase Phase Ladder by Using Dioxygen as the Sole Oxidant: The Case Study of Costunolide
Source: Org Lett. 2024 Mar 29;26(15):2934–8. doi: 10.1021/acs.orglett.4c00406 (PMC11187638; doi:10.1021/acs.orglett.4c00406)
Supplement: Supplementary file 1 — ol4c00406_si_001.pdf [file ol4c00406_si_001.pdf]

Supporting Information

Climbing the Oxidase Phase Ladder by Using Dioxygen as the Sole Oxidant: The Case Study of Costunolide.

Kyriaki Gennaiou, Antonis Kelesidis, Alexandros L. Zografos*

Department of Chemistry, Aristotle University of Thessaloniki, Main University Campus, Thessaloniki, 54124, Greece

Email: alzograf@chem.auth.gr

Table of contents

	Page
1. Materials and Methods	S3
2. The pyrrole proline diketopiperazine (DKP) catalytic system	S4
3. Modified total synthesis of costunolide	S5
4. Aerobic oxidation of costunolide	S7
5. Cyclase Phase of Santamarine and Reynosin - 6,12-*Asteraceae* lactone guaiane cores	S11
6. Further oxidative decoration	S15
7. Copies of NMR spectra	S20
8. References	S41

1. Materials and Methods

The reaction for the preparation of 2,5-diketopiperazine (DKP) was conducted in a Schlenk tube under positive argon pressure. The solvent and the materials were carefully degassed with the use of dry ice under freeze-pump-thaw technique. DKP was kept in sealed tubes under positive pressure of dry argon upon preparation and was used in caution of atmospheric air when tubes were opened. In general, DKP can be weighted without the use of a glovebox, but it needs to be protected from air as soon as it comes in contact with polar solvents or base. Hantzsch ester is also labile to air and light, so it should be stored in dark colored sealed tubes.

Anhydrous solvents were either obtained from commercial sources (dry DMF) or dried accordingly. Dry methylene chloride (CH_2Cl_2) was obtained by refluxing the solvent with CaH_2. All solvents were kept under Ar using molecular sieves 4Å in their bottles. Petroleum ether refers to the 40-60°C boiling fraction. Commercially available reagents were purchased at the highest commercial quality and used without further purification or where specified, purified by standard techniques.

Reactions were monitored by thin-layer chromatography (TLC) carried out on S-2 0.25 mm E. Merck silica gel plates (60F-254) using UV light as visualizing agent (λ_{max}= 254 nm or 360 nm) and ethanolic *p-anisaldehyde* or *Seebach* as developing solutions, followed by heating. E. Merck silica gel (60, particle size 0.040–0.063 mm) was used for flash column chromatography.

NMR spectra were recorded at 298 K using an Agilent Technologies DD2 500 MHz or a Bruker Avance 300 MHz spectrometer and calibrated by residual solvent peaks. ^{1}H NMR spectra were recorded at 500 MHz and 300 MHz and residual solvent peaks were used as an internal reference ($CDCl_3$ δ 7.26). Data are reported as follows: chemical shift in ppm, multiplicity (s = singlet, brs = broad singlet, d = doublet, t = triplet, q = quartet, m = multiplet or overlap of nonequivalent resonances, coupling constants are reported in Hz and integration is included. ^{13}C NMR spectra were recorded at 125 MHz or 75MHz and residual solvent peaks were used as an internal reference ($CDCl_3$ δ 77.00). Data are reported as follows: chemical shift in ppm, multiplicity deduced.

Optical rotations were recorded on a Krüss Optronic polarimeter at 589 nm and are reported in units of 10–1(deg cm2 g–1). High-resolution mass spectra (HRMS) were recorded on an Agilent ESI-TOF (time of light) mass spectrometer at a 4000V emitter voltage.

Spectroscopic data of synthesized natural products are compared with the isolated ones reported in the literature or previously synthesized ones (where isolation spectroscopic data is incomplete). In these cases, the numbering of the carbon atoms in the skeletons follows the logic presented below:

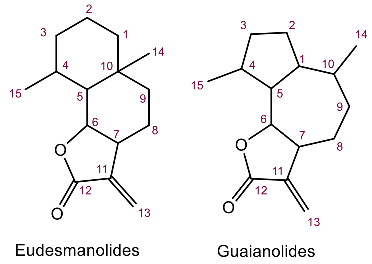

2. The pyrrole-proline diketopiperazine (DKP) catalytic system

A quick look into our aerobic catalytic system

Lately, our group reported the development of pyrrole-proline diketopiperazine (DKP) (**1**) as an efficient catalyst to activate dioxygen, allowing the aerobic oxidation of heteroatoms,[9a] the epoxidation and the allylic oxidation of alkenes[9b] and the oxidative coupling of phenols.[9c] Its success is relying upon the direct formation of peroxy-DKP by mixing DKP with dioxygen that serves as the active oxidant of the process, while utilization of Hantzsch ester (**2**) ensures the regeneration of the catalyst adopting a role of a reductase mimic.

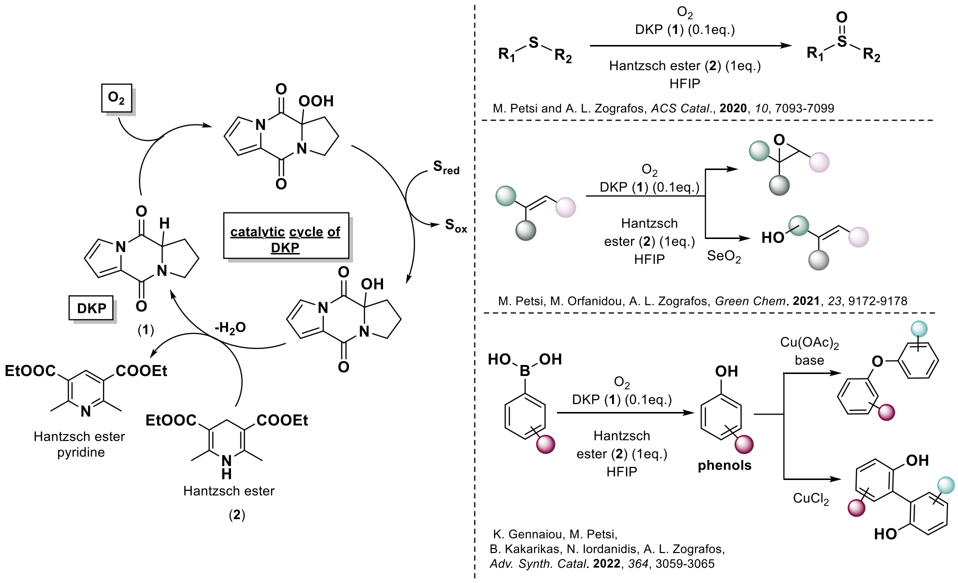

In this work, we envisioned harnessing the DKP catalytic system to mimic monooxygenase performance in order to achieve the total synthesis of several 6,12-sesquiterpenoid lactones using dioxygen as the sole oxidant.

2.1 Preparation of the DKP catalyst

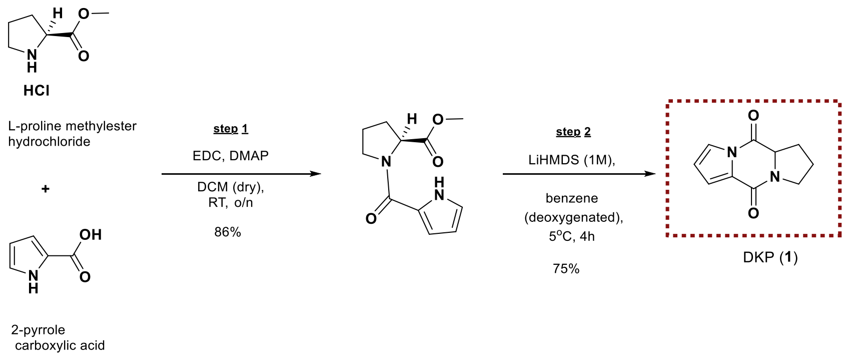

Step 1

To a mixture of 2-pyrrolecarboxylic acid (311.1 mg, 2.80 mmol, 1.0 eq.) and *L*-proline methyl ester (602.9 mg, 3.64 mmol, 1.3 equiv.) in anhydrous DCM (12 mL), 1-(3-dimethylaminopropyl)-3 ethylcarbodiimide (697.8 mg, 3.64 mmol, 1.3 eq.) and DMAP (11.2 mg, 0.092 mmol, 0.033 eq.) were added under argon at 0°C. After 30 min of stirring at 0°C and 12h at room temperature, the reaction mixture was quenched with water and was extracted with DCM (2 x 10 mL). The combined organic layers washed with $NaHCO_3$ (2 x 10 mL) and then dried with $MgSO_4$. After removal of the solvent under reduced pressure and subsequent flash column chromatography of the obtained residue on silica-gel (eluent: hexane:EtOAc 1:1, TLC: *Rf* = 0.3 upon hexane:EtOAc 1:1, UV active on TLC, stains greenish upon *p-anisaldehyde* staining) gave the coupling product in 86% (535.1 mg) yield as a white foam; Recorded NMR was found identical to the reported values.[30] ^{1}H NMR (500 MHz, $CDCl_3$): δ_H 9.67 (brs, 1H), 6.95 (m, 1H), 6.66 (m, 1H), 6.27 (m, 1H), 4.68 (s, 1H), 4.01 – 3.93 (m, 1H), 3.88 – 3.84 (m, 1H), 3.74 (s, 3H),2.27 – 2.18 (m, 2H), 2.10 – 2.00 (m, 2H) ppm; ^{13}C NMR (125 MHz, $CDCl_3$): δ_C 172.9, 160.4, 125.3, 121.4, 112.6, 110.1, 60.0, 52.2, 48.2, 28.7, 25.3 ppm.

Step 2

Methyl ester (200 mg, 0.90 mmol, 1.0 eq.) was washed with benzene twice, and then dissolved in benzene (6 mL) in a Schlenk tube under argon, which was connected to a vacuum pump and immersed in a dry ice bath. The substrate was deoxygenated 3 times and then lithium bis(trimethylsilyl)amide 1M (1.1 ml, 1.08 mmol, 1.2 eq.) was added and the mixture was stirred for 4h at 5°C. The mixture was quenched with CH_3COOH/CH_3COONa buffer solution (pH 3.7, 10 mL) and the products were extracted with EtOAc (5 x 10). After removal of the solvents under reduced pressure, the desirable DKP (1) product was received as a white solid in 75% (128.3 mg) yield and was used without further purification. TLC: *Rf* = 0.35 upon DCM:EtOAc (1:2), UV active on TLC, stains blue upon *Seebach* staining. Recorded NMR was found identical to the reported values.[9] ^1H NMR (500 MHz, $CDCl_3$): δ_H 7.46 (dd, *J* = 3.3, 1.8 Hz,1H), 7.07 (dd, *J* = 3.3, 1.8 Hz,1H), 6.48 (dd, *J* = 3.3, 3.3 Hz, 1H), 4.49 (dd, *J* = 9.5, 6.2 Hz, 1H), 3.88 – 3.80 (m, 1H), 3.70 – 3.61 (m, 1H), 2.60 – 2.54 (m, 1H), 2.19 – 2.13 (m, 1H), 2.10 – 1.96 (m, 2H) ppm; ^{13}C NMR (125 MHz, $CDCl_3$): δ_C 164.5, 155.2, 127.4, 118.8, 117.9, 115.6, 61.5, 44.8, 29.0, 22.3 ppm.

2.2 Preparation of Hantzsch ester[31] (2)

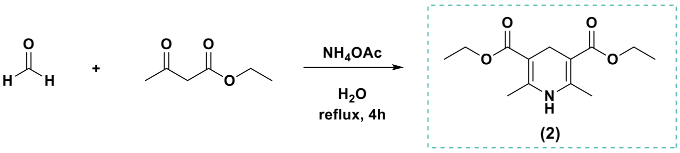

According to literature,[31] formaldehyde solution (1.4 ml, 0.05 mol, 1.0 eq.), ethylacetoacetate (10.1 ml, 0.08 mol, 1.5 eq.) and ammonium acetate (3.08 g, 0.04 mmol, 0.8 eq.) were dissolved in H_2O (40 ml) in a round bottom flask and stirred at 80°C (using an oil bath) for 4 hours. After this period of time, yellow balls were formed and the reaction mixture was cooled to room temperature. The resulting solid was filtered, washed with icy water (30 ml) and cold acetone (30 ml) to afford diethyl 2,6-dimethyl-1,4-dihydropyridine-3,5-dicarboxylate or Hantzsch ester (2) as a yellow fine solid in 83% (10.5 g) yield. NMR spectra were fully consistent with reported literature values.[31] ^1H NMR (500 MHz, $CDCl_3$): δ_H 5.17 (brs, 1H), 4.16 (q, *J* = 7.1 Hz, 4H), 3.26 (s, 2H), 2.19 (s, 6H), 1.28 (t, *J* = 7.1 Hz, 6H) ppm; ^{13}C NMR (125 MHz, $CDCl_3$): δ_C 168.0, 144.7, 99.5, 59.6, 24.8, 19.2, 14.5 ppm.

3 Modified total synthesis of costunolide following Corey's cyclase protocol[11]

Costunolide was prepared following a modification of Corey's cyclase protocol[11] and used as starting material to mimic its oxidase phase by using molecular oxygen as the sole oxidant. Alternatively, costunolide can be isolated directly by its extraction and chromatographic purification from *Saussurea lappa*.

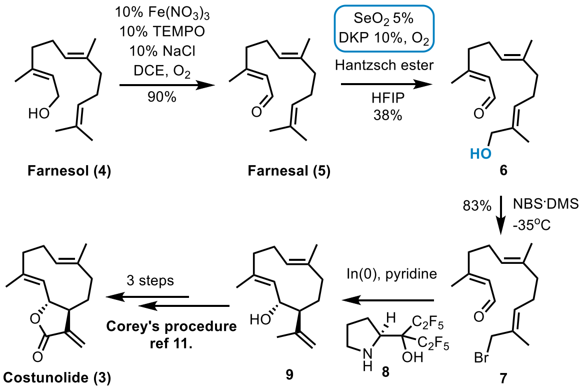

Preparation of farnesal (5) (step 1).

Following Ma's typical procedure:[12] To a 25 mL flask were added $Fe(NO_3)_3{\cdot}9H_2O$ (404 mg, 1 mmol, 0.1 eq.), TEMPO (156.2 mg, 1 mmol, 0.1 eq.), NaCl (58.4 mg, 1 mmol 0.1 eq.), and DCE (100 mL). Farnesol (2.5 ml, 10.0 mmol, 1 eq.) was then added to the suspension and the resulting mixture was placed in oxygen atmosphere with the use of a balloon and was stirred at room temperature for 12h. After evaporation, the crude product was purified by column chromatography using silica gel (eluent: PS/Et_2O 48:1, TLC: *Rf* = 0.48 upon PS /Et_2O 5:1, , UV active on TLC, stains purple upon *p*-anisaldehyde staining) to afford farnesal (**5**) in 90% (1.98 g) yield as a colorless oil: ^1H NMR (500 MHz, $CDCl_3$)): δ_H 10.01 (d, *J* = 8.1 Hz, 1H), 5.90 (d, *J* = 8.0 Hz, 1H), 5.14 – 5.06 (m, 2H), 2.29 – 2.17 (m, 8H), 2.11 – 1.99 (m, 3H), 1.70 (s, 3H), 1.62 (s, 6H) ppm; ^{13}C NMR (125 MHz, $CDCl_3$): δ_C 191.3, 163.9, 136.5, 131.5, 127.4, 124.1, 122.4, 40.6, 39.6, 26.6, 25.9, 25.6, 17.7, 17.6, 16.0 ppm.

Preparation of hydroxyfarnesal 6 (step 2).

Following our allylic oxidation protocol:[9] Farnesal (**5**, 28.6 mg, 0.13 mmol, 1 eq.) was dissolved in the corresponding solvent (2 ml) in a 4 ml screw capped vial with a stirring bar. Then, Hantzsch ester (**2**, 32.9 mg, 0.13 mmol, 1 eq.), DKP (**1**, 2.5 mg, 0.013 mmol, 0.1 eq.) and SeO_2 (0.7 mg, 0.0064 mmol, 0.05 eq.) were added in one portion and the vial was tightly capped with a rubber septum. An exit needle and a pipette were introduced in the septum, through which dioxygen was bubbled continuously by a balloon. Care was taken to adjust slow bubbling in the reaction vial to avoid solvent's evaporation. The reaction was stirred at room temperature overnight under dioxygen atmosphere. Upon completion of time, the reaction mixture was diluted with DCM and extracted consecutively with water and brine. The combined organic layers were dried over $MgSO_4$, filtrated, and evaporated under reduced pressure. The residue was purified by flash column chromatography on silica gel (eluent: PS/EtOAc 4:1, TLC: R_f = 0.14 upon PS/EtOAc 10:1, UV active on TLC, stains purple upon *p-anisaldehyde* staining) to give **6** as a colorless oil in 38% (11.7 mg) yield. ^{1}H NMR (500 MHz, $CDCl_3$): δ_H 9.97 (d, *J* = 8.1 Hz, 1H), 5.87 (d, *J* = 8.1 Hz, 1H), 5.35 (t, *J* = 6.9 Hz, 1H), 5.08 (d, *J* = 5.6 Hz, 1H), 3.98 (s, 2H), 2.25 – 2.20 (m, 5H), 2.13 – 2.10 (m, 1H), 2.08 – 2.05 (m, 1H), 2.04 – 1.99 (m, 3H), 1.65 (s, 3H), 1.60 (s, 3H) ppm.

Preparation of bromofarnesal 7 (step 3).

NBS (1.22 g, 6.85 mmol, 1.2 eq.) was dissolved in DCM (10 mL) followed by dimethyl sulfide (0.53 g, 8.56 mmol, 1.5 eq.) under argon at -35 °C. The obtained solution was maintained at this temperature for 1 h before being warmed up to 0 °C for an additional 30min. The NBS-adduct was re-cooled to -35 °C prior to the dropwise introduction of compound **6** (1.43 g, 5.62 mmol, 1 eq.) in DCM (5 mL), followed by slowly warming up the mixture to room temperature within an hour. The reaction was worked up by pouring the reaction mixture into an aqueous solution of saturated $NaHCO_3$. The mixture was extracted with ethyl acetate twice and the combined organic layers were dried over $MgSO_4$ and concentrated under vacuo to yield compound **7** as oil in 83% (1.39 g) yield, which was used in the next step without further purification. ^{1}H NMR (500 MHz, $CDCl_3$): δ_H 10.01 (d, *J* = 8.1 Hz, 1H), 5.89 (d, *J* = 8.1 Hz, 1H), 5.56 (t, *J* = 7.0 Hz, 1H), 5.10 (t, *J* = 6.2 Hz, 1H), 3.98 (s, 2H), 2.26 (ddt, *J* = 15.4, 11.8, 5.0 Hz, 4H), 2.19 (s, 3H), 2.13 (q, *J* = 7.1 Hz, 2H), 2.04 (t, *J* = 7.4 Hz, 2H), 1.76 (s, 3H), 1.62 (s, 3H) ppm.

Preparation of (+)-Costunolide (3) (steps 4-7).

Costunolide (**3**) was obtained following identical conditions as those described by Corey for the subsequent 4 steps.[11] It was delivered in 25% yield over the 4 steps, as a pale yellow solid. TLC: R_f = 0.75 (hexane/EtOAc 2:1, UV inactive on TLC, stains purple upon *p-anisaldehyde* staining). Column chromatography eluent: hexane/EtOAc 17:1. Spectroscopic and physical data were in accordance with literature reported values.[32] $[a]_D^{20}$ = +119 (c = 0.3, $CHCl_3$); mp 109 – 111°C; ^{1}H NMR (500 MHz, $CDCl_3$): δ_H 6.23 (d, *J* = 3.6 Hz, 1H), 5.5 (d, *J* = 3.2 Hz, 1H), 4.82 (dd, *J* = 11.7, 4.3 Hz, 1H), 4.71 (d, *J* = 9.9 Hz, 1H), 4.54 (t, *J* = 9.6 Hz, 1H), 2.54 (dd, *J* = 10.3, 6.9 Hz, 1H), 2.42 (dd, *J* = 13.3, 6.0 Hz, 1H), 2.31 – 2.26 (m, 1H), 2.23 (dd, *J* = 12.0, 4.7 Hz, 1H), 2.18 – 2.13 (m, 1H), 2.09 (dd, *J* = 6.1, 4.7 Hz, 1H), 2.06 (dd, *J* = 6.0, 1.5 Hz, 1H), 2.0 (td, *J* = 11.4, 5.1 Hz, 1H), 1.67 (d, *J* = 1.2 Hz, 3H), 1.70 – 1.61 (m, 1H), 1.39 (s, 3H) ppm; ^{13}C NMR (125 MHz, $CDCl_3$): δ_C 170.5, 141.5, 140.0, 136.9, 127.2, 127.0, 119.7, 81.9, 50.4, 40.9, 39.4, 28.0, 26.2, 17.3, 16.1 ppm.

Direct comparison of proton and carbon shift values of synthesized costunolide with the reported isolation.[32]

Position	Proton Shifts synthesized	Proton Shifts Isolation	Carbon Shifts synthesized	Carbon Shifts Isolation
**1**	4.82	4.81	127.0	127.0
**2**			28.0	28.0
**3**			40.9	40.9
**4**			140.0	140.0
**5**	4.71	4.71	127.2	127.2
**6**	4.54	4.54	81.9	81.9
**7**			50.4	50.3
**8**			26.2	26.2
**9**			39.4	39.4
**10**			136.9	136.9
**11**			141.5	141.5

**12**			170.5	170.5
**13**	6.23 (a); 5.50 (b)	6.24 (a); 5.50 (b)	119.7	119.4
**14**	1.39	1.39	16.1	16.0
**15**	1.67	1.67	17.3	17.2

4. Aerobic oxidation of costunolide

*Syntheses of Santamarine (**12**), Reynosin (**11**), Costunolide epoxide (**10**) and 1β-hydroxy- Arbusculin A (**13**)*

Willing to evaluate the efficiency of our method in the decoration of sesquiterpenoids' skeleton, we initiated our survey by applying the original conditions of DKP organocatalysis to costunolide. That is, using one equivalent of Hanztsch ester and 10% of DKP-H in HFIP at room temperature, under an oxygen balloon induced atmosphere, overnight. This attempt afforded Santamarine (**12**) and Reynosin (**11**) cleanly in 75% combined yield in a **12**:**11**=5:1 ratio.

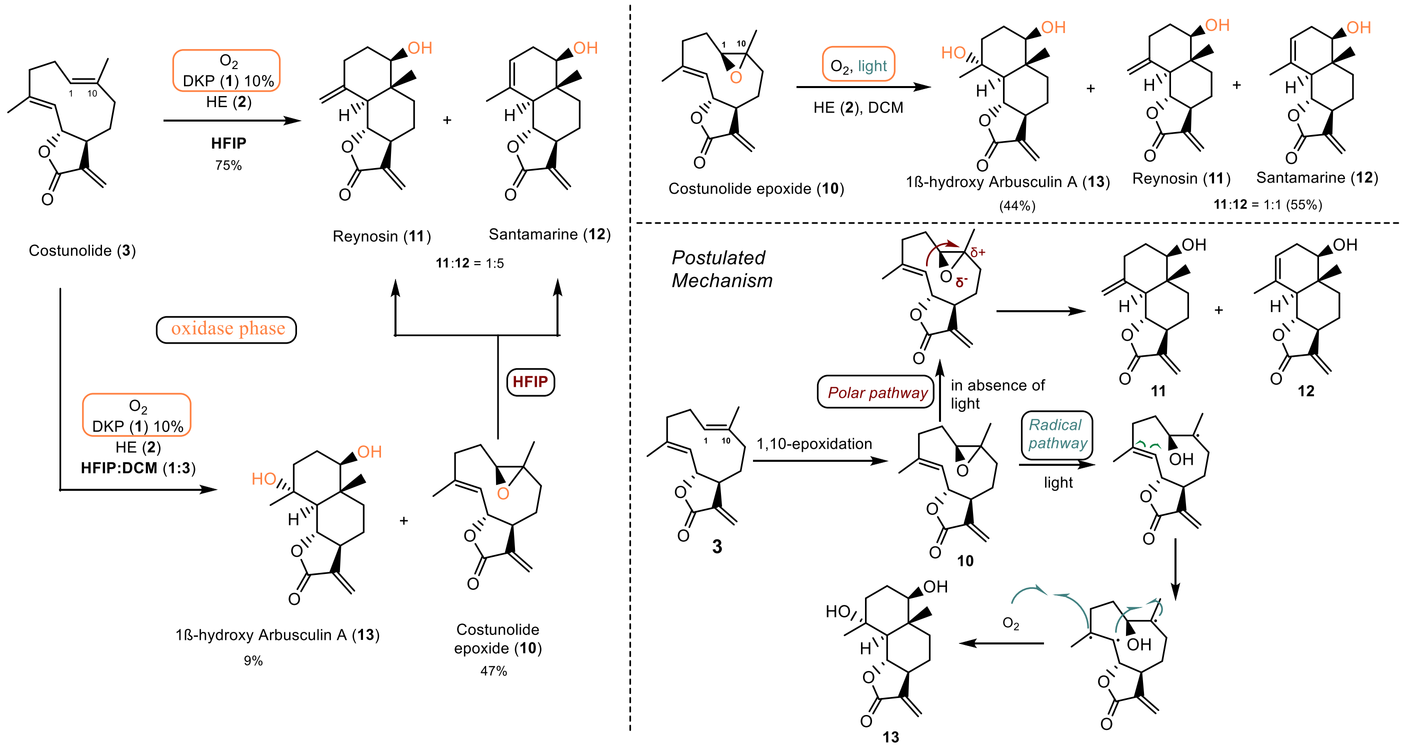

It was postulated that this process involves the chemo- and stereoselective aerobic epoxidation of costunolide (**3**) at C 1-C10 alkene, followed by its spontaneous cyclization to **11** and **12**. To corroborate our hypothesis, we slightly changed the reaction conditions, using a mixture of DCM/HFIP (3:1) as the reaction solvent. This would limit the epoxide opening, induced by the HFIP. Indeed, the rationale was reinforced by isolating the respective costunolide epoxide (**10**) and the first total synthesis of 1β-hydroxy-Arbusculin A (**13**), in 47% and 9% yield respectively. Removing HFIP from the reaction altogether and using DCM as the reaction's sole solvent, failed to deliver any products except for unreacted starting material. The same outcome was observed when DMF was used as the reaction solvent.

Isolation of hydroxy-β-arbusculin A (**13**) supports, at least partially, a radical cleavage of the epoxide, in contrast to what was initially considered to be a purely HFIP promoted epoxide opening. To verify this assumption, costunolide epoxide (**10**) was allowed to stir in HFIP so as to produce Santamarine (**12**) and Reynosin (**11**) in the same ratio (**12**:**11**=5:1), without a trace of hydroxy-β-Arbusculin A (**13**). In sharp contrast, when costunolide epoxide (**10**) was stirred in the presence of Hantzsch ester and dioxygen in DCM under light, a radical pathway was initiated producing hydroxy-β-Arbusculin (**13**) in 44% yield, along with a 1:1 mixture of Santamarine (**12**) and Reynosin (**11**) (55% combined yield). The latter is postulated to involve the radical cleavage of the epoxide followed by intramolecular cyclization, quenched by dioxygen.

Experimental procedure for costunolide epoxidation. Preparation of Santamarine (12), Reynosin (11), Costunolide epoxide (10) and 1β-hydroxy-Arbusculin A (13).

Isolated products when HFIP was used as the reaction solvent.

Costunolide (**3**, 40 mg, 0.1721 mmol, 1 eq.) was dissolved in HFIP (2 ml) in a 4 ml screw capped vial with a stirring bar. Then, Hantzsch ester (**2**, 43.6 mg, 0.1721 mmol, 1 eq.) and DKP (**1**, 3.2 mg, 0.0172 mmol, 0.1 eq.) were added in one portion and the vial was tightly capped using a rubber septum. An exit needle and a pipette were introduced in the septum, through which dioxygen was bubbled continuously by a balloon. Care was taken to adjust slow bubbling in the reaction vial to avoid solvent's evaporation. The reaction was stirred at room temperature, under dioxygen atmosphere, overnight. After the completion of time, the reaction mixture was diluted with DCM and extracted consecutively with water and brine. The combined organic layers were dried over $MgSO_4$, filtrated and evaporated under reduced pressure. The residue was purified by flash column chromatography on silica gel to give Santamarine (**12**, eluent: PS/acetone 10:1, TLC: R_f = 0.48 upon PS/acetone 2:1, UV active on TLC, stains black upon *p-anisaldehyde* staining, physical state: white solid) and Reynosin (**11,** eluent: PS/acetone 10:1, TLC: R_f = 0.44 upon PS/acetone 2:1, UV active on TLC, stains black upon *p-anisaldehyde* staining, physical state: colorless crystals)in 63% (26.9 mg) and 12% (5.1 mg) yields respectively. Spectroscopic and physical data were in accordance with literature reported values.[15b] For **Santamarine** (**12**): $[a]_D^{20}$ = +99.2 (c = 0.5, $CHCl_3$); mp 135 – 137 °C; ^{1}H NMR (500 MHz, $CDCl_3$): δ_H 6.07 (d, *J* = 3.2 Hz, 1H), 5.41 (d, *J* = 3.0 Hz, 1H), 5.34 (brs, 1H), 3.94 (t, *J* = 10.9 Hz, 1H), 3.67 (dd, *J* = 9.9, 6.7 Hz, 1H), 2.49 (td, *J* = 11.6, 3.1 Hz, 1H), 2.37 (dd, *J* = 28.8, 14.2 Hz, 2H), 2.11 – 2.02 (m, 2H), 2.01 – 1.93 (m, 1H), 1.65 (ddd, *J* = 26.2, 13.4, 4.0 Hz, 1H), 1.84 (s, 3H), 1.29 (ddd, *J* = 17.0, 13.0, 4.9 Hz, 2H), 0.88 (s, 3H) ppm; ^{13}C NMR (125 MHz, $CDCl_3$): δ_C 170.8, 138.9, 133.5, 121.2, 116.8, 81.5, 75.2, 51.1, 51.0, 40.9, 34.2, 32.8, 23.3, 21.2, 11.0 ppm. For **Reynosin** (**11**): $[a]_D^{20}$ = +177.2 (c = 0.1, EtOH); mp 144 – 146 °C; ^{1}H NMR (500 MHz, $CDCl_3$): δ_H 6.08 (d, *J* = 3.0 Hz, 1H), 5.41 (d, *J* = 2.9 Hz, 1H), 4.98 (brs, 1H), 4.86 (brs, 1H), 4.03 (t, *J* = 10.9 Hz, 1H), 3.52 (dd, *J* = 11.5, 4.5 Hz, 1H), 2.54 (td, *J* = 11.4, 3.0 Hz, 1H), 2.33 (dd, *J* = 13.4, 3.3 Hz, 1H), 2.18 (d, *J* = 11.0 Hz, 1H), 2.16 – 2.11 (m, 1H), 2.11 – 2.05 (m, 2H), 1.81 – 1.87 (m, 1H), 1.64 – 1.52 (m, 2H), 1.39 – 1.32 (m, 1H), 0.81 (s, 3H) ppm; ^{13}C NMR (125 MHz, $CDCl_3$): δ_C 170.6, 142.4, 139.2, 117.1, 110.6, 79.5, 78.2, 52.9, 49.6, 42.9, 35.7, 33.5, 31.3, 21.4, 11.6 ppm.

Isolated products when a mixture of DCM: HFIP (3:1) was used as the reaction solvent.

Costunolide (**3**, 40 mg, 0.1721 mmol, 1 eq.) was dissolved in HFIP:DCM (1:3, 2 ml total) in a 4 ml screw capped vial with a stirring bar. Then, Hantzsch ester (**2**, 43.6 mg, 0.1721 mmol, 1 eq.) and DKP (**1**, 3.2 mg, 0.0172 mmol, 0.1 eq.) were added in one portion and the vial was tightly capped by a rubber septum. An exit needle and a pipette were introduced in the septum, through which dioxygen was bubbled continuously by a balloon. Care was taken to adjust slow bubbling in the reaction vial to avoid solvent's evaporation. The reaction was stirred at room temperature, under dioxygen atmosphere, overnight. After the completion of time, the reaction mixture was diluted with DCM and extracted consecutively with water and brine. The combined organic layers were dried over $MgSO_4$, filtrated and evaporated under reduced pressure. The residue was purified by flash column chromatography on silica gel to give Costunolide epoxide (**10**, eluent: hexane/EtOAc 3:1, TLC: R_f = 0.32 upon PS/EtOAc 2:1, UV active on TLC, stains brown-red upon *p-anisaldehyde* staining, physical state: white crystal solid) in 47% (20 mg) yield, and 1ß-hydroxy Arbusculin A (**13**, eluent: PS/acetone 2:1, TLC: R_f = 0.33 upon PS/acetone 2:1, UV active on TLC, stains blue-black upon *p-anisaldehyde*, physical state: colorless oil) in 9% (4.1 mg) yield. Spectroscopic and physical data were in accordance with literature reported values.[15b, 32] For **Costunolide epoxide** (**10**): $[a]_D^{20}$ = +55 (c = 0.1, MeOH); mp 126 – 128 °C; ^{1}H NMR (500 MHz, $CDCl_3$): δ_H 6.27 (d, *J* = 3.4 Hz, 1H), 5.51 (d, *J* = 3.0 Hz, 1H), 5.30 (d, *J* = 9.9 Hz, 1H), 4.61 (t, *J* = 9.6 Hz, 1H), 2.72 (d, *J* = 11.2 Hz, 1H), 2.58 (t, *J* = 9.3 Hz, 1H), 2.41 (ddd, *J* = 21.2, 14.0, 6.2 Hz, 2H), 2.27 (d, *J* = 12.3 Hz, 1H), 2.22 – 2.10 (m, 2H), 1.84 (s, 3H), 1.54 (dd, *J* = 18.9, 8.1 Hz, 1H), 1.44 (ddd, *J* = 25.0, 13.5, 4.6 Hz, 1H), 1.19 (d, *J* = 13.6 Hz, 1H),1.14 (s, 3H) ppm; ^{13}C NMR (125 MHz, $CDCl_3$): δ_C 170.1, 144.4, 139.6, 123.9, 119.6, 80.8, 67.7, 61.3, 51.0, 39.3, 36.2, 25.3, 24.9, 17.7, 17.1 ppm. For **1ß-hydroxy Arbusculin A** (**13**): $[a]_D^{20}$ = -9.5 (c = 0.1, MeOH); ^{1}H NMR (500 MHz, $CDCl_3$): δ_H 6.03 (d, *J* = 3.2 Hz, 1H), 5.37 (d, *J* = 3.1 Hz, 1H), 4.03 (t, *J* = 11.1 Hz, 1H), 3.39 – 3.34 (m, 1H), 2.55– 2.47 (m, 1H), 2.00 (ddd, *J* = 13.2, 6.7, 3.8 Hz, 1H), 1.96 – 1.91 (m, 1H), 1.76 (d, *J* = 11.5 Hz, 1H), 1.72 (dd, *J* = 9.1, 2.7 Hz, 1H), 1.68 – 1.64 (m, 1H), 1.55 (d, *J* = 8.4 Hz, 1H), 1.53 – 1.49 (m, 1H), 1.48 – 1.45 (m, 1H), 1.27 (s, 3H), 1.26 – 1.22 (m, 1H), 0.89 (s, 3H) ppm; ^{13}C NMR (125 MHz, $CDCl_3$): δ_C 169.6, 138.0, 118.1, 80.9, 78.4, 71.2, 56.5, 50.4, 40.9, 38.9, 38.1, 28.3, 24.3, 21.8, 13.6 ppm.

Direct comparison of proton and carbon shift values of synthesized santamarine with the reported isolation.[33]

Position	Proton Shifts synthesized	Proton Shifts Isolation	Carbon Shifts synthesized41.9	Carbon Shifts Isolation
**1**	3.67	3.68	75.2	75.3
**2**	2.37 (a); 1.97 (b)	2.40 (a); 1.97 (b)	32.8	32.8
**3**	5.34	5.35	121.2	121.3
**4**	-	-	133.5	133.4
**5**	2.37	2.35	51.1	51.1
**6**	3.94	3.95	81.5	81.5
**7**	2.49	2.50	51.0	51.0
**8**	2.08 (a); 1.65 (b)	2.10 (a); 1.67 (b)	21.2	21.2
**9**	1.29 (a); 2.08 (b);	1.31 (a); 2.06 (b)	34.2	34.2
**10**	-	-	40.9	40.9
**11**	-	-	138.9	138.9
**12**	-	-	170.8	171.0
**13**	6.07 (a); 5.41 (a)	6.08 (a); 5.41 (b)	116.8	116.8
**14**	0.88	0.88	11.0	11.0
**15**	1.84	1.84	23.3	23.3

Direct comparison of proton and carbon shift values of synthesized reynosin with the reported isolation.[33]

Position	Proton Shifts synthesized	Proton Shifts Isolation	Carbon Shifts synthesized	Carbon Shifts Isolation
**1**	3.52	3.53	78.2	78.1
**2**	1.84 (a); 1.58 (b)	1.85 (a); 1.57 (b)	31.3	31.3
**3**	2.13 (a); 2.33 (b)	2.14 (a); 2.34 (b)	35.7	35.7
**4**	-	-	142.4	142.4
**5**	2.18	2.19	52.3	53.0
**6**	4.03	4.03	79.5	79.6
**7**	2.54	2.54	49.6	49.6
**8**	2.08 (a); 1.58 (b)	2.09 (a); 1.60 (b)	21.4	21.5
**9**	1.36 (a); 2.08 (b)	1.36 (a); 2.10 (b)	33.5	33.6
**10**	-	-	42.9	43.0
**11**	-	-	139.2	139.1
**12**	-	-	170.6	170.5
**13**	5.41 (a); 6.08 (b)	5.42 (a); 6.09 (b)	117.1	117.0
**14**	0.81	0.82	110.6	110.5
**15**	4.86 (a); 4.98 (b)	4.87 (a); 4.99 (b)	11.6	11.7

Direct comparison of proton and carbon shift values of synthesized costunolide epoxide with previously reported synthesis.[15b]

Position	Proton Shifts synthesized	Proton Shifts of previously synthesized product	Carbon Shifts synthesized	Carbon Shifts of previously synthesized product
**1**	2.72	2.71	67.7	67.8
**2**	2.10 – 2.22 (a); 1.44 (b)	2.12 (a); 1.43 (b)	25.3	25.5
**3**	2.41 (a); 2.27 (b)	2.41 (a); 2.26 (b)	39.3	39.5
**4**	-	-	139.6	139.7
**5**	5.30	5.29	123.9	124.1
**6**	4.61	4.61	80.8	81.0
**7**	2.58	2.56	51.0	51.2
**8**	1.54 (a); 2.10 – 2.22 (b)	1.53 (a); 2.18 (b)	24.9	25.0
**9**	2.41 (a); 1.19 (b)	2.38 (a); 1.17 (b)	36.2	36.3

**10**	-	-	61.3	61.5
**11**	-	-	144.4	144.6
**12**	-	-	170.1	170.3
**13**	5.51 (a); 6.27 (b)	5.51 (a); 6.27 (b)	119.6	119.8
**14**	1.14	1.13	17.7	17.8
**15**	1.84	1.84	17.1	17.2

Direct comparison of proton and carbon shift values of synthesized 1β-hydroxy-arbusculin with the reported isolation.[32]

Position	Proton Shifts synthesized	Proton Shifts Isolation	Carbon Shifts synthesized	Carbon Shifts Isolation
**1**	3.37	3.34	78.4	77.9
**2**	1.45 – 1.68	1.47 – 1.73	28.3	27.9
**3**	1.45 – 1.68	1.47 – 1.73	38.1	38.0
**4**	-	-	71.2	71.4
**5**	1.76	1.76	56.5	56.3
**6**	4.03	4.05	80.9	81.0
**7**	2.50	2.52	50.4	50.3
**8**	2.00 (a); 1.47 (b)	2.00 (a); 1.42 (b)	21.8	21.6
**9**	1.24 (a); 1.93 (b)	1.21 (a); 1.94 (b)	38.8	38.8
**10**	-	-	40.9	41.8
**11**	-	-	138.0	137.9
**12**	-	-	169.6	169.9
**13**	5.37 (a); 6.03(b)	5.40 (a); 6.04 (b)	118.1	118.1
**14**	0.89	0.89	13.6	13.5
**15**	1.27	1.28	24.3	24.1

*Light induced synthesis of 1ß-hydroxy Arbusculin A (**13**) from Costunolide epoxide (**10**).*

Costunolide epoxide (**10**, 5.5 mg, 0.0221 mmol, 1 eq.) was dissolved in acetone (2 ml) in a 4 ml screw capped vial with a stirring bar. Then, Hantzsch ester (**2**, 5.6 mg, 0.0221 mmol, 1 eq.) was added and the vial was tightly capped by a rubber septum. An exit needle and a pipette were introduced in the septum, through which dioxygen was bubbled continuously by a balloon. Care was taken to adjust slow bubbling in the reaction vial to avoid solvent's evaporation. The reaction was stirred at room temperature, under dioxygen atmosphere and blue light irradiation overnight. After the completion of time, the reaction mixture was diluted with DCM and extracted consecutively with water and brine. The combined organic layers were dried over $MgSO_4$, filtrated, and evaporated under reduced pressure. The residue was purified by flash column chromatography on silica gel as mentioned above, to give **13** in 44% (2.6 mg), along with a 1:1 mixture of **12** and **11** (55% combined yield, 1.5 mg each).

Allylic oxidation conditions for costunolide

Costunolide, on oxidation with SeO_2, is known to give melampolides skeletons, such as *Soulangianolide A* and others. In this case, the proposed mechanism involves a bulky peroxometal intermediate *t*-BuOO–SeO_3H. The change in the stereochemistry of the double bond, from *E* to *Z*, takes place through a rotation before the sigmatropic rearrangement occurs.

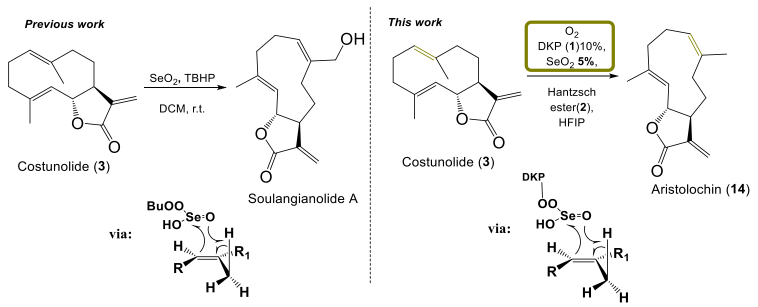

Determined that utilization of our aerobic protocol for allylic oxidations would produce the same results as above, we subjected costunolide to aerobic DKP-catalysis in the presence of catalytic SeO_2. To our surprise, the latter was isomerized to melambolide Aristolochin (**14**) and no hydroxylation occurred, in sharp contrast to previous references utilizing the SeO_2/t-BuOOH protocol.[19] Although the delivered product supports the intermediacy of a DKP-peroxometal pathway, the reaction mechanism has not yet been clarified. To rule out the chance of isomerization due to HFIP's acidity, we dissolved Costunolide (**3**) to HFIP and left it stir to room temperature overnight. No reaction was observed. Raising the amount of the SeO_2 used, from catalytic to stoichiometric, in the absence of DKP and dioxygen only returned a complex mixture of labile products, which were not further characterized.

Experimental procedure for costunolide allylic oxidation, in presence of catalytic SeO_2. Preparation of Aristolochin (14)

Costunolide (**3**, 30 mg, 0.1291 mmol, 1 eq.) was dissolved in the corresponding solvent (2 ml) in a 4 ml screw capped vial with a stirring bar. Then, Hantzsch ester (**2**, 32.7 mg, 0.1291 mmol, 1 eq.), DKP (**1**, 2.4 mg, 0.0129 mmol, 0.1 eq.) and SeO_2 (0.7 mg, 0.0064 mmol, 0.05 eq.) were added in one portion and the vial was tightly capped by a rubber septum. An exit needle and a pipette were introduced in the septum, through which dioxygen was bubbled continuously by a balloon. Care was taken to adjust slow bubbling in the reaction vial to avoid solvent's evaporation. The reaction was stirred at room temperature, under dioxygen atmosphere, overnight. After the completion of time, the reaction mixture was diluted with DCM and extracted consecutively with water and brine. The combined organic layers were dried over $MgSO_4$, filtrated, and evaporated under reduced pressure. The residue was purified by flash column chromatography on silica gel (eluent: PS/acetone 26:1, TLC: R_f = 0.67 upon PS/acetone 2:1, UV inactive on TLC, stains blue-black upon *seebach* staining) to give **14** as colorless plates in 57% (17.1 mg) yield. Spectroscopic and physical data were in accordance with literature reported values.[18] ($[\alpha]_D^{20}$ = +2.6 (c = 0.5, $CHCl_3$); mp 144 – 146 °C; HRMS (ESI): m/z: $[M+H]^+$ calculated for $C_{15}H_{20}O_2H^+$ 233.1536; found: 233.1538; ^1H NMR (500 MHz, $CDCl_3$): δ_H 6.15 (d, J = 3.4 Hz, 1H), 5.41 (d, J = 3.1 Hz, 1H), 5.16 (t, J = 8.3 Hz, 1H), 5.04 (d, J = 10.2 Hz, 1H), 4.56 (t, J = 9.8 Hz, 1H), 2.48 (ddd, J = 12.4, 8.1, 3.5 Hz, 1H), 2.29 – 2.21 (m, 1H), 2.16 – 2.11 (m, 2H), 2.02 (td, J = 13.6, 5.1 Hz, 2H), 1.97 – 1.82 (m, 2H), 1.81 (s, 3H), 1.67 (s, 3H), 1.54 – 1.46 (m, 1H) ppm; ^{13}C NMR (75 MHz, $CDCl_3$): δ_C 170.6, 140.3, 138.3, 136.9, 125.0, 124.6, 118.5, 80.7, 44.9, 38.9, 26.7, 25.4, 25.1, 21.9, 17.2 ppm.

Direct comparison of proton and carbon shift values of synthesized aristolochin with the reported isolation.[18]

Position	Proton Shifts synthesized	Proton Shifts Isolation	Carbon Shifts synthesized	Carbon Shifts Isolation
**1**	5.16	5.16	124.6	124.9
**2**	1.82 – 2.06	1.76 – 2.1	26.7	26.7
**3**	1.82 – 2.06 (a); 2.11 – 2.16 (b)	1.76 – 2.1; 2.14 (b)	38.9	38.9
**4**	-	-	138.3	138.1
**5**	5.04	5.05	124.6	124.4
**6**	4.56	4.57	80.7	80.7
**7**	2.48	2.48	44.9	44.9
**8**	1.46 – 1.54 (a); 2.21 – 2.27 (b)	1.50 (a); 2.27 (b)	25.4	25.4
**9**	1.82-2.06	1.76-2.10	25.1	25.1

**10**	-	-	136.9	136.7
**11**	-	-	140.3	140.1
**12**	-	-	170.6	170.3
**13**	5.41 (a); 6.15 (b)	5.41 (a); 6.14 (b)	118.5	118.4
**14**	1.67	1.68	21.9	21.9
**15**	1.81	1.81	17.2	17.2

5. Cyclase Phase of Santamarine and Reynosin - 6,12-*Asteraceae* lactone guaiane cores

Although detailed biosynthetic blueprints are missing for most guaianolide members, it is generally assumed that an oxidized germacrene-type macrocycle serves as their precursor. In our work, we sought to use Santamarine (**12**) and Reynosin (**11**) as parent materials to access the main core of *Asteraceae* family. Acetylation or mesylation of the former *Eudesmanolides*, followed by solvolytic rearrangement with sodium acetate in acetic acid, resulted in the synthesis of Dehydrocostus lactone (**20**) (from Reynosin (**11**)) in 55% yield along with isomerized on the C-10 alkene congener (**21**) in less than 10% yield, and Isodehydrocostus lactone (**22**) (from Santamarine (**12**)) in 70% yield. Santamarine and Reynosin mesylates (**19** and **18)** were also used to prepare Gazanolide (**27**) (57% yield from **19**) and 3-deoxy Brachylaenolide (**26**) (66% yield from **18**) with the aid of lithium bromide and lithium carbonate at 120°C according to previously known procedures.[26]

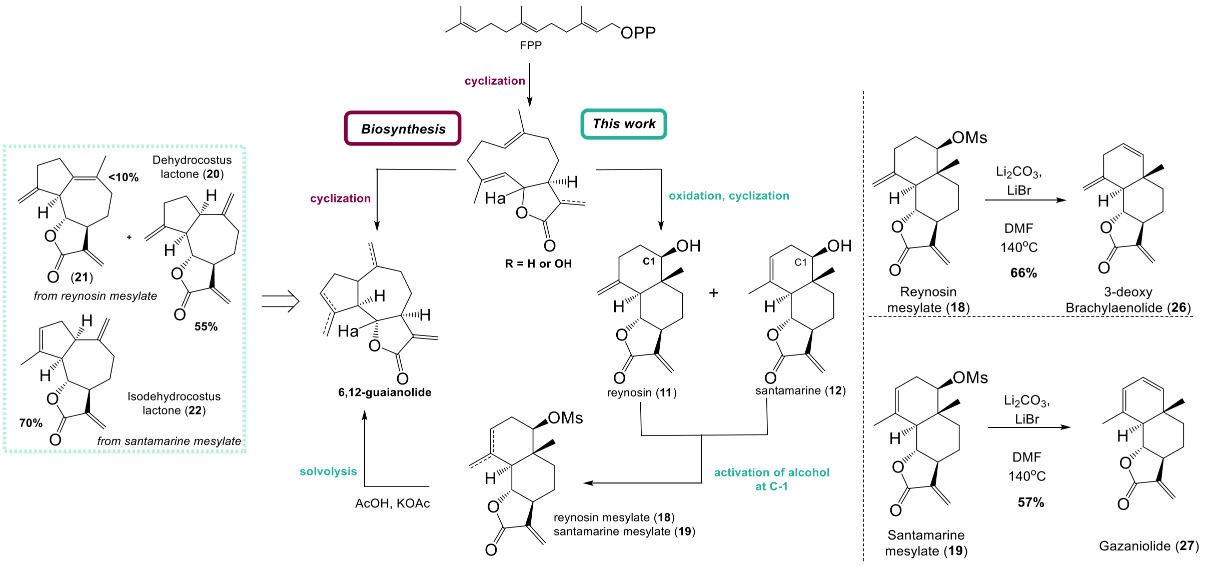

Activation of the secondary alcohol at C-1 position followed by solvolytic rearrangement.

Preparation of Dehydrocostus lactone (20)

To a flame dried round bottom flask, equipped with a magnetic stirring bar, anhydrous Et_3N (41 μl, 0.2899 mmol, 6 eq) and DMAP (3.2 mg, 0.0265 mmol, 0.55 eq.) were added consecutively, under argon. The mixture was then cooled, using an ice bath. Reynosin (**11**, 12 mg, 0.048 3mmol, 1 eq.) was dissolved in dry DCM (4 ml) and added to the mixture. Lastly, MsCl (11 μl, 0.2174 mmol, 3 eq.) was added dropwise to the reaction solution which was allowed to warm up to room temperature and was stirred for about 1 hour. Upon completion, the reaction mixture was quenched using NH_4Cl (sat.) and extracted with DCM. The combined organic layers were dried over $MgSO_4$, filtrated and evaporated under reduced pressure. The residue was purified by flash column chromatography on silica gel (eluent: PS/EtOAc 1:1, TLC: R_f = 0.48 upon PS/EtOAc 1:1, UV inactive on TLC, stains blue-black upon *p*-anisaldehyde staining) to give Reynosin mesylate **18** as a white foam in 70% (11 mg) yield and used directly in the next step. ^{1}H NMR (500 MHz, $CDCl_3$): δ_H 6.10 (d, *J* = 3.1 Hz, 1H), 5.43 (d, *J* = 2.9 Hz, 1H), 5.04 (brs, 1H), 4.92 (brs, 1H), 4.59 (dd, *J* = 11.8, 4.7 Hz, 1H), 3.99 (t, *J* = 10.8 Hz, 1H), 3.03 (s, 3H), 2.55 (td, *J* = 11.3, 3.1 Hz, 1H), 2.39 (dd, *J* = 13.9, 3.6 Hz, 1H), 2.27 (d, *J* = 10.8 Hz, 1H), 2.21 – 2.07 (m, 3H), 2.04 – 2.00 (m, 1H), 1.87 (ddd, *J* = 25.8, 12.5, 5.2 Hz, 1H), 1.58 (ddd, *J* = 15.2, 12.8, 2.9 Hz, 1H), 1.48 (td, *J* = 13.1, 3.6 Hz, 1H), 0.89 (s, 3H) ppm; ^{13}C NMR (125 MHz, $CDCl_3$): δ_C 170.3, 140.7, 138.6, 117.6, 111.9, 87.6, 78.8, 52.9, 49.3, 42.3, 39.1, 35.6, 32.9, 29.1, 21.2, 12.5 ppm.

Reynosin mesylate (**18**, 11 mg, 0.0336 mmol, 1 eq.) was dissolved in a solution of 0.5M CH_3COOH/CH_3COOK (43 mg, 13 eq. of CH_3COOK dissolved in 0.88 ml CH_3COOH), in a sealed tube equipped with a magnetic stirring bar. The reaction mixture was then immersed in an oil bath and heated up to 120°C, where it was allowed to stir overnight. Upon completion, the reaction mixture was quenched with brine and extracted with EA. The combined organic layers were dried over $MgSO_4$, filtrated and evaporated under reduced pressure. The residue was purified by flash column chromatography on silica gel (eluent: PS/Et_2O 8:1, TLC: R_f = 0.59 upon PS/Et_2O 2:1, UV inactive on TLC, stains blue upon *p*-anisaldehyde staining) to give Dehydrocostus lactone (**20)** as a white powder in 55% (4.2 mg). Spectroscopic and physical data were in accordance with literature reported values.[34] ($[\alpha]_D^{20}$ = −14.1 (c = 0.5, $CHCl_3$); mp 47.5 – 49 °C ; ^{1}H NMR (500 MHz, $CDCl_3$): δ_H 6.19 (d, *J* = 3.4 Hz, 1H), 5.45 (d, *J* = 3.1 Hz, 1H), 5.24 (brs, 1H), 5.04 (brs, 1H), 4.86 (brs, 1H), 4.78 (brs, 1H), 3.93 (t, *J* = 9.3 Hz, 1H), 2.92 – 2.81 (m, 3H), 2.56 – 2.43 (m, 3H), 2.21 (ddd, *J* = 13.4, 9.5, 4.3 Hz, 1H), 2.13 (ddd, *J* = 12.9, 10.2, 5.9 Hz, 1H), 1.95 – 1.89 (m, 1H), 1.88 – 1.80 (m, 1H), 1.39 (ddd, *J* = 22.9, 11.2, 5.6 Hz, 1H) ppm; ^{13}C NMR (125 MHz, $CDCl_3$): δ_C 170.2, 151.2, 149.2, 139.8, 120.1, 112.6, 109.6, 85.2, 52.0, 47.6, 45.1, 36.2, 32.6, 30.9, 30.3 ppm.

Direct comparison of proton and carbon shift values of synthesized Dehydrocostus lactone with the reported isolation.[34]

Position	Proton Shifts synthesized	Proton Shifts Isolation	Carbon Shifts synthesized	Carbon Shifts Isolation
**1**	2.86	2.85	47.6	47.5
**2**	1.92	1.92	32.6	32.6
**3**	2.49	2.46	30.3	30.3
**4**	-	-	149.2	149.2
**5**	2.86	2.85	52.0	52.0
**6**	3.93	3.93	85.2	85.2
**7**	2.86	2.85	45.1	45.0
**8**	1.39 (a); 2.21 (b)	1.46 (a); 2.27 (b)	30.9	30.9
**9**	2.49 (a); 2.21 (b)	2.46 (a); 2.27(b)	36.2	36.3
**10**	-	-	151.2	151.2
**11**	-	-	139.8	139.7
**12**	-	-	170.2	170.2
**13**	6.19 (a); 5.45 (b)	6.19 (a); 5.46 (b)	120.1	120.2
**14**	5.24 (a); 5.04 (b)	5.23 (a); 5.03 (b)	109.6	109.5
**15**	4.86 (a); 4.78 (b)	4.90 (a); 4.78 (b)	112.6	112.6

Preparation of Isodehydrocostus lactone (22)

To a flame dried round bottom flask, equipped with a magnetic stirring bar, anhydrous Et_3N (270 µl, 1.9330 mmol, 6 eq) and DMAP (21.6 mg, 0.1771 mmol, 0.55 eq.) were added consecutively, under argon. The mixture was then cooled, using an ice bath. Santamarine (**12**, 80 mg, 0.3221 mmol, 1 eq.) was dissolved in dry DCM (4 ml) and added to the mixture. Lastly, MsCl (75 µl, 0.9665 mmol, 3 eq.) was added dropwise to the reaction solution which was allowed to warm up to room temperature and stirred for about 1 hour. Upon completion, the reaction mixture was quenched with NH_4Cl (sat.) and extracted with DCM. The combined organic layers were dried over $MgSO_4$, filtrated and evaporated under reduced pressure. The residue was purified by flash column chromatography on silica gel (eluent: cyclohexane/Et_2O 1:1, TLC: R_f = 0.25 upon cyclohexane/Et_2O 1:2, UV active on TLC, stains blue upon *p*-anisaldehyde staining) to give Santamarine mesylate **19** as a white foam in 73% (76.7 mg) yield and used directly in the next step. ^{1}H NMR (500 MHz, $CDCl_3$): δ_H 6.10 (d, *J* = 3.2 Hz, 1H), 5.43 (d, *J* = 3.0 Hz, 1H), 5.35 (brs, 1H), 4.71 (dd, *J* = 9.8, 7.0 Hz, 1H), 3.92 (t, *J* = 11.0 Hz, 1H), 3.03 (s, 3H), 2.62 (dd, *J* = 17.5, 2.0 Hz, 1H), 2.52 – 2.45 (m, 2H), 2.38 – 2.30 (m, 1H), 2.10 (ddd, *J* = 13.3, 6.6, 3.6 Hz, 1H), 2.01 (dt, *J* = 13.6, 3.0 Hz, 1H), 1.85 (s, 3H), 1.64 (ddd, *J* = 25.7, 12.9, 3.7 Hz, 1H), 1.51 – 1.44 (m, 1H), 0.99 (d, *J* = 5.4 Hz, 3H) ppm; ^{13}C NMR (125 MHz, $CDCl_3$): δ_C 170.4, 138.4, 133.7, 117.3, 85.2, 80.7, 51.0, 50.7, 40.0, 38.9, 34.4, 30.7, 23.1, 20.9,12.1 ppm.

Santamarine mesylate (**19**, 50 mg, 0.1531 mmol, 1 eq.) was dissolved in a solution of 0.5M CH_3COOH/CH_3COOK (200.2 mg, 13 eq. of CH_3COOK dissolved in 4.08 ml CH_3COOH), in a sealed tube equipped with a magnetic stirring bar. The reaction mixture was then immersed in an oil bath and heated up to 120°C, where it was allowed to stir overnight. After the completion of time, the reaction mixture was quenched with brine and extracted with EA. The combined organic layers were dried over $MgSO_4$, filtrated and evaporated under reduced pressure. The residue was purified by flash column chromatography on silica gel (eluent: PS/Et_2O 20:1, TLC: R_f = 0.47 upon PS/Et_2O 3:1, UV inactive on TLC, stains blue upon *p*-anisaldehyde staining) to give **22** as a pale yellow oil in 70% (24.6 mg). Spectroscopic and physical data were in accordance with literature reported values.[25c] $[\alpha]_D^{20}$ = +122.4 (c = 0.5, $CDCl_3$); ^{1}H NMR (500 MHz, $CDCl_3$): δ_H 6.20 (d, *J* = 3.4 Hz, 1H), 5.54 (brs, 1H), 5.47 (d, *J* = 3.1 Hz, 1H), 4.88 (d, *J* = 7.7 Hz, 2H), 4.04 (t, *J* = 9.6 Hz, 1H), 3.14 (dd, *J* = 13.6, 8.2 Hz, 1H), 2.85 (t, *J* = 7.9 Hz, 2H), 2.50 (ddd, *J* = 24.3, 14.9, 5.6 Hz, 2H), 2.39 (d, *J* = 16.4 Hz, 1H), 2.26 (ddd, *J* = 13.4, 9.5, 4.9 Hz, 1H), 2.16 – 2.09 (m, 1H), 1.85 (s, 3H), 1.44 (ddd, *J* = 15.7, 11.4, 5.3 Hz, 1H) ppm; ^{13}C NMR (125 MHz, $CDCl_3$): δ_C 170.3, 149.2, 140.0, 139.7, 126.6, 119.9, 113.0, 85.2, 56.1, 47.5, 45.9, 37.3, 35.5, 31.2, 16.8 ppm.

Direct comparison of proton and carbon shift values of synthesized isodehydrocostus lactone with previously reported synthesis.[25c]

Position	Proton Shifts synthesized	Proton Shifts of previously synthesized product	Carbon Shifts synthesized	Carbon Shifts of previously synthesized product
**1**	3.14	3.14	47.5	47.5
**2**			35.5	35.5
**3**	5.54	5.55	126.6	126.6
**4**	-	-	149.2	149.2
**5**	2.85	2.85	56.1	56.1
**6**	4.04	4.05	85.2	85.2
**7**	2.85	2.85	45.9	45.9
**8**			31.2	31.2
**9**			37.3	37.3
**10**	-	-	149.2	149.2
**11**	-	-	139.7	139.8
**12**	-	-	170.3	170.3
**13**	6.20 (a); 5.47 (b)	6.21 (a); 5.49 (b)	119.9	119.9
**14**	4.88	4.88	113.0	113.0
**15**	1.85	1.85	16.8	16.8

Synthesis of 3-deoxy-brachylaenolide (26)

To a sealed tube filled with argon, LiBr (14.6mg, 0.1685 mmol, 5eq.) and Li_2CO_3 (14.9mg, 0.2022mmol, 6eq.) were added. Reynosin mesylate (**18**, 11mg, 0.0337 mmol. 1eq.) was dissolved in DMF (dry, 1ml) and added to the tube. The resulting suspension was then stirred at 140°C (using an oil bath), for about 5 hours. After the completion of time, the reaction mixture was cooled to 0°C and neutralized, using 1N HCl (aq.) and was subsequently diluted with DCM and extracted with EtOAc. The combined organic layers were washed with H_2O, dried over $MgSO_4$, filtrated and evaporated under reduced pressure. The residue was purified by flash column chromatography on silica gel (eluent: hexane/Et_2O 14:1, TLC: R_f = 0.33 upon hexane/Et_2O 2:1, UV active on TLC, stains purple upon *p*-anisaldehyde staining) to give **26** as colorless gum in 66% (5.1 mg) yield. Spectroscopic and physical data were in accordance with literature reported values.[26] ($[\alpha]_D^{20}$ = −14.1 (c = 0.5, $CHCl_3$); ^{1}H NMR (500 MHz, $CDCl_3$): δ_H 6.09 (d, *J* = 3.1 Hz, 1H), 5.55 (brs, 2H), 5.40 (d, *J* = 2.9 Hz, 1H), 5.05 (brs, 1H), 4.93 (brs, 1H), 4.07 (t, *J* = 10.9 Hz, 1H), 2.93 (d, *J* = 20.2 Hz, 1H), 2.75 (d, *J* = 20.7 Hz, 1H), 2.59 (td, *J* = 11.2, 3.1 Hz, 1H), 2.53 (d, *J* = 11.0 Hz, 1H), 2.08 – 2.03 (m, 1H), 1.72 (dd, *J* = 8.4, 6.8 Hz, 1H), 1.67 – 1.58 (m, 2H), 0.89 (s, 3H) ppm; ^{13}C NMR (125 MHz, $CDCl_3$): δ_C 170.7, 141.1, 139.3, 137.5, 123.9, 116.8, 109.7, 79.6, 52.7, 50.0, 39.5, 37.2, 34.7, 21.5, 20.8 ppm.

Synthesis of Gazanolide (27)

To a sealed tube filled with argon, LiBr (66.8 mg, 0.7689 mmol, 5eq.) and Li_2CO_3 (68.2 mg, 0.9227 mmol, 6eq.) were added. Santamarine mesylate (**19**, 50.2 mg, 0.1537 mmol. 1eq.) dissolved in DMF (dry, 3.5 ml) was added to the tube. The resulting suspension was then stirred at 140°C (using an oil bath), overnight. After the completion of time, the reaction mixture was cooled to 0°C and neutralized, using 1N HCl (aq.) and subsequently diluted with DCM and extracted with EtOAc. The combined organic layers were washed with H_2O, dried over $MgSO_4$, filtrated and evaporated under reduced pressure. The residue was purified by flash column chromatography on silica gel (eluent: PS/Et_2O 20:1, TLC: R_f = 0.47 upon PS/Et_2O 5:1, UV active on TLC, stains light blue upon *p*-anisaldehyde staining) to give **27** as colorless amorphous solid in 57% (20.2 mg) yield. Spectroscopic and physical data were in accordance with literature reported values.[26][35] ($[\alpha]_D^{20}$ = +22.1 (c = 0.1, $CHCl_3$); ^{1}H NMR (500 MHz, $CDCl_3$): δ_H 6.08 (d, *J* = 2.7 Hz, 1H), 5.82 (dd, *J* = 9.3, 5.0 Hz, 1H), 5.72 (brs, 1H), 5.55 (d, *J* = 9.3 Hz, 1H), 5.41 (d, *J* = 2.6 Hz, 1H), 4.03 (t, *J* = 11.1 Hz, 1H), 2.67 (d, *J* = 11.5 Hz, 1H), 2.54 (td, *J* = 11.1, 3.0 Hz, 1H), 2.05 (dd, *J* = 12.7, 2.7 Hz, 1H), 2.00 (s, 3H), 1.74 – 1.61 (m, 3H), 0.92 (s, 3H) ppm; ^{13}C NMR (125 MHz, $CDCl_3$): δ_C 170.6, 138.8, 136.8, 136.0, 122.7, 120.1, 116.5, 81.1, 50.7, 50.6, 37.4, 36.5, 21.9, 21.3, 15.3 ppm.

Direct comparison of proton and carbon shift values of synthesized gazanolide with the reported isolation.[26, 35]

Position	Proton Shifts synthesized	Proton Shifts Isolation	Carbon Shifts synthesized	Carbon Shifts Isolation
**1**	5.55	5.55	120.1	120.2
**2**	5.82	5.82	136.8	136.9
**3**	5.72	5.72	122.7	122.7
**4**	-	-	136.0	135.9
**5**	2.67	2.68	50.7	50.7
**6**	4.03	4.03	81.1	81.1
**7**	2.54	2.54	50.7	50.7
**8**	2.05 (a); 1.61 – 1.74 (b)	2.03 (a); 1.62 (b)	21.3	21.3
**9**			36.5	36.5
**10**	-	-	37.4	37.4
**11**	-	-	138.8	138.8
**12**	-	-	170.6	170.5
**13**	6.08 (a); 5.41 (b)	6.09 (a); 5.40 (b)	116.5	116.5
**14**	0.92	0.92	15.3	15.3
**15**	2.00	2.00	21.9	21.9

6. Further oxidative decoration.

With ample quantities of Santamarine (**12**) and Reynosin (**11**) at our disposal, we envisioned the further enrichment, from an oxidative perspective, of the complexity of these *Eudesmanolide* congeners. Using our aerobic catalytic system for the epoxidation and the allylic oxidation of alkenes, we sought to access more heavily oxidized members of the family. In this paragraph we describe the syntheses of Santamarine acetate (**15**), acetyl Santamarine epoxide (**16**), Diacetyl-3α-hydroxyreynosin (**17**), Estafiatin (**23**), Isozaluzanin C (**25**), 5-hydroxy-Dehydrocostus lactone (**24**), 3-epi-Brachylaenolide (**28**) and Dehydro-α-santonin (**29**).

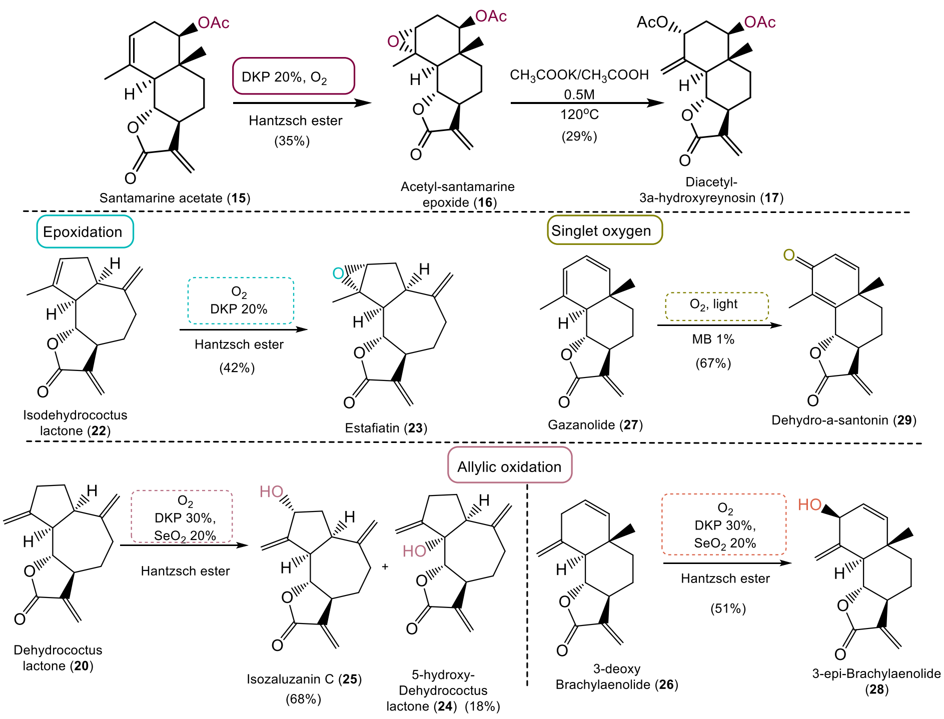

*Oxidation of Santamarine acetate (**15**)*

Considering that the free hydroxyl groups of **12** and **11** could interfere with our catalytic cycle, we envisaged masking the alcohol moiety at C-1 of these compounds with a protecting group. Eager to firstly test the theory of interference, we subjected **12** and **11** to the original conditions of DKP organocatalysis. Both epoxidation of Santamarine (**12**) and allylic oxidation of Reynosin (**11**) failed to deliver any product except for unreacted starting material. Santamarine and Reynosin mesylate **19** and **18** also met the same fate. It was then hypothesized that there were no oxidation products isolated due to the homoallylic interaction from the axial-positioned mesylate. The latter is not surprising considering the complete lack of reaction when allylic and homoallylic alcohols were tested in the seminal report. [9b]

After some experimentation, epoxidation of Santamarine acetate (**15**) was furnished by applying an excess of DKP catalyst and Hantzsch ester. This oxidation step provided, stereoselectively, the total synthesis of acetyl Santamarine epoxide (**16**), albeit in low yield (35%).

Preparation of Santamarine Acetate (15)

To a round bottom flask, equipped with a magnetic stirring bar, Santamarine (**12**, 19 mg, 0.0765 mmol, 1 eq.) was dissolved in pyridine (1.5 ml, 18.5 mmol, 242 eq.). Sequential addition of Ac_2O (0.15 ml, 1.58 mmol, 20.7 eq.) and DMAP (0.1 mg, 0.0007 mmol, 0.01 eq.) followed. The reaction mixture was then allowed to stir at room temperature for about 1 hour and 30 minutes. After the completion of time, the solution was diluted with DCM and extracted with water. The organic layer was washed with brine, dried over $MgSO_4$, filtrated and evaporated under reduced pressure. The residue was purified by flash column chromatography on silica gel (eluent: PS/Et_2O 5:1, TLC: R_f = 0.38 upon PS/Et_2O 2:1, UV active on TLC, stains blue-black upon p-anisaldehyde staining) to give **15** as white solid in 80% (17.7 mg) yield. Spectroscopic and physical data were in accordance with literature reported values.[14c] $[a]_D^{20}$ = +120.8 (c = 0.3, $CHCl_3$); mp 125–127°C ; ^1H NMR (500 MHz, $CDCl_3$): δ_H 6.08 (d, J = 3.1 Hz, 1H), 5.35 (brs, 1H), 5.40 (d, J = 3.1 Hz, 1H), 4.90 (dd, J = 9.9, 6.8 Hz, 1H), 3.92 (t, J = 11.0 Hz, 1H), 2.51 – 2.43 (m, 3H), 2.06 (s, 3H), 2.07 – 2.01 (m, 2H), 1.84 (s, 3H), 1.83 – 1.79 (m, 1H), 1.62 (ddd, J = 25.8, 13.0, 3.9 Hz, 1H), 1.35 – 1.28 (m, 1H), 0.97 (s, 3H) ppm; ^{13}C NMR (125 MHz, $CDCl_3$): δ_C 170.6, 138.8, 133.3, 120.9, 116.9, 81.1, 76.7, 50.9, 50.8, 39.7, 34.0, 29.4, 23.3, 21.0, 12.3 ppm.

Direct comparison of proton and carbon shift values of synthesized santamarine acetate with previously reported synthesis.[14c]

Position	Proton Shifts	Proton Shifts of	Carbon Shifts	Carbon Shifts of

	synthesized	previously synthesized product	synthesized	previously synthesized product
**1**	4.90	4.89	76.7	76.7
**2**	2.43 – 2.51	2.41 – 2.52	34.0	34.0
**3**	5.35	5.34	120.9	120.8
**4**	-	-	133.3	133.2
**5**	2.43 – 2.51	2.41 – 2.52	50.8	50.9
**6**	3.92	3.93	81.1	81.0
**7**	2.43 – 2.51	2.41 – 2-52	50.9	50.8
**8**	2.01 – 2.07 (a); 1.62 (b)	1.98 – 2.01 (a); 1.56 – 1.66 (b)	29.4	29.3
**9**	1.28 – 1.35 (a); 1.79 – 1.83 (b)	1.23 – 1.34 (a); 1.77 – 1.82 (b)	21.0	20.9
**10**	-	-	39.7	39.6
**11**	-	-	138.8	138.7
**12**	-	-	170.6	170.5
**13**	6.08 (a); 5.40 (b)	6.07 (a); 5.39 (b)	116.9	116.9
**14**	0.97	0.96	23.3	23.2
**15**	1.84	1.83	12.3	12.2
**other**	2.06	2.05	170.6, 21.0	170.5; 21.0

Preparation of Acetyl santamarine epoxide (16)

Santamarine acetate (**15**, 8 mg, 0.0275 mmol, 1 eq.) was dissolved in HFIP (2 ml) in a 4 ml screw capped vial with a stirring bar. Then, Hantzsch ester (**2**, 14 mg, 0.0551 mmol, 2 eq.) and DKP (**1**, 1.1 mg, 0.0055 mmol, 0.2 eq.) were added in one portion and the vial was tightly capped by a rubber septum. An exit needle and a pipette were introduced in the septum, through which dioxygen was bubbled continuously by a balloon. Care was taken to adjust slow bubbling in the reaction vial to avoid solvent's evaporation. The reaction was stirred at room temperature, under dioxygen atmosphere, overnight. After the completion of time, the reaction mixture was diluted with DCM and extracted consecutively with water and brine. The combined organic layers were dried over $MgSO_4$, filtrated and evaporated under reduced pressure. The residue was purified by flash column chromatography on silica gel (eluent: PS/EtOAc 3:1, TLC: R_f = 0.38 upon PS/EtOAc 2:1, UV inactive on TLC, stains greenish upon *p*-anisaldehyde staining) to give **16** as white solid in 35% (2.9 mg) yield. Spectroscopic and physical data were in accordance with literature reported values.[14a] ($[a]_D$ = +14; c=1.0, $CHCl_3$); mp 183–185°C; HRMS (ESI): m/z: $[M+H]^+$ calculated for $C_{17}H_{22}O_5H^+$ 307.1540; found: 307.1539; ^1H NMR (500 MHz, $CDCl_3$): δ_H 6.11 (brs, 1H), 5.42 (brs, 1H), 4.71 (dd, *J* = 9.0, 7.5 Hz, 1H), 3.89 (t, *J* = 11.2 Hz, 1H), 3.03 (brs, 1H), 2.53 (dd, *J* = 15.3, 6.7 Hz, 1H), 2.50 – 2.44 (m, 1H), 2.05 (s, 5H), 1.86 – 1.79 (m, 1H), 1.70 (d, *J* = 13.8 Hz, 1H), 1.54 – 1.61 (m, 2H), 1.49 (s, 3H), 1.01 (s, 3H) ppm;, ^{13}C NMR (125 MHz, $CDCl_3$): δ_C 170.1, 170.0, 138.3, 117.5, 80.3, 75.4, 60.4, 57.0, 52.5, 50.3, 39.2, 33.9, 29.7, 27.9, 21.5, 21.0,12.9 ppm.

Synthesis of Diacetyl-3α-hydroxyreynosin (17)

Acetyl santamarine epoxide (**16**, 13 mg, 0.0424 mmol, 1 eq.) was dissolved in a solution of 0.5M CH_3COOH/CH_3COOK (55.4 mg, 13 eq. of CH_3COOK dissolved in 1.13ml of CH_3COOH), in a sealed tube equipped with a magnetic stirring bar. The reaction mixture was then immersed in an oil bath and heated up to 120°C, where it was allowed to stir overnight. After the completion of time, the reaction mixture was quenched with brine and extracted with EtOAc. The combined organic layers were dried over $MgSO_4$, filtrated and evaporated under reduced pressure and heat. The residue was purified by flash column chromatography on silica gel (eluent: hexane/EtOAc 3:1, TLC: R_f = 0.48 upon PS/EtOAc 2:1, UV inactive on TLC, stains purple upon *p-anisaldehyde* staining) to give **17** as colorless oil in 29% (4.3 mg) yield. Spectroscopic and physical data were in accordance with literature reported values.[22] $[a]_D^{20}$ = +57.5 (c = 0.1, $CHCl_3$,; HRMS (ESI): m/z: $[M+H]^+$ calculated for $C_{19}H_{24}O_6H^+$ 349.1646; found: 349.1646; ^1H NMR (500 MHz, $CDCl_3$): δ_H 6.11 (brs, 1H), 5.45 (d, *J* = 16.4 Hz, 2H), 5.34 (brs, 1H), 5.16 (brs, 1H), 5.11 (dd, *J* = 12.4, 4.5 Hz, 1H), 3.99 (t, *J* = 10.5 Hz, 1H), 2.71 (d, *J* = 11.0 Hz, 1H), 2.64 – 2.57 (m, 1H), 2.07 (d, *J* = 9.6 Hz, 7H), 1.81 (d, *J* = 12.2 Hz, 2H), 1.61 (d, *J* =

11.7 Hz, 2H), 1.47 (d, *J* = 11.6 Hz, 1H), 0.90 (s, 3H) ppm; ^{13}C NMR (125 MHz, $CDCl_3$): δ_C 170.3, 169.8, 139.3, 138.7, 117.5, 116.7, 78.7, 75.2, 73.7, 49.4, 48.5, 41.9, 35.0, 32.7, 21.5, 21.2, 21.1, 12.1 ppm.

*Synthesis of Estafiatin (**23**), Isozaluzanin C (**25**), 5-hydroxy-Dehydrocostus lactone (**24**) and 3-epi-Brachylaenolide (**28**)*

Inspired by the successful conversion of Santamarine acetate (**15**) to its epoxidized derivative (**16**), we sought to apply the same rationale in the oxidation of the newly prepared guaiane carbocycles **20**, **22** and the *Eudesmanolide* **26**. Performing epoxidation and allylic oxidation with the aid of higher quantities of DKP (20-30%) on **22** and **20**, resulted in the total synthesis of Estafiatin (**23**) in 40% yield, and a mixture of Isozaluzanin C (**25**) and 5-hydroxy Dehydrocostus lactone (**24**) in 68% and 18% yields respectively, while subjecting **26** to allylic oxidation conditions returned stereoselectively 3-epi-Brachylaenolide (**28**) in 51% yield.

Synthesis of Estafiatin (23)

Isodehydrocostus lactone (**22**, 28 mg, 0.1215 mmol, 1 eq.) was dissolved in HFIP (2 ml) in a 4 ml screw capped vial with a stirring bar. Then, Hantzsch ester (**2**, 61.6 mg, 0.2431 mmol, 2 eq.) and DKP (**1**, 4.6 mg, 0.0243 mmol, 0.2 eq.) were added in one portion and the vial was tightly capped by a rubber septum. An exit needle and a pipette were introduced in the septum, through which dioxygen was bubbled continuously by a balloon. Care was taken to adjust slow bubbling in the reaction vial to avoid solvent's evaporation. The reaction was stirred at room temperature, under dioxygen atmosphere, overnight. After the completion of time, the reaction mixture was diluted with DCM and extracted consecutively with water and brine. The combined organic layers were dried over $MgSO_4$, filtrated and evaporated under reduced pressure. The residue was purified by flash column chromatography on silica gel (eluent: hexane/Et_2O 2:1, TLC: R_f = 0.37 upon hexane/Et_2O 1:1, UV inactive on TLC, stains purple upon *p*-anisaldehyde staining) to give **23** as colorless needles in 42% (12.6 mg) yield. Spectroscopic and physical data were in accordance with literature reported values.[36] $[a]_D^{20}$ = -13.5 (c = 0.1, $CHCl_3$); mp 105–107°C; HRMS (ESI): m/z: $[M+H]^+$ calculated for $C_{15}H_{18}O_3H^+$ 247.1329; found:247.1330 ; ^{1}H NMR (500 MHz, $CDCl_3$): δ_H 6.21 (d, *J* = 3.5 Hz, 1H), 5.49 (d, *J* = 3.1 Hz, 1H), 4.95 (brs, 1H), 4.86 (brs, 1H), 4.08 (dd, *J* = 10.7, 9.1 Hz, 1H), 3.38 (brs, 1H), 2.98 (dd, *J* = 18.4, 8.1 Hz, 1H), 2.90 – 2.82 (m, 1H), 2.34 – 2.25 (m, 1H), 2.24-2.16 (m, 3H), 2.07 (dd, *J* = 14.0, 7.5 Hz, 1H), 1.86 – 1.78 (m, 1H), 1.62 (s, 3H), 1.55 – 1.50 (m, 1H) ppm; ^{13}C NMR (125 MHz, $CDCl_3$): δ_C 169.8, 146.0, 139.5, 120.3, 115.3, 80.5, 65.9, 63.2, 50.8, 44.8, 44.0, 32.9, 29.2, 28.5,18.5 ppm.

Direct comparison of proton and carbon shift values of synthesized estafiatin with the reported isolation.[36]

Position	Proton Shifts synthesized	Proton Shifts Isolation	Carbon Shifts synthesized	Carbon Shifts Isolation
**1**	2.98	2.98	44.8	44.9
**2**	2.07 (a); 1.81 (b)	2.07 (a); 1.81 (b)	32.9	33.0
**3**	3.38	3.38	63.2	63.2
**4**	-	-	65.9	65.8
**5**	2.30	2.32	50.8	50.8
**6**	4.08	4.08	80.5	80.5
**7**	2.87	2.87	44.0	44.1
**8**	2.20 (a); 1.53 (b)	2.22 (a); 1.53 (b)	29.2	29.2
**9**	2.30 (a); 2.20 (b)	2.28 (a); 2.19 (b)	28.5	28.6
**10**	-	-	146.0	146.1
**11**	-	-	139.5	139.6
**12**	-		169.8	169.7
**13**	6.21 (a); 5.49 (b)	6.21 (a); 5.48 (b)	120.3	120.2
**14**	4.95 (a); 4.86 (b)	4.95 (a); 4.86 (b)	115.3	115.3
**15**	1.62	1.62	18.5	18.5

Synthesis of Isozaluzanin C (25) and 5-hydroxy-dehydrocostus lactone (24)

Dehydrocostus lactone (**20**, 20 mg, 0.0868 mmol, 1eq.) was dissolved in HFIP (2 ml) in a 4 ml screw capped vial with a stirring bar. Then, Hantzsch ester (**2**, 66 mg, 0.2605 mmol, 3 eq.), DKP (**1**, 4.9 mg, 0.0260 mmol, 0.3 eq.) and SeO_2 (1.9 mg, 0.0173 mmol, 0.2 eq.) were added in one portion and the vial was tightly capped by a rubber septum. An exit needle and a pipette were introduced in the septum, through which dioxygen was bubbled continuously by a balloon. Care was taken to adjust slow bubbling in the reaction vial to avoid solvent's evaporation. The reaction was stirred at room temperature, under dioxygen atmosphere, overnight. After the completion of time, the reaction mixture was diluted with DCM and extracted consecutively with water and brine. The combined organic layers were dried over $MgSO_4$, filtrated and evaporated under reduced pressure. The residue was purified by flash column chromatography on silica gel to give Isozaluzanin C (**25**) (eluent: PS/EtOAc 3:1, TLC: R_f = 0.11 upon PS/EtOAc 3:1, UV inactive on TLC, stains brown upon *p*-anisaldehyde staining, physical state: colorless crystals) and 5-hydroxy-dehydrocostus lactone (**24**) (eluent: PS/EtOAc 8:1, TLC: R_f = 0.43 upon PS/EtOAc 3:1, UV inactive on TLC, stains light brown-grey upon *p*-anisaldehyde staining, physical state: colorless amorphous solid) in 68% (14.5 mg) and 18% (3.8 mg) yield respectively. Spectroscopic and physical data were in accordance with literature reported values.[37,25a] **For Isozaluzanin C (25):** $[\alpha]_D^{20}$ = - 50.2(c = 0.5,$CHCl_3$); mp 143–145°C; ^{1}H NMR (500 MHz, $CDCl_3$): δ_H 6.21 (d, *J* = 3.4 Hz, 1H), 5.50 – 5.48 (m, 2H), 5.35 (brs, 1H), 4.92 (brs, 1H), 4.77 (brs, 1H), 4.68 (t, *J* = 6.0 Hz, 1H), 3.89 (t, *J* = 9.1 Hz, 1H), 3.11 – 3.07 (m, 2H), 2.88 – 2.81 (m, 1H), 2.51 (dt, *J* = 12.6, 4.6 Hz, 1H), 2.24 – 2.16 (m, 2H), 2.14 – 2.07 (m, 1H), 1.89 – 1.83 (m, 1H), 1.42 – 1.34 (m, 1H) ppm; ^{13}C NMR (125 MHz, $CDCl_3$): δ_C 170.1, 154.1, 148.5, 139.4, 120.5, 113.3, 113.2, 84.9, 74.6, 49.6, 45.6, 44.2, 39.9, 36.7, 30.9 ppm. **For 5-hydroxy-dehydrocostus lactone (24):** ($[\alpha]_D^{20}$ = - 33.5 (c = 0.5, $CHCl_3$); ^{1}H NMR (300 MHz, $CDCl_3$): δ_H 6.24 (d, *J* = 3.6 Hz, 1H), 5.52 (d, *J* = 3.2 Hz, 1H), 5.41 (t, *J* = 2.4 Hz, 1H), 5.10 (t, *J* = 2.2 Hz, 1H), 4.92 (brs, 1H), 4.82 (brs, 1H), 4.18 (d, *J* = 8.8 Hz, 1H), 3.25 (tq, *J* = 8.7, 3.3 Hz, 1H), 2.68 (dd, *J* = 8.4, 3.6 Hz, 2H), 2.61 – 2.54 (m, 1H), 2.52 – 2.45 (m, 1H), 2.8 – 2.18 (m, 4H), 1.82 (ddd, *J* = 18.4, 9.2, 4.6 Hz, 1H), 1.46 – 1.41 (m, 1H) ppm; ^{13}C NMR (75 MHz, $CDCl_3$): δ_C 170.2, 154.1, 148.1, 139.4, 120.9, 113.6, 110.6, 85.7, 81.1, 55.4, 40.2, 37.0, 31.6, 31.3, 28.2 ppm.

Direct comparison of proton and carbon shift values of synthesized isozaluzanin with previously reported synthesis.[37]

Position	Proton Shifts synthesized	Proton Shifts of previously synthesized product	Carbon Shifts synthesized	Carbon Shifts of previously synthesized product
**1**	3.10	3.08	49.6	49.6
**2**	2.20 (a); 1.86 (b)	2.16 (a); 1.85 (b)	36.7	36.6
**3**	4.68	4.62	74.6	74.6
**4**	-	-	154.1	154.3
**5**	3.10	3.08	45.6	45.6
**6**	3.89	3.89	84.9	84.9
**7**	2.84	2.84	44.2	44.2
**8**	2.10 (a); 1.37 (b)	2.08 (a); 1.37 (b)	30.9	31.3
**9**	2.51 (a); 2.20 (b)	2.51 (a); 2.21 (b)	39.9	39.8
**10**	-	-	139.4	139.6
**11**	-	-	148.5	148.6
**12**	-	-	170.1	169.7
**13**	5.35 (a); 6.21(b)	5.30 (a); 6.21 (b)	120.5	120.5
**14**	4.92 (a); 4.77 (b)	4.92 (a); 4.77 (b)	113.2	113.2
**15**	5.49 (a);(b)	5.49 (a); 5.34 (b)	113.3	113.3

Direct comparison of proton and carbon shift values of synthesized 5-hydroxy-dehydrocostus lactone with previously reported synthesis.[25a]

Position	Proton Shifts synthesized	Proton Shifts Isolation	Carbon Shifts synthesized	Carbon Shifts Isolation
**1**	2.68	2.69	55.4	55.4
**2**	1.82 (a); 2.20 (b)	1.82 (a); 2.19 (b)	28.2	28.2

**3**	2.57 (a); (b) 2.68 (b)	2.57 (a); 2.77 (b)	31.3	31.2
**4**	-	-	154.1	154.2
**5**	-	-	81.1	81.2
**6**	4.18	4.18	85.7	85.8
**7**	3.25	3.26	40.2	40.2
**8**	2.20 (a); 1.44 (b)	2.19 (a); 1.45 (b)	31.6	31.5
**9**	2.25 (a); 2.49 (b)	2.24 (a); 2.48 (b)	37.0	37.0
**10**	-	-	139.4	139.5
**11**	-	-	148.1	148.2
**12**	-	-	170.2	170.9
**13**	5.52 (a); 6.24 (b)	5.52 (a); 6.24 (b)	120.9	121.0
**14**	4.92 (a); 4.82 (b)	4.92 (a); 4.82 (b)	110.6	110.7
**15**	5.41 (a); 5.10 (b)	5.41 (a); 5.10 (b)	113.6	113.7

Synthesis of 3-epi-Brachylaenolide (28)

3-deoxy-Brachylaenolide (**26**, 12 mg, 0.0521 mmol, 1eq.) was dissolved in HFIP (2 ml) in a 4 ml screw capped vial with a stirring bar. Then, Hantzsch ester (**2**, 39.6 mg, 0.1563 mmol, 3 eq.), DKP (**1**, 3 mg, 0.0156 mmol, 0.3 eq.) and SeO_2 (1.2 mg, 0.0104 mmol, 0.2 eq.) were added in one portion and the vial was tightly capped by a rubber septum. An exit needle and a pipette were introduced in the septum, through which dioxygen was bubbled continuously by a balloon. Care was taken to adjust slow bubbling in the reaction vial to avoid solvent's evaporation. The reaction was stirred at room temperature, under dioxygen atmosphere, overnight. Upon completion, the reaction mixture was diluted with DCM and extracted consecutively with water and brine. The combined organic layers were dried over $MgSO_4$, filtrated and evaporated under reduced pressure. The residue was purified by flash column chromatography on silica gel (eluent: PS/Et_2O 1:1, TLC: R_f = 0.40 upon PS/Et_2O 1:3, UV active on TLC, stains blue-black upon *p*-anisaldehyde staining) to give **28** as colorless crystals in 51% (6.5 mg) yield. Spectroscopic and physical data were in accordance with literature reported values.[27b] $[\alpha]_D^{20}$ = +122 (c = 0.1, $CDCl_3$); mp 141–143°C; ^{1}H NMR (500 MHz, $CDCl_3$): δ_H 6.09 (t, *J* = 3.0 Hz, 1H), 5.76 (dd, *J* = 9.9, 2.4 Hz, 1H), 5.71 (dt, *J* = 9.6, 3.2 Hz, 1H), 5.41 (t, *J* = 3.0 Hz, 1H), 5.32 (brs, 1H), 5.15 (brs, 1H), 4.43 (brs, 1H), 4.05 (td, *J* = 11.0, 3.0 Hz, 1H), 2.82 (d, *J* = 11.3 Hz, 1H), 2.65 – 2.58 (m, 1H), 2.11 – 2.04 (m, 1H), 1.75 – 1.60 (m, 3H), 0.84 (s, 3H) ppm; ^{13}C NMR (125 MHz, $CDCl_3$): δ_C 170.6, 144.8, 141.4, 139.1, 125.4, 117.1, 113.9, 78.9, 69.2, 50.0, 48.6, 40.2, 36.6, 21.4, 19.5 ppm.

*Combining DKP aerobic oxidations with the established synthetic ability of singlet oxygen chemistry-Synthesis of dehydro-α-Santonin (**29**)*

The combination of DKP's catalytic system along with singlet oxygen chemistry, further extended the borders of its synthetic potential. When previously prepared Gazaniolide (**27**) was treated with methylene blue and dioxygen under regular light cleanly provided Dehydro-α-santonin (**29**) in 67% yield.

Synthesis of Dehydro-α-santonin (29)

Gazaniolide (**27**, 10 mg, 0.0434 mmol, 1eq.) was dissolved in $CDCl_3$ (4 ml) in a 10 ml screw capped vial with a stirring bar. Methylene blue was added and the vial was tightly capped by a rubber septum. An exit needle and a pipette were introduced in the septum, through which dioxygen was bubbled continuously by a balloon. Care was taken to adjust slow bubbling in the reaction vial to avoid solvent's evaporation. The reaction was stirred at a cooling bath (0°C), under dioxygen atmosphere and 10 cm away from a light source (white led lamp, SYLVANIA, 2550Lm, 6500K, 220-240V, 50/60Hz, 20W) for about 10 minutes. After completion, the reaction mixture was quenched with dimethyl sulfide and left stirring for 1h. Evaporation under reduced pressure followed. The residue was purified by flash column chromatography on silica gel (eluent: hexane/EA 1:1, TLC: R_f = 0.41 upon hexane/EA 1:1, UV active on TLC, stains light purple upon p-anisaldehyde staining) to give **29** as colorless crystals in 67% (7.1 mg) yield. Spectroscopic

and physical data were in accordance with literature reported values.[38] $[\alpha]_D^{20}$ = –62 (c = 0.5, EtOH); mp 145–147°C; ^{1}H NMR (300 MHz, $CDCl_3$): δ_H 6.70 (d, *J* = 9.8 Hz, 1H), 6.27 (d, *J* = 10.0 Hz, 1H), 6.25 (d, *J* = 1.5 Hz, 1H), 5.56 (brs, 1H), 4.77 (d, *J* = 11.5 Hz, 1H), 2.70 (t, *J* = 10.7 Hz, 1H), 2.16 (s, 3H), 1.93 (d, *J* = 13.6 Hz, 1H), 1.78 (ddd, *J* = 25.7, 12.7, 3.4 Hz, 1H), 1.63 – 1.58 (m, 2H), 1.32 (s, 3H) ppm; ^{13}C NMR (125 MHz, $CDCl_3$): δ_C 186.2, 169.1, 154.6, 150.6, 137.5, 129.0, 126.0, 119.7, 81.4, 50.3, 41.3, 37.6, 25.2, 21.7, 10.8 ppm.

7. Copies of NMR spectra

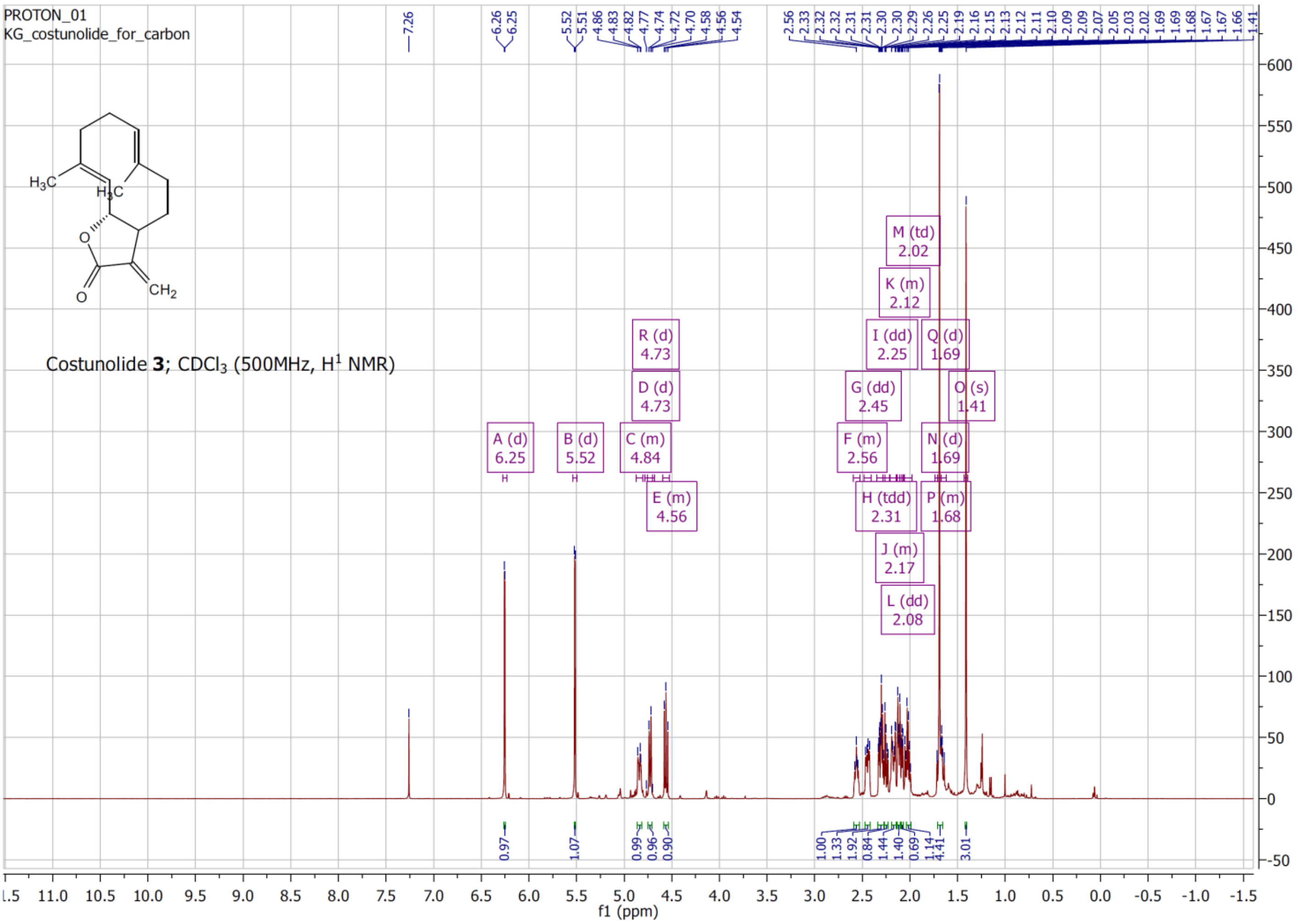
PROTON_01
KG_costunolide_for_carbon
Costunolide 3; CDCl3 (500MHz, H1 NMR)
f1 (ppm)

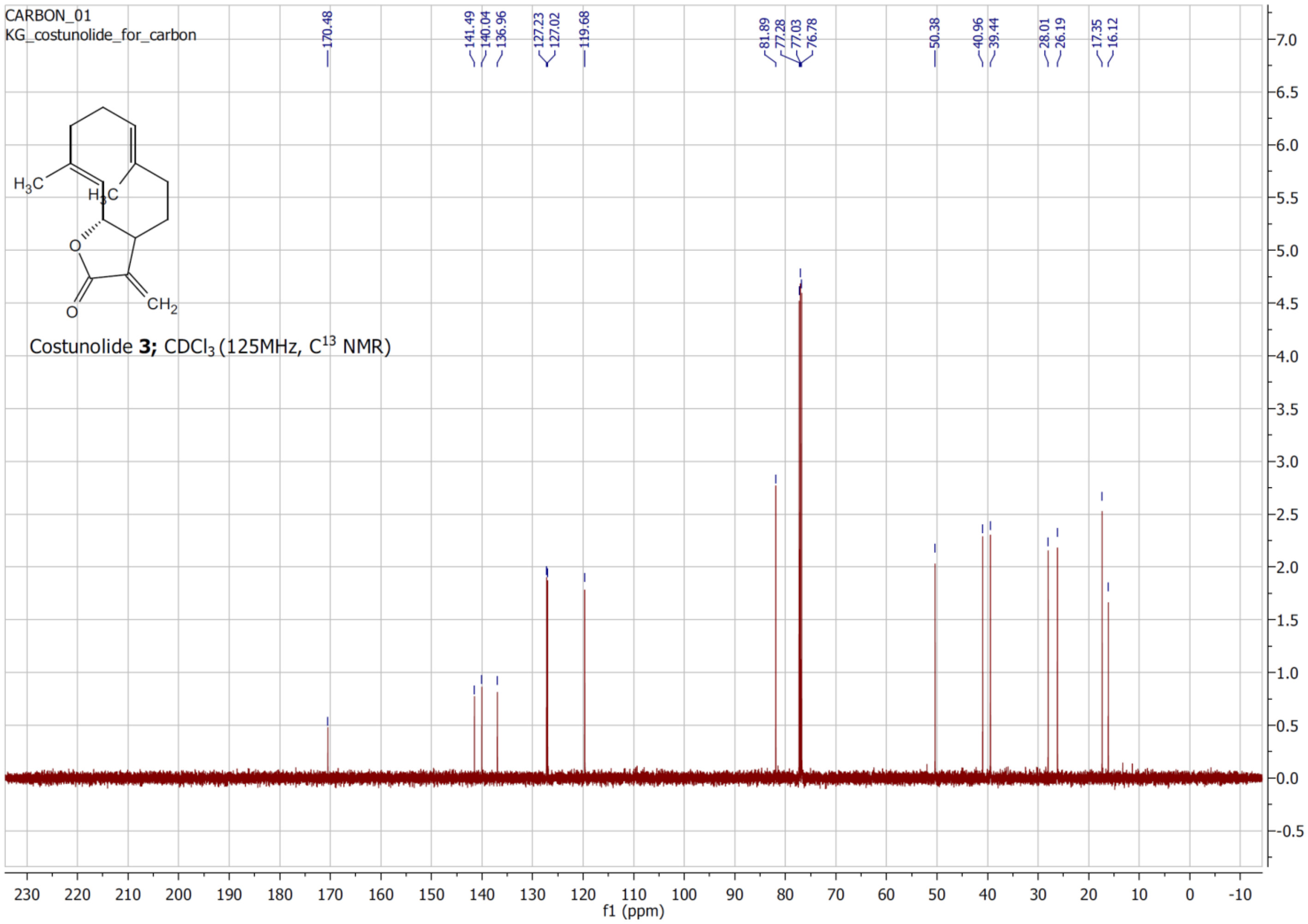
CARBON_01
KG_costunolide_for_carbon
Costunolide 3; CDCl3 (125MHz, C13 NMR)
f1 (ppm)

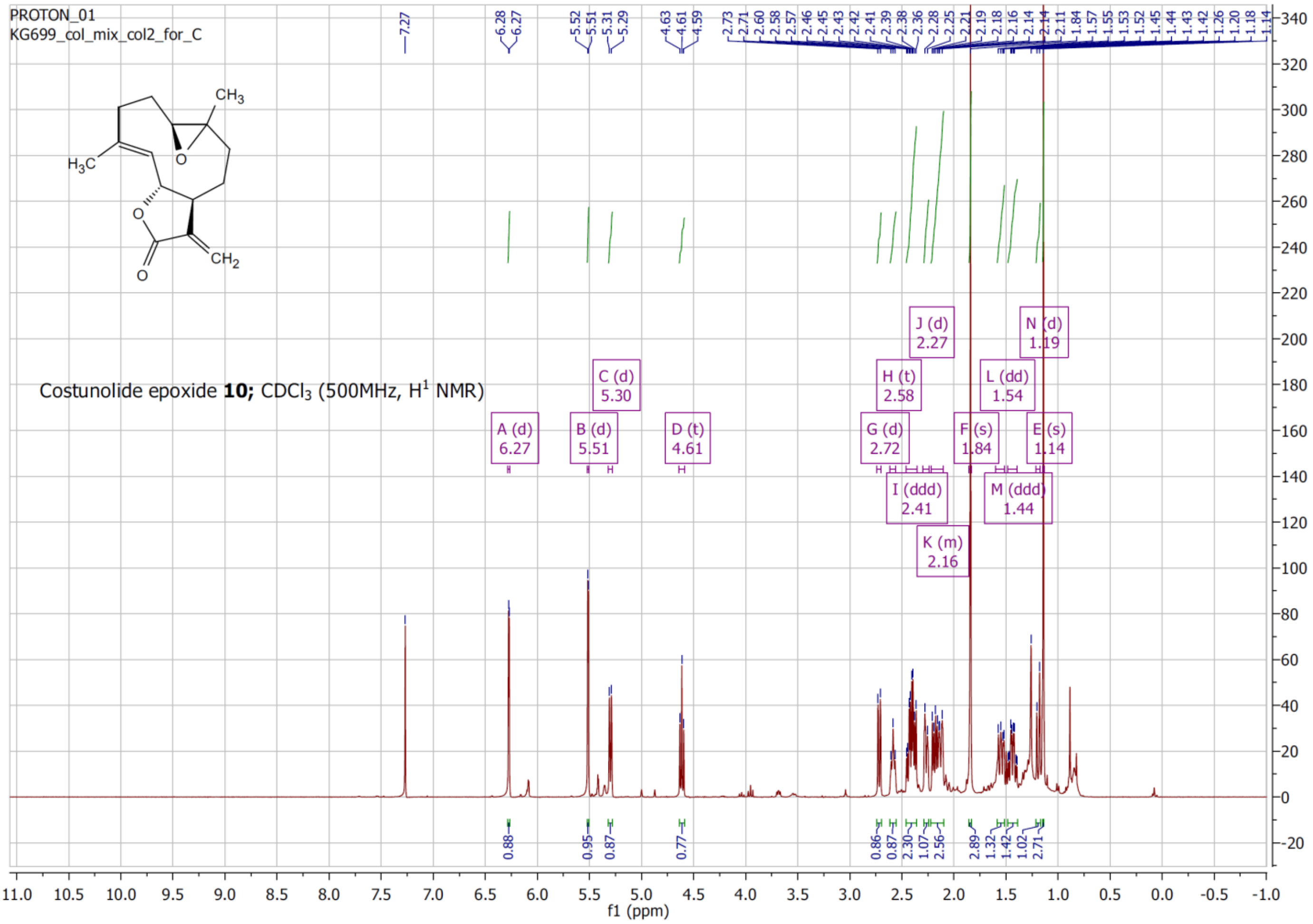
PROTON_01
KG699_col_mix_col2_for_C
7.27
6.28
6.27
5.52
5.51
5.31
5.29
4.63
4.61
4.59
2.73
2.71
2.60
2.58
2.57
2.46
2.45
2.43
2.42
2.41
2.39
2.38
2.36
2.28
2.25
2.21
2.19
2.18
2.16
2.14
2.14
2.11
1.84
1.57
1.55
1.53
1.52
1.45
1.44
1.43
1.42
1.26
1.20
1.18
1.14
CH3
H3C
O
O
O
CH2
Costunolide epoxide 10; CDCl3 (500MHz, H1 NMR)
A (d) 6.27
B (d) 5.51
C (d) 5.30
D (t) 4.61
G (d) 2.72
H (t) 2.58
I (ddd) 2.41
J (d) 2.27
K (m) 2.16
F (s) 1.84
L (dd) 1.54
M (ddd) 1.44
E (s) 1.14
N (d) 1.19
0.88
0.95
0.87
0.77
0.86
0.87
2.30
1.07
2.56
2.89
1.32
1.42
1.02
2.71
f1 (ppm)

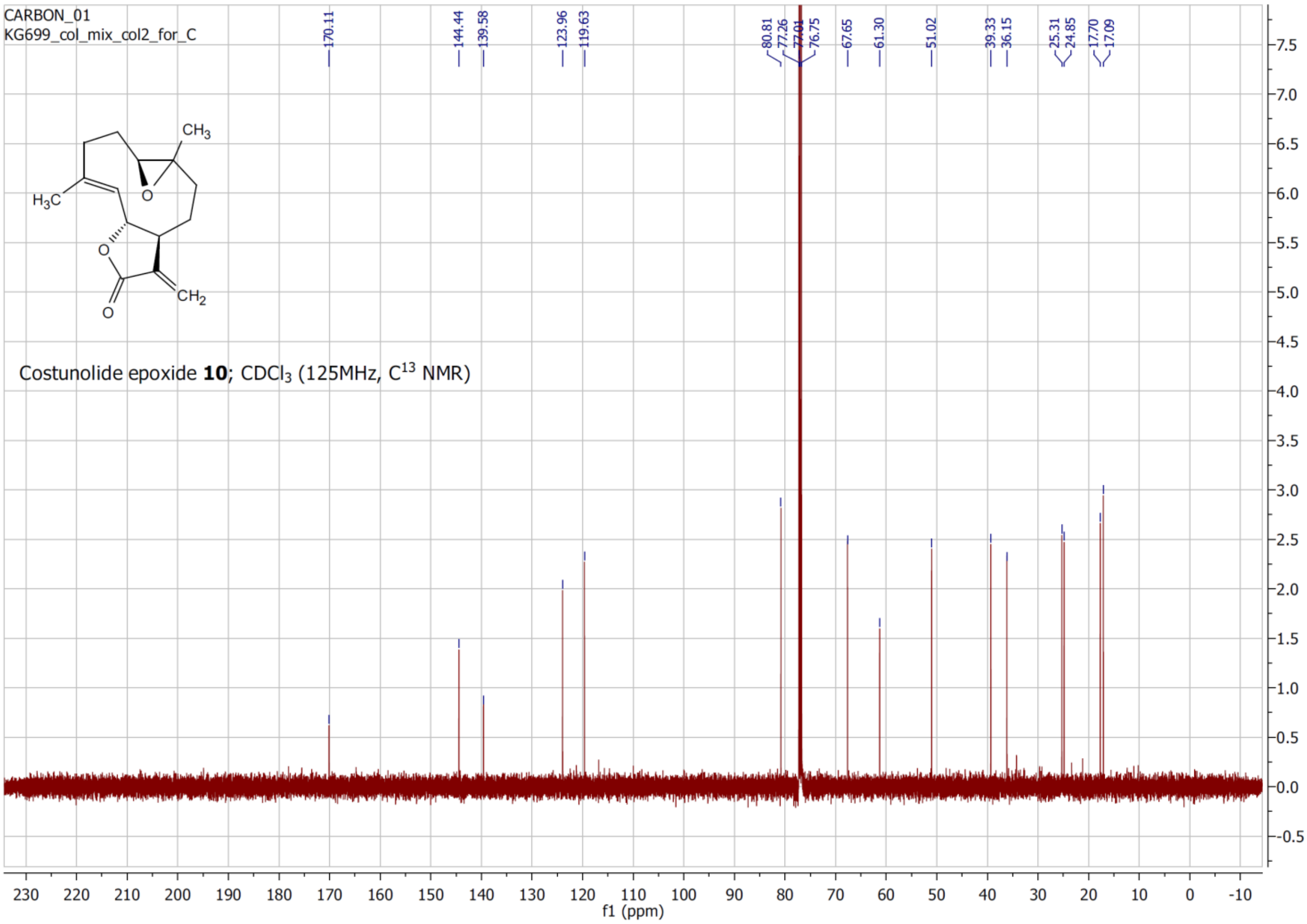
CARBON_01
KG699_col_mix_col2_for_C
170.11
144.44
139.58
123.96
119.63
80.81
77.26
77.01
76.75
67.65
61.30
51.02
39.33
36.15
25.31
24.85
17.70
17.09
CH3
H3C
O
O
O
CH2
Costunolide epoxide 10; CDCl3 (125MHz, C13 NMR)
f1 (ppm)

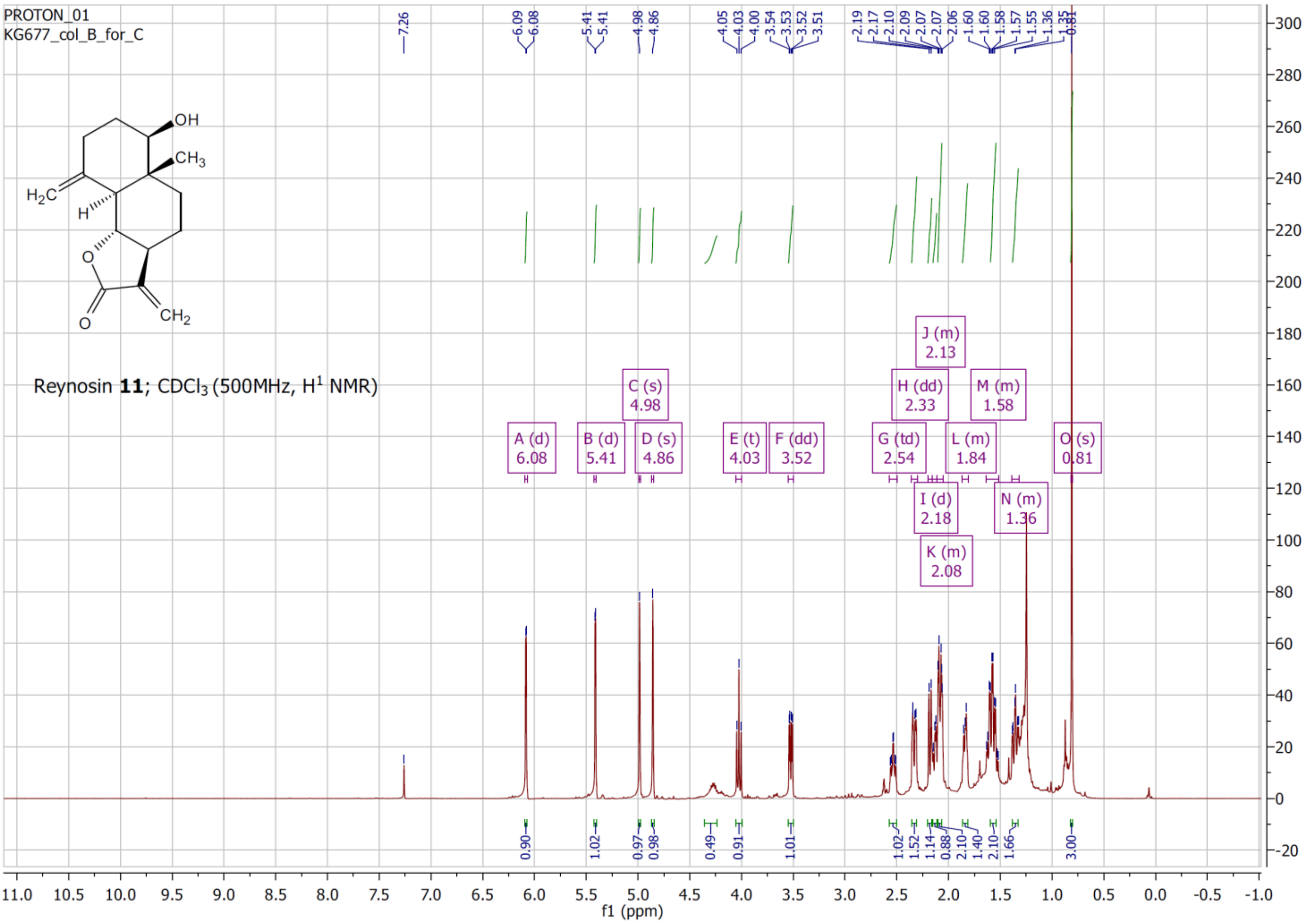
PROTON_01
KG677_col_B_for_C
Reynosin **11**; $CDCl_3$ (500MHz, H[1] NMR)
7.26
6.09 6.08
5.41 5.41
4.98 4.86
4.05 4.03 4.00 3.54 3.53 3.52 3.51
2.19 2.17 2.10 2.09 2.07 2.07 2.06 1.60 1.60 1.58 1.57 1.55 1.36 1.35 0.81
A (d) 6.08
B (d) 5.41
C (s) 4.98
D (s) 4.86
E (t) 4.03
F (dd) 3.52
G (td) 2.54
H (dd) 2.33
I (d) 2.18
J (m) 2.13
K (m) 2.08
L (m) 1.84
M (m) 1.58
N (m) 1.36
O (s) 0.81
0.90 1.02 0.97 0.98 0.49 0.91 1.01 1.02 1.52 1.14 0.88 2.10 1.40 2.10 1.66 3.00
f1 (ppm)

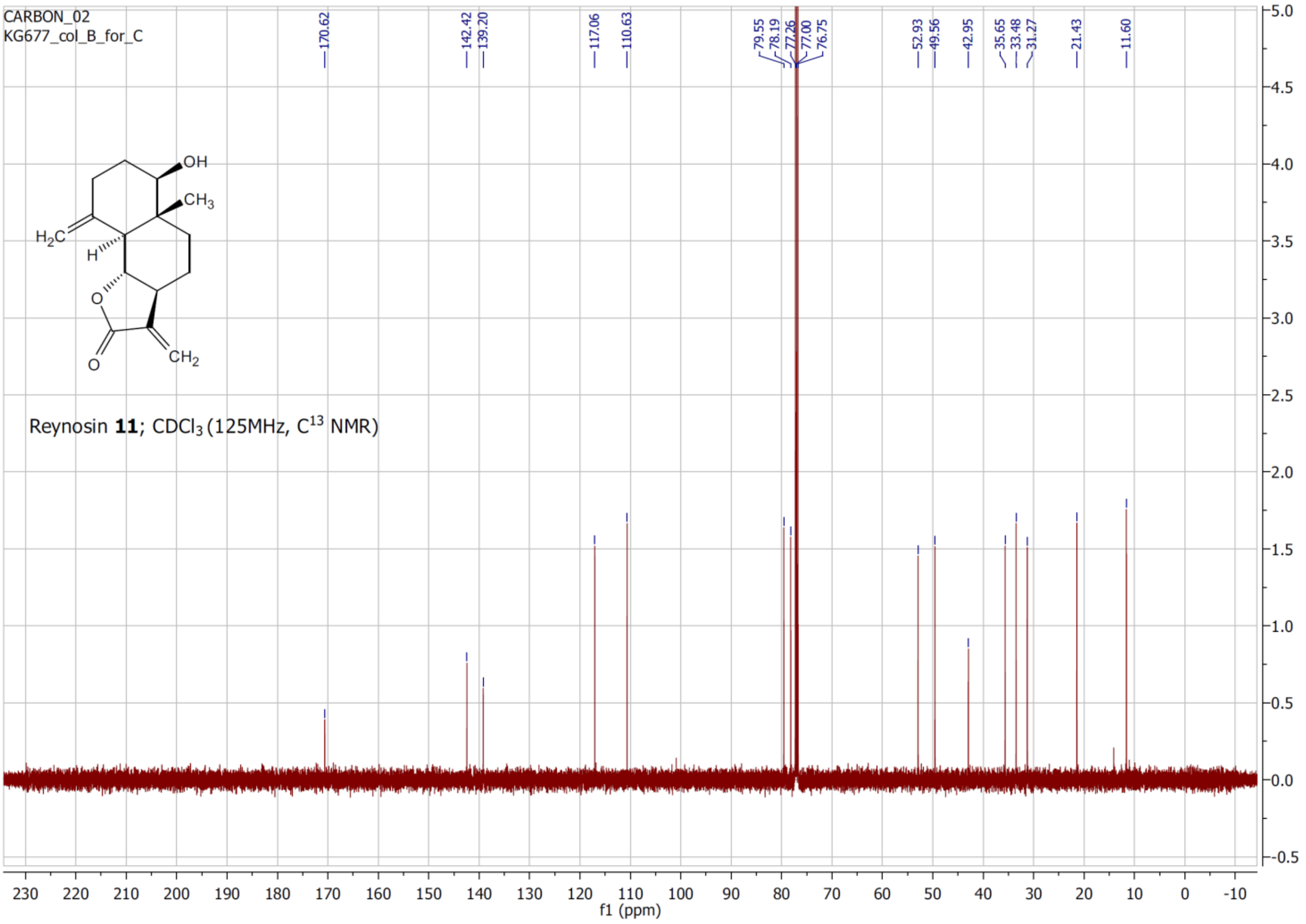
CARBON_02
KG677_col_B_for_C
Reynosin **11**; $CDCl_3$ (125MHz, C[13] NMR)
170.62
142.42 139.20
117.06 110.63
79.55 78.19 77.26 77.00 76.75
52.93 49.56 42.95 35.65 33.48 31.27 21.43 11.60
f1 (ppm)

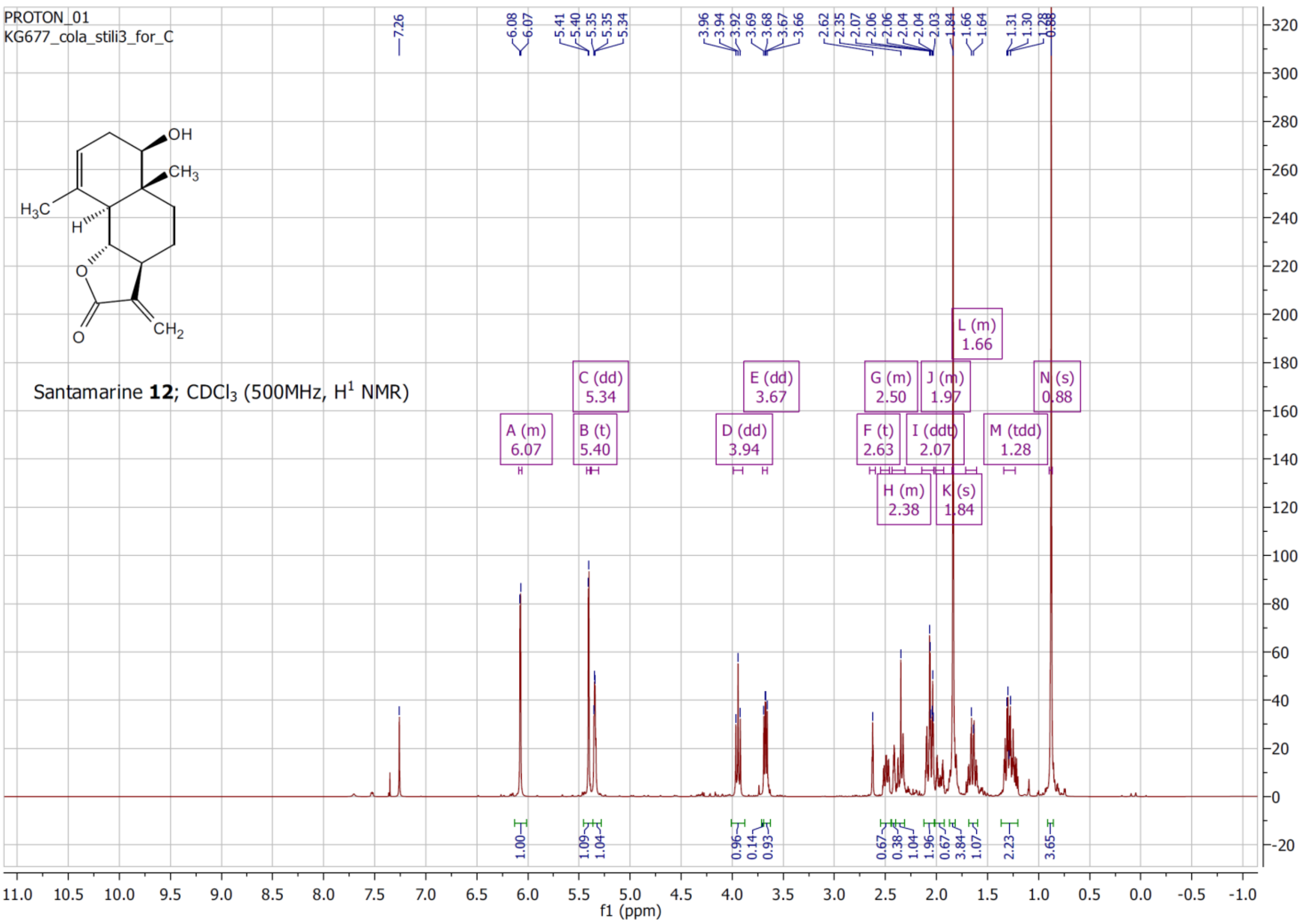
PROTON_01
KG677_cola_stili3_for_C
Santamarine 12; CDCl3 (500MHz, H1 NMR)
A (m) 6.07
B (t) 5.40
C (dd) 5.34
D (dd) 3.94
E (dd) 3.67
F (t) 2.63
G (m) 2.50
H (m) 2.38
I (ddt) 2.07
J (m) 1.97
K (s) 1.84
L (m) 1.66
M (tdd) 1.28
N (s) 0.88
f1 (ppm)

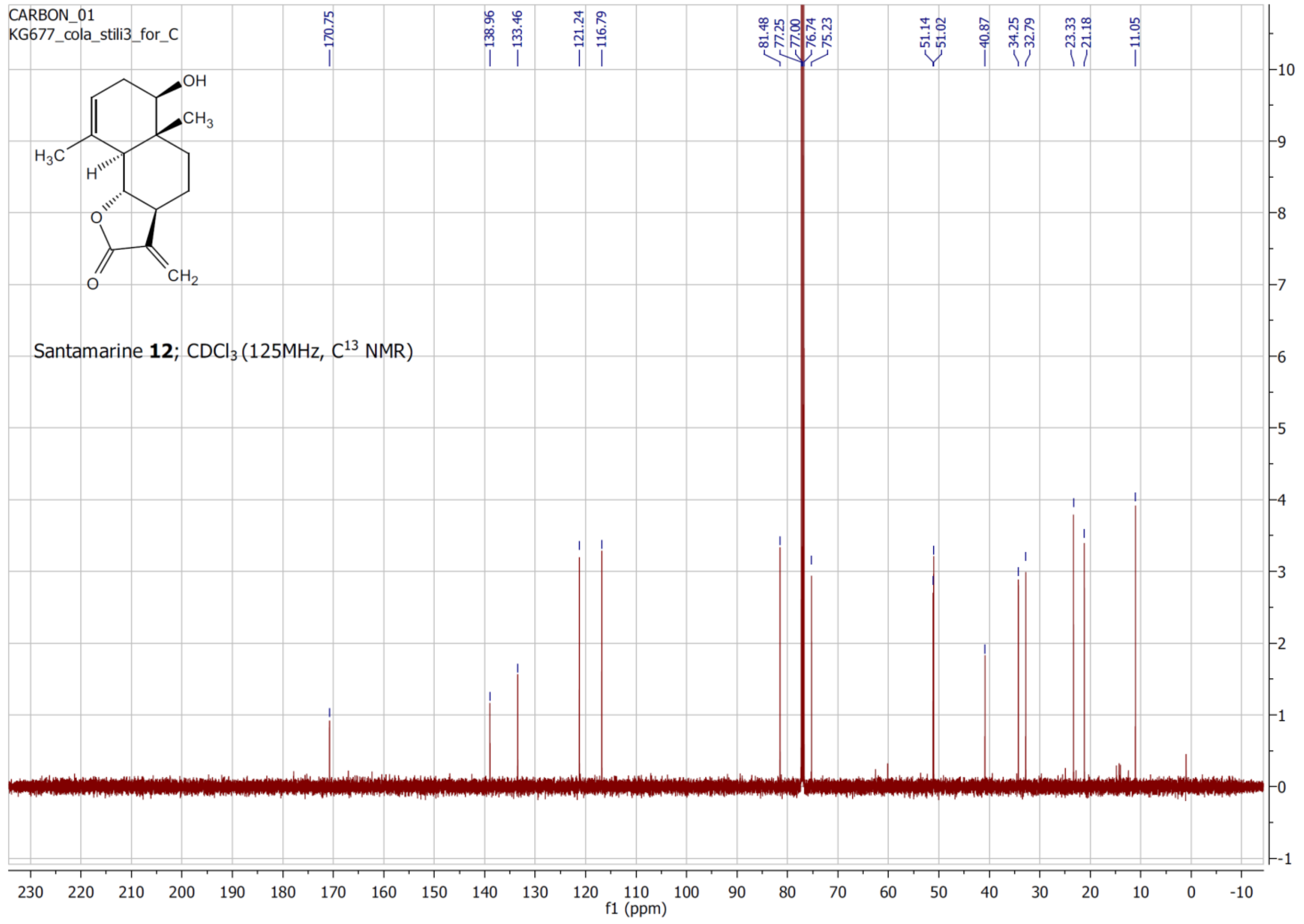
CARBON_01
KG677_cola_stili3_for_C
Santamarine 12; CDCl3 (125MHz, C13 NMR)
170.75
138.96
133.46
121.24
116.79
81.48
77.25
77.00
76.74
75.23
51.14
51.02
40.87
34.25
32.79
23.33
21.18
11.05
f1 (ppm)

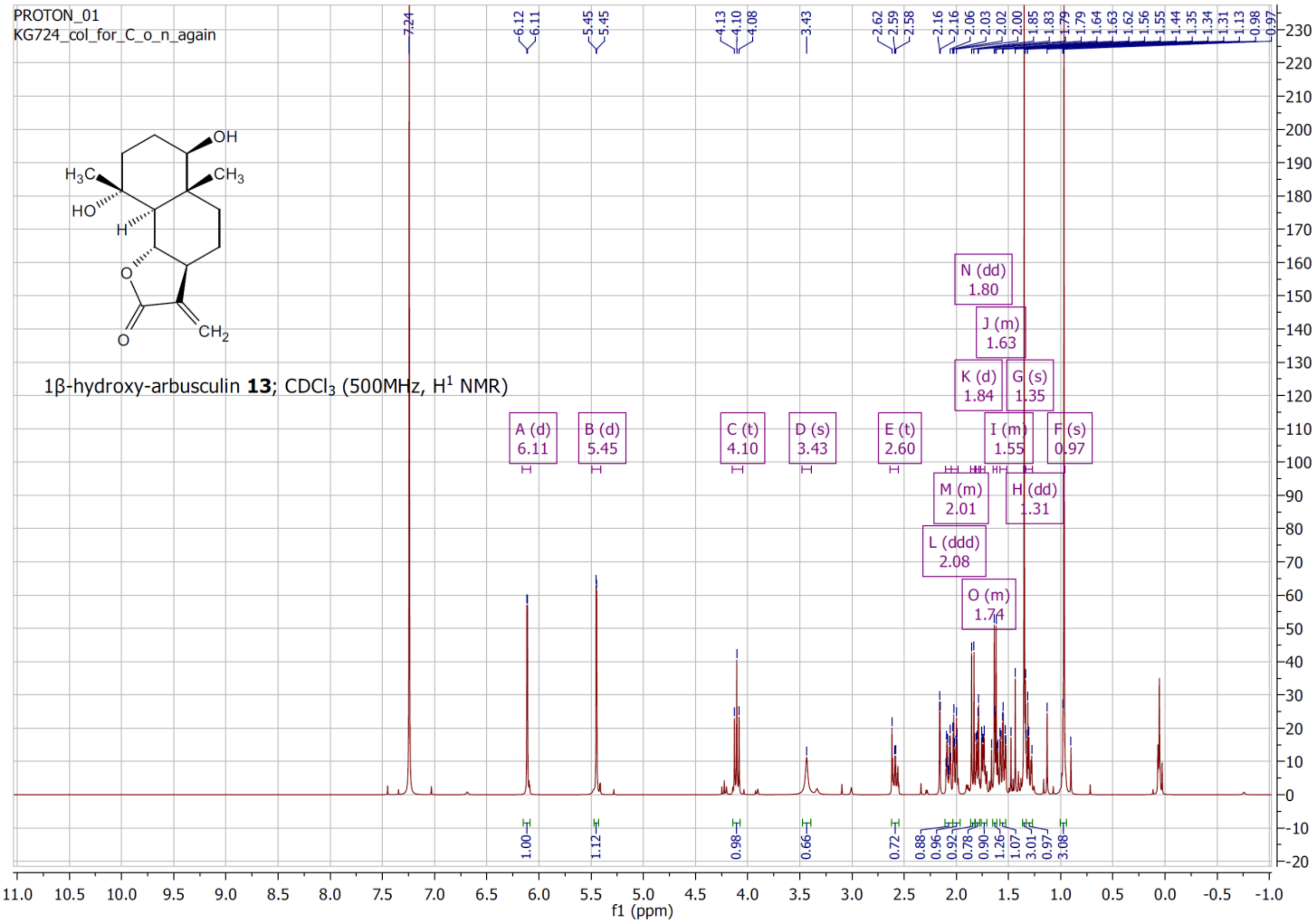
PROTON_01
KG724_col_for_C_o_n_again
OH
H3C
CH3
HO
H
O
O
CH2
1β-hydroxy-arbusculin 13; CDCl3 (500MHz, H1 NMR)
7.24
6.12
6.11
5.45
5.45
4.13
4.10
4.08
3.43
2.62
2.59
2.58
A (d)
6.11
B (d)
5.45
C (t)
4.10
D (s)
3.43
E (t)
2.60
F (s)
0.97
G (s)
1.35
H (dd)
1.31
I (m)
1.55
J (m)
1.63
K (d)
1.84
L (ddd)
2.08
M (m)
2.01
N (dd)
1.80
O (m)
1.74
1.00
1.12
0.98
0.66
0.72
f1 (ppm)

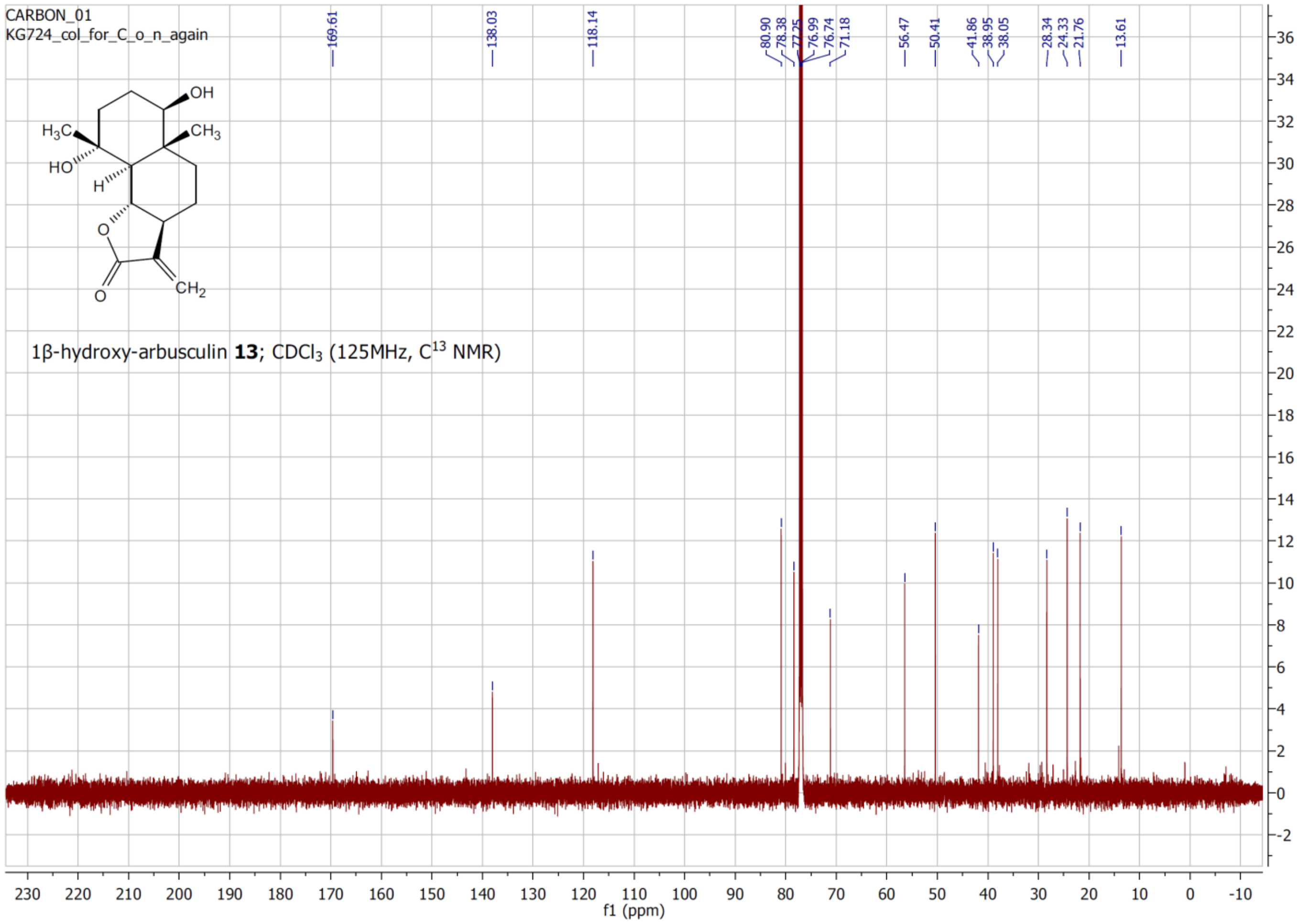
CARBON_01
KG724_col_for_C_o_n_again
OH
H3C
CH3
HO
H
O
O
CH2
1β-hydroxy-arbusculin 13; CDCl3 (125MHz, C13 NMR)
169.61
138.03
118.14
80.90
78.38
77.25
76.99
76.74
71.18
56.47
50.41
41.86
38.95
38.05
28.34
24.33
21.76
13.61
f1 (ppm)

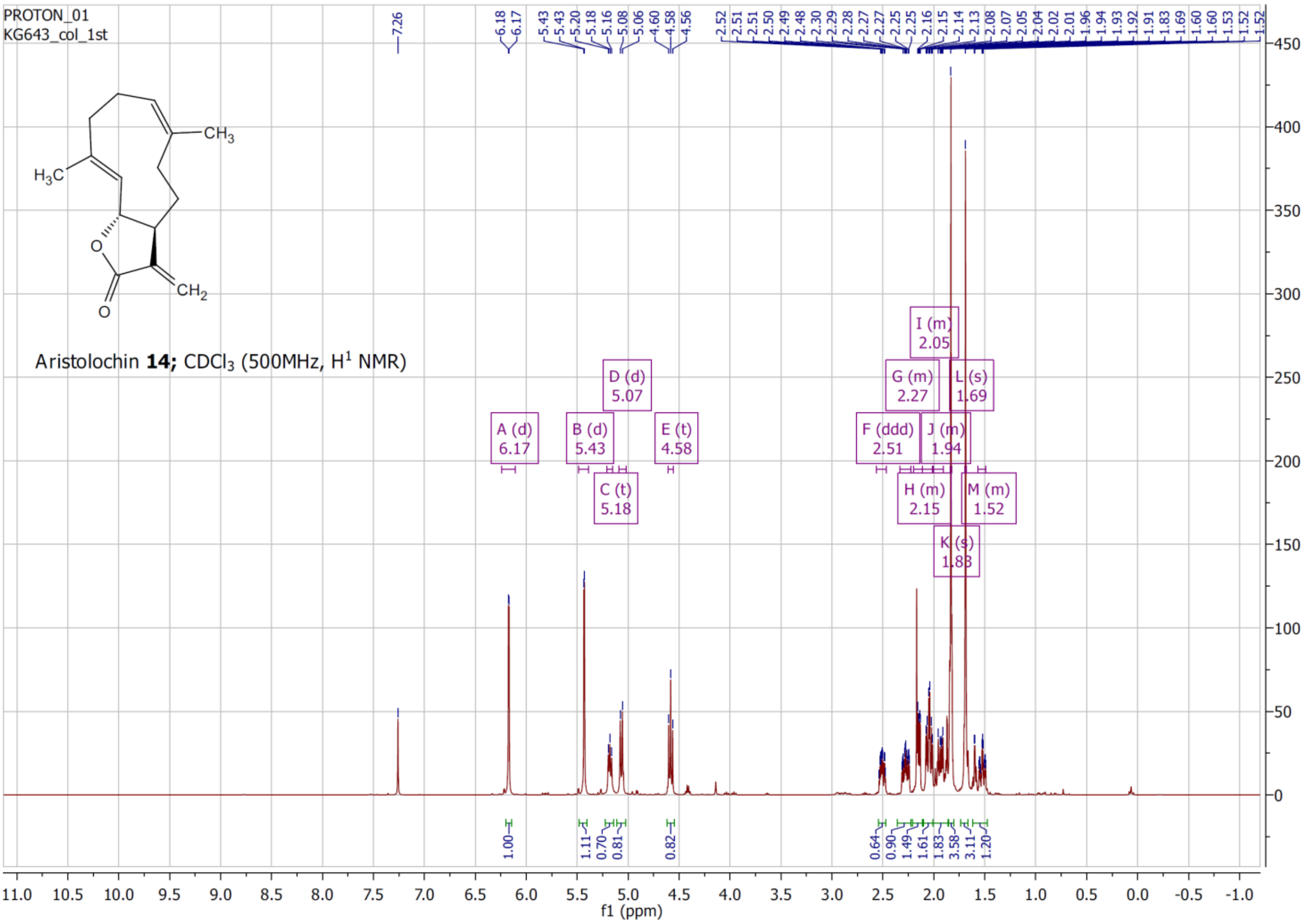
PROTON_01
KG643_col_1st
Aristolochin 14; CDCl3 (500MHz, H1 NMR)
f1 (ppm)

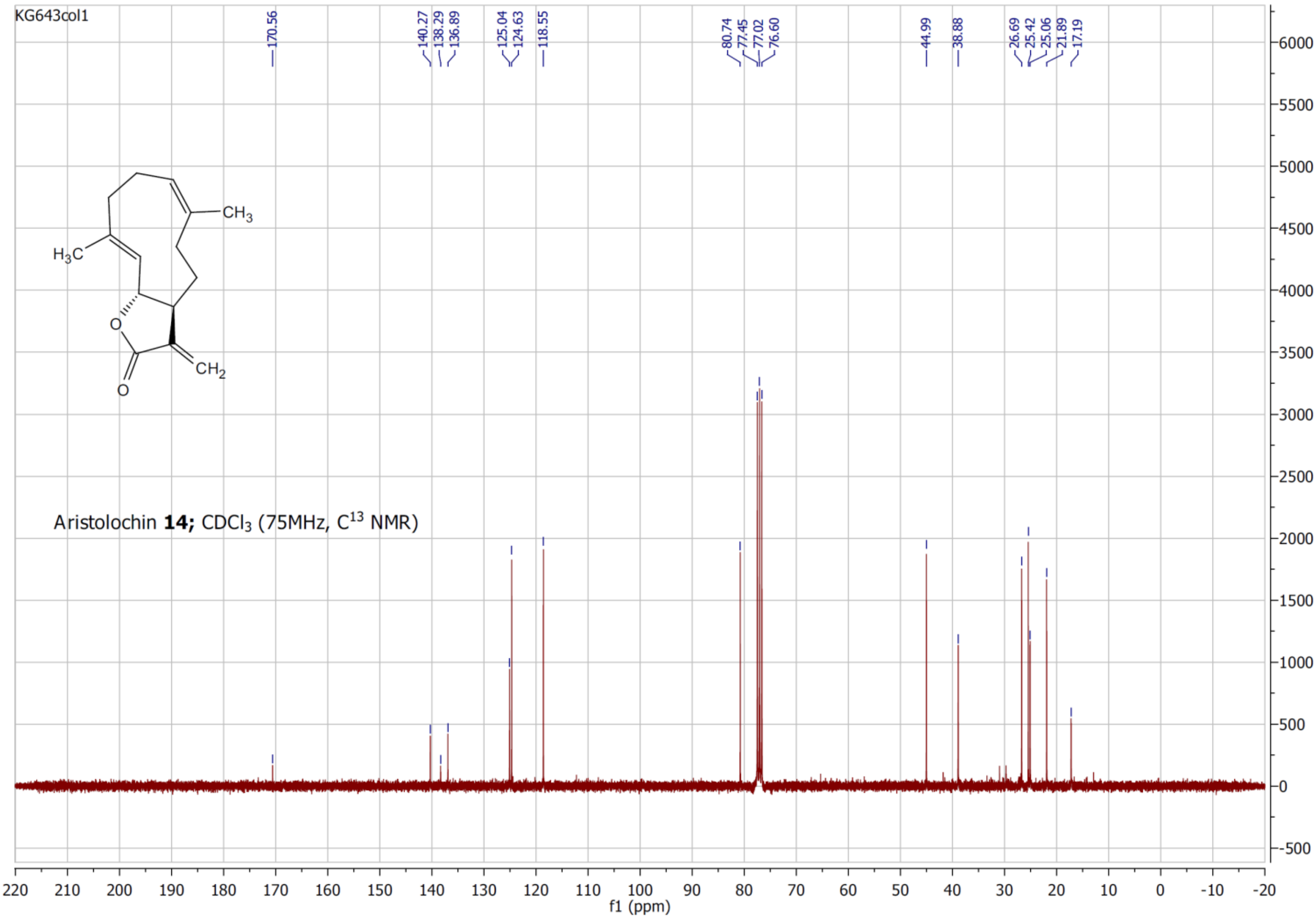
KG643col1
Aristolochin 14; CDCl3 (75MHz, C13 NMR)
170.56
140.27
138.29
136.89
125.04
124.63
118.55
80.74
77.45
77.02
76.60
44.99
38.88
26.69
25.42
25.06
21.89
17.19
f1 (ppm)

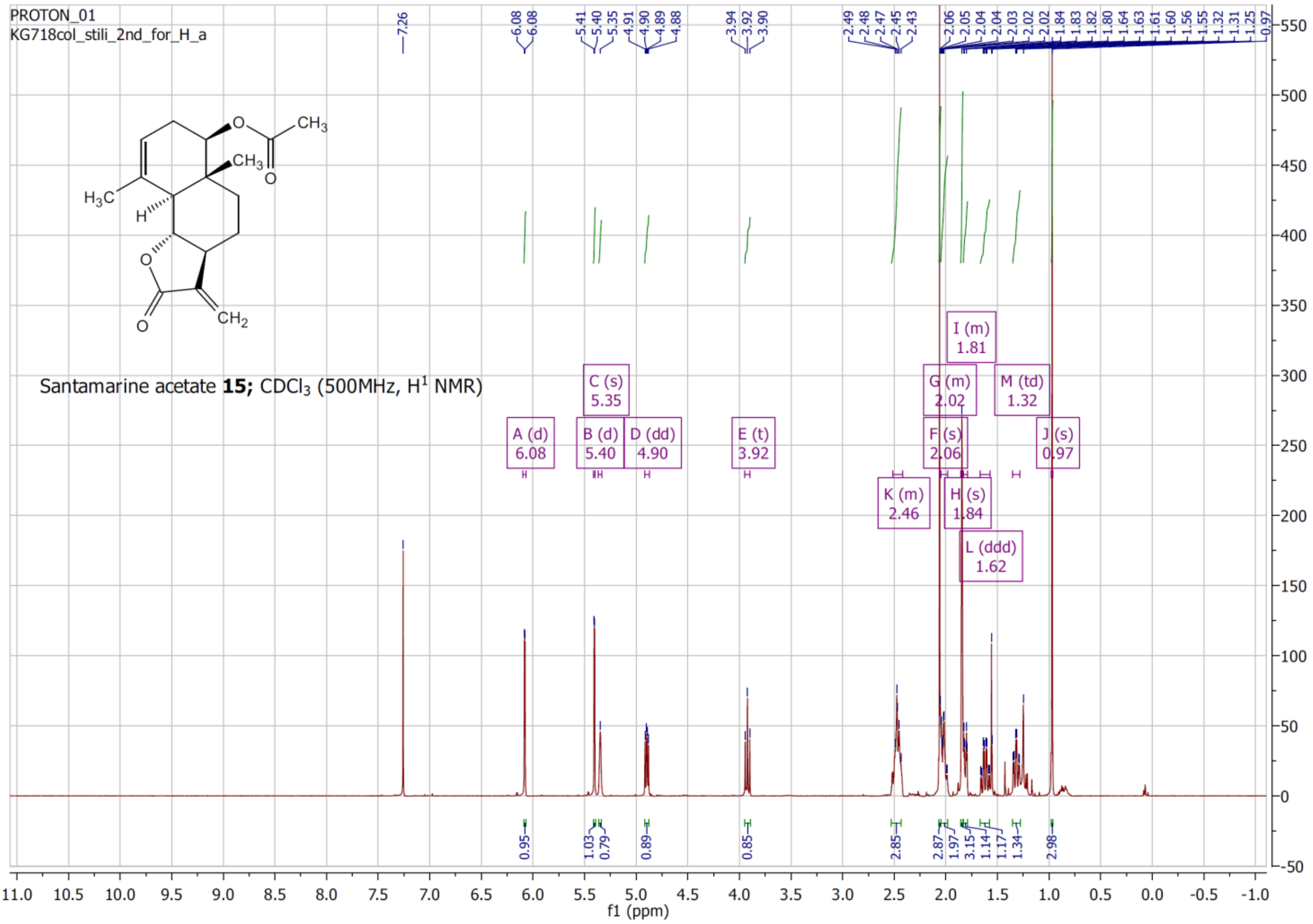

PROTON_01
KG718col_stili_2nd_for_H_a
Santamarine acetate 15; CDCl3 (500MHz, H1 NMR)
A (d) 6.08
B (d) 5.40
C (s) 5.35
D (dd) 4.90
E (t) 3.92
F (s) 2.06
G (m) 2.02
H (s) 1.84
I (m) 1.81
J (s) 0.97
K (m) 2.46
L (ddd) 1.62
M (td) 1.32
f1 (ppm)

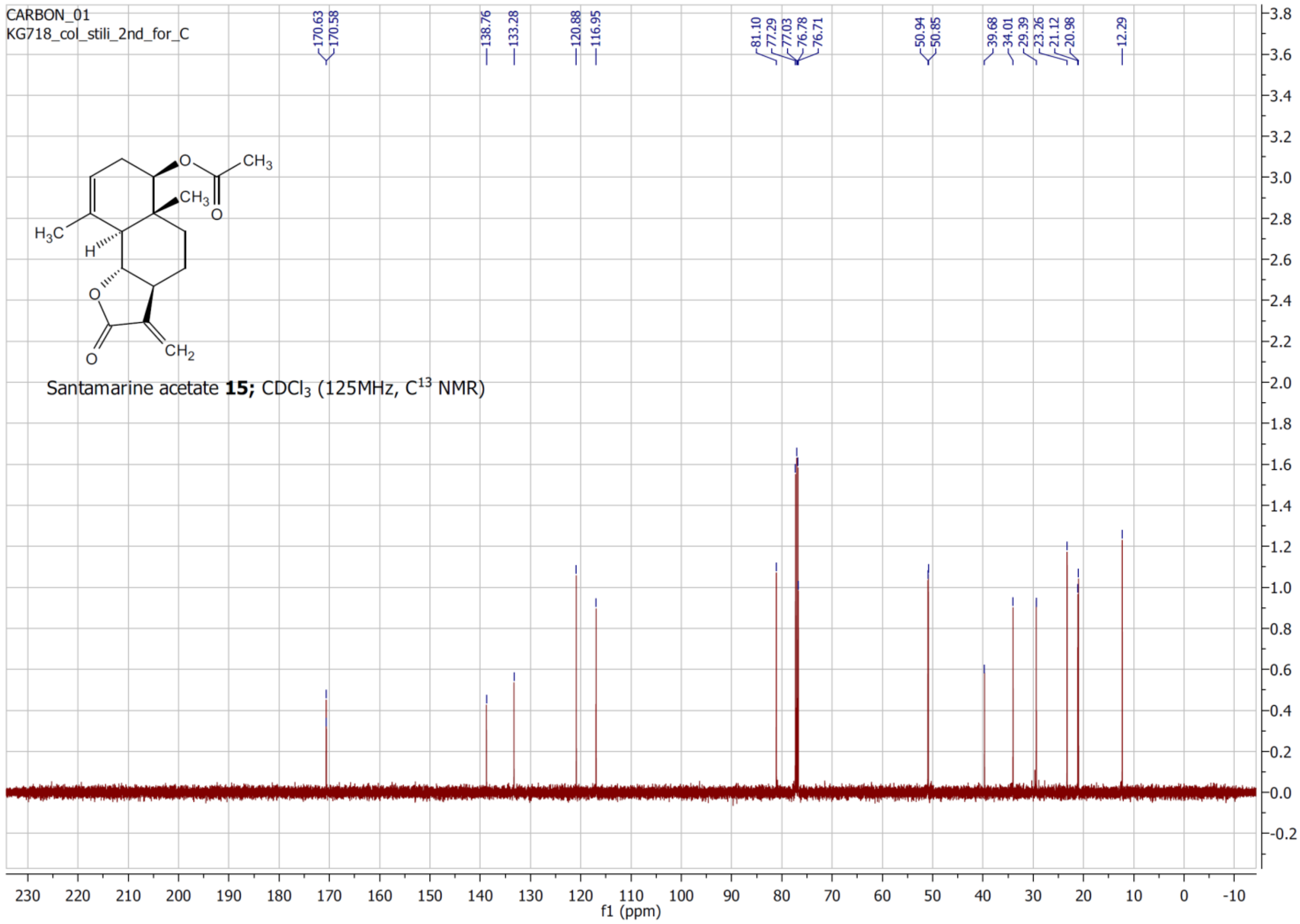

CARBON_01
KG718_col_stili_2nd_for_C
170.63
170.58
138.76
133.28
120.88
116.95
81.10
77.29
77.03
76.78
76.71
50.94
50.85
39.68
34.01
29.39
23.26
21.12
20.98
12.29
Santamarine acetate 15; CDCl3 (125MHz, C13 NMR)
f1 (ppm)

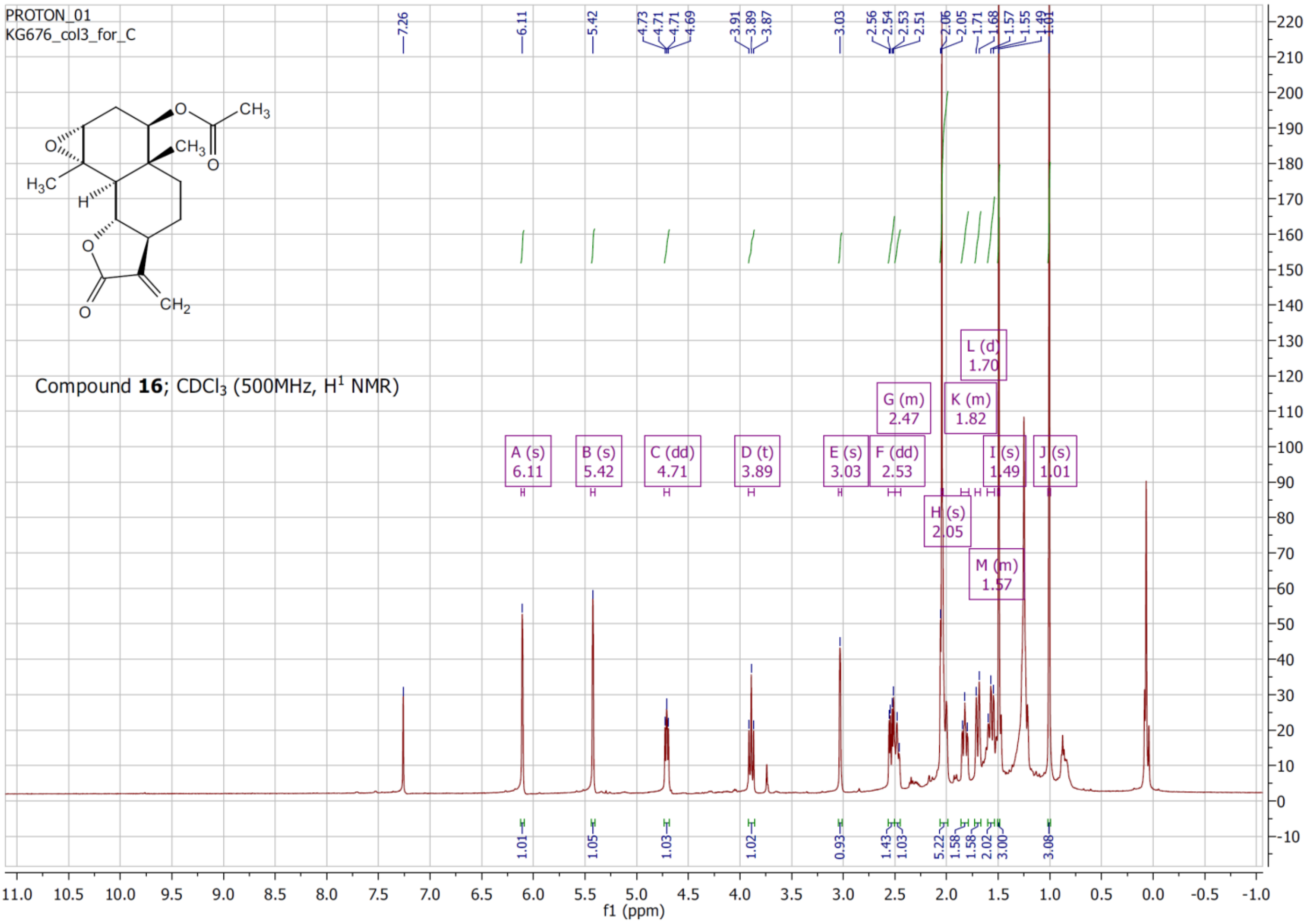
PROTON_01
KG676_col3_for_C
Compound 16; CDCl3 (500MHz, H1 NMR)
7.26
6.11
5.42
4.73
4.71
4.71
4.69
3.91
3.89
3.87
3.03
2.56
2.54
2.53
2.51
2.06
2.05
1.71
1.68
1.57
1.55
1.49
1.01
A (s)
6.11
B (s)
5.42
C (dd)
4.71
D (t)
3.89
E (s)
3.03
F (dd)
2.53
G (m)
2.47
H (s)
2.05
I (s)
1.49
J (s)
1.01
K (m)
1.82
L (d)
1.70
M (m)
1.57
1.01
1.05
1.03
1.02
0.93
1.43
1.03
5.22
1.58
1.58
2.02
3.00
3.08
f1 (ppm)

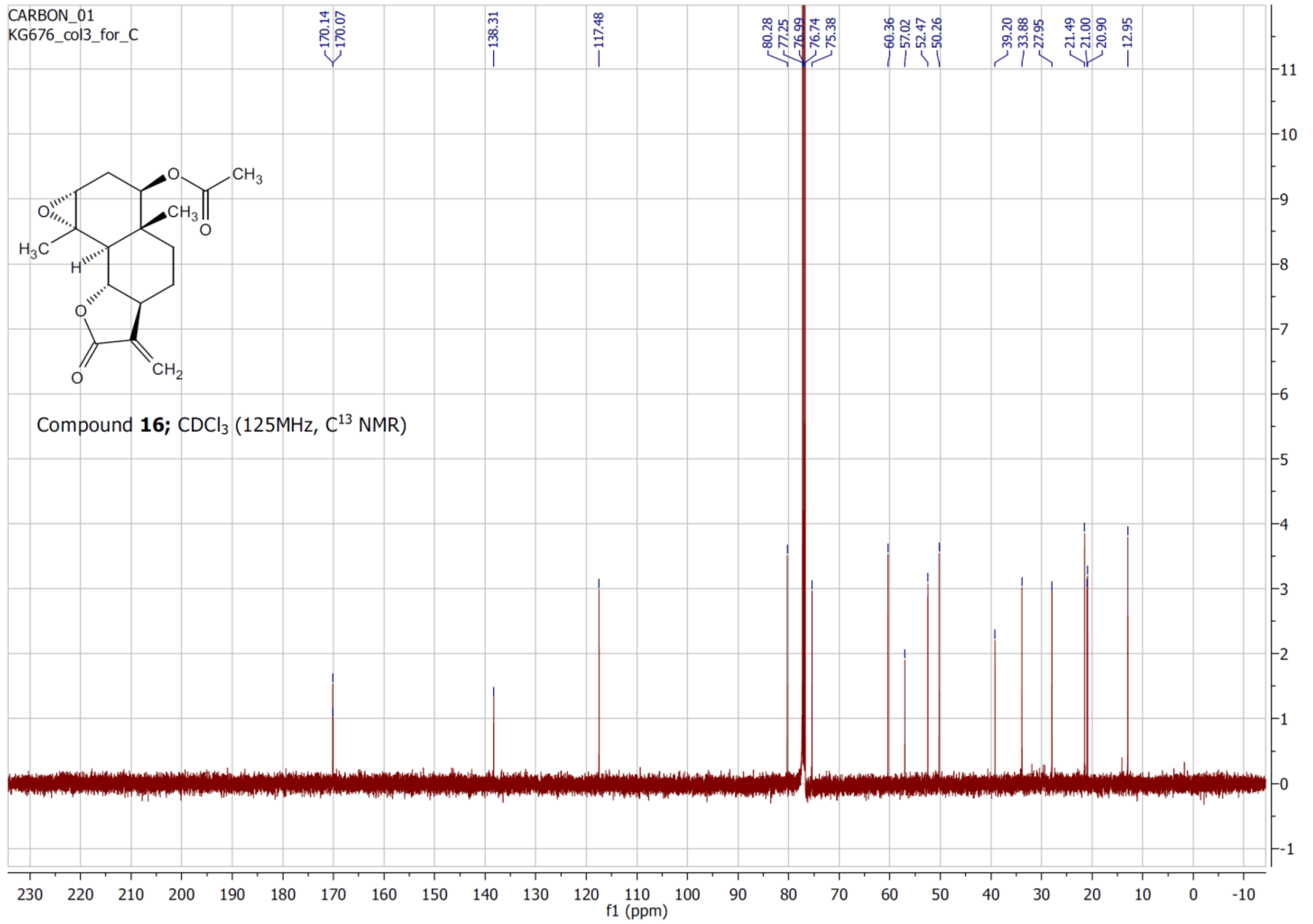
CARBON_01
KG676_col3_for_C
Compound 16; CDCl3 (125MHz, C13 NMR)
170.14
170.07
138.31
117.48
80.28
77.25
76.99
76.74
75.38
60.36
57.02
52.47
50.26
39.20
33.88
27.95
21.49
21.00
20.90
12.95
f1 (ppm)

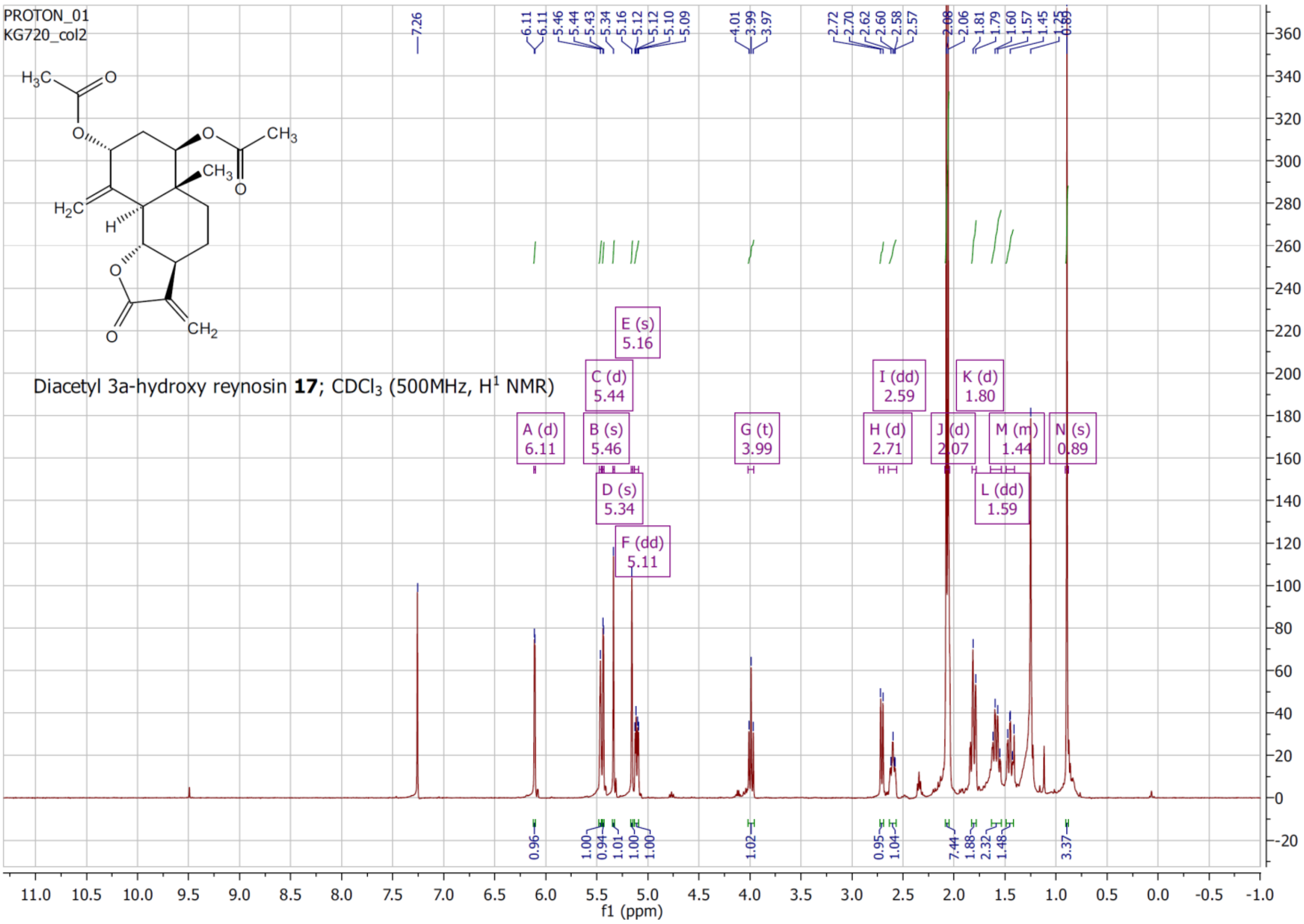
PROTON_01
KG720_col2
Diacetyl 3a-hydroxy reynosin **17**; $CDCl_3$ (500MHz, H[1] NMR)
A (d) 6.11
B (s) 5.46
C (d) 5.44
D (s) 5.34
E (s) 5.16
F (dd) 5.11
G (t) 3.99
H (d) 2.71
I (dd) 2.59
J (d) 2.07
K (d) 1.80
L (dd) 1.59
M (m) 1.44
N (s) 0.89
f1 (ppm)

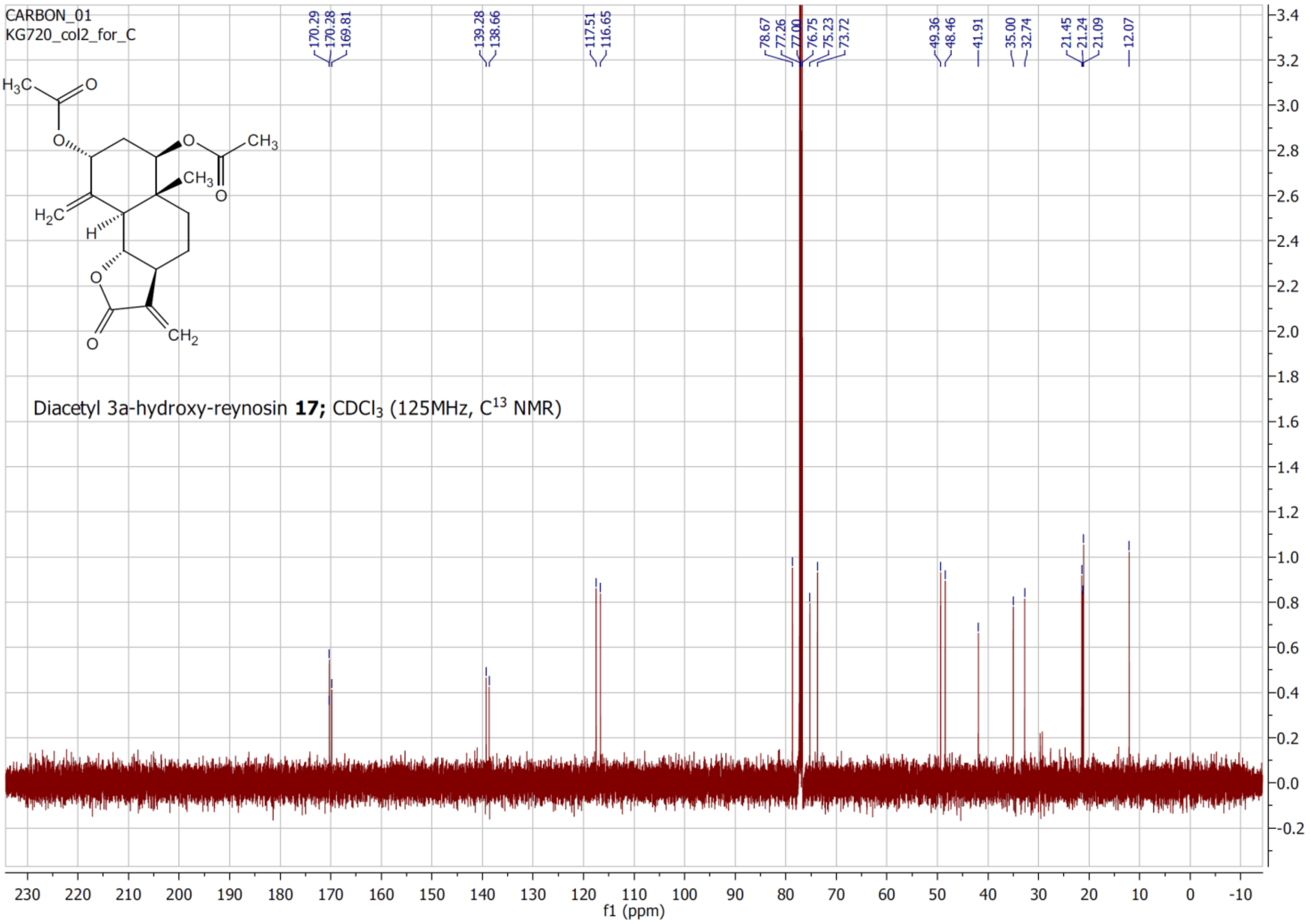
CARBON_01
KG720_col2_for_C
170.29
170.28
169.81
139.28
138.66
117.51
116.65
78.67
77.26
77.00
76.75
75.23
73.72
49.36
48.46
41.91
35.00
32.74
21.45
21.24
21.09
12.07
Diacetyl 3a-hydroxy-reynosin **17;** $CDCl_3$ (125MHz, C[13] NMR)
f1 (ppm)

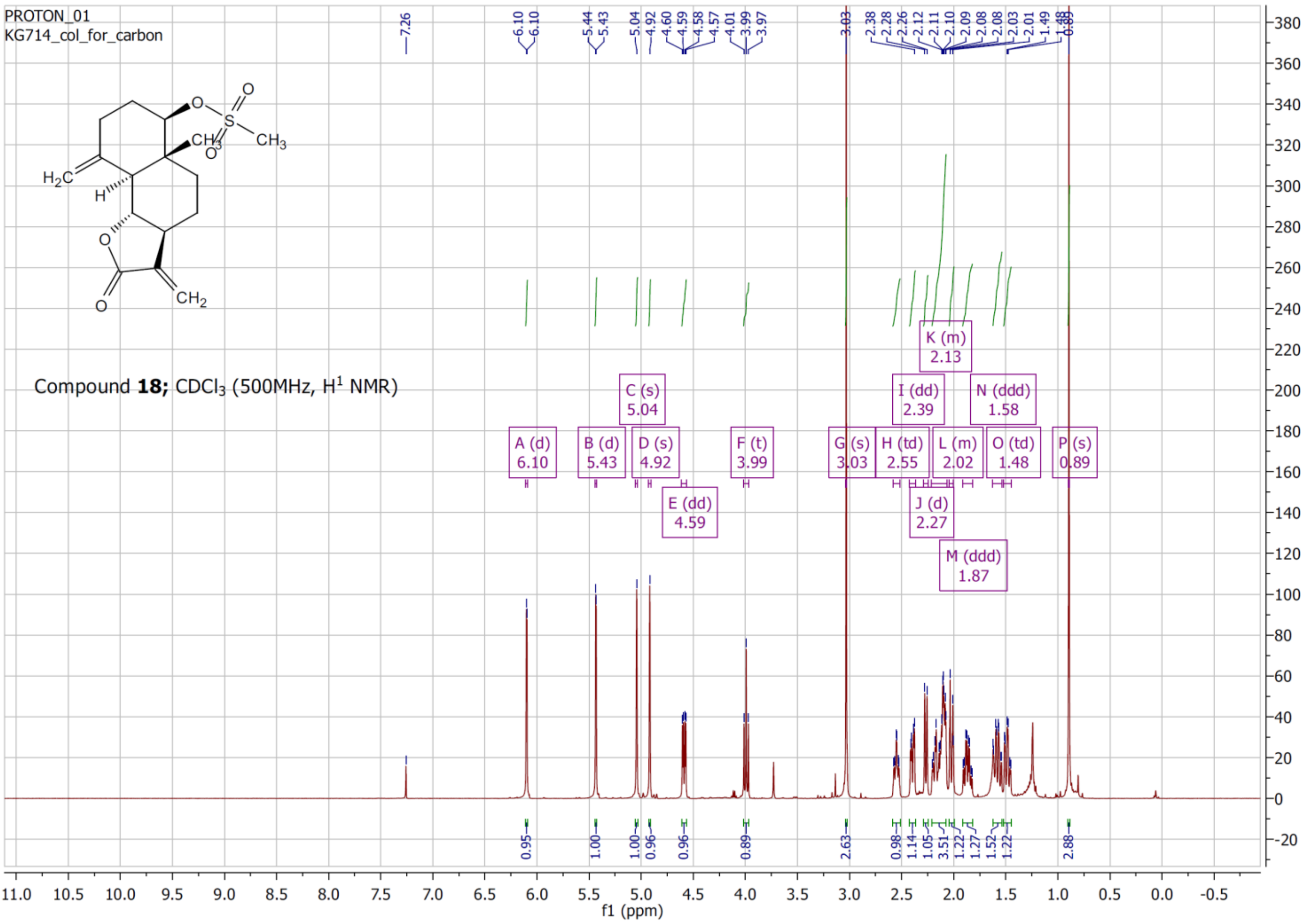
PROTON_01
KG714_col_for_carbon
7.26
6.10 6.10
5.44 5.43
5.04 4.92
4.60 4.59 4.58 4.57
4.01 3.99 3.97
3.03
2.38 2.28 2.26 2.12 2.11 2.10 2.09 2.08 2.08 2.03 2.01 1.49 1.48
0.89
Compound 18; CDCl3 (500MHz, H1 NMR)
A (d) 6.10
B (d) 5.43
C (s) 5.04
D (s) 4.92
E (dd) 4.59
F (t) 3.99
G (s) 3.03
H (td) 2.55
I (dd) 2.39
J (d) 2.27
K (m) 2.13
L (m) 2.02
M (ddd) 1.87
N (ddd) 1.58
O (td) 1.48
P (s) 0.89
0.95 1.00 1.00 0.96 0.96 0.89 2.63 0.98 1.14 1.05 3.51 1.22 1.27 1.52 1.22 2.88
f1 (ppm)

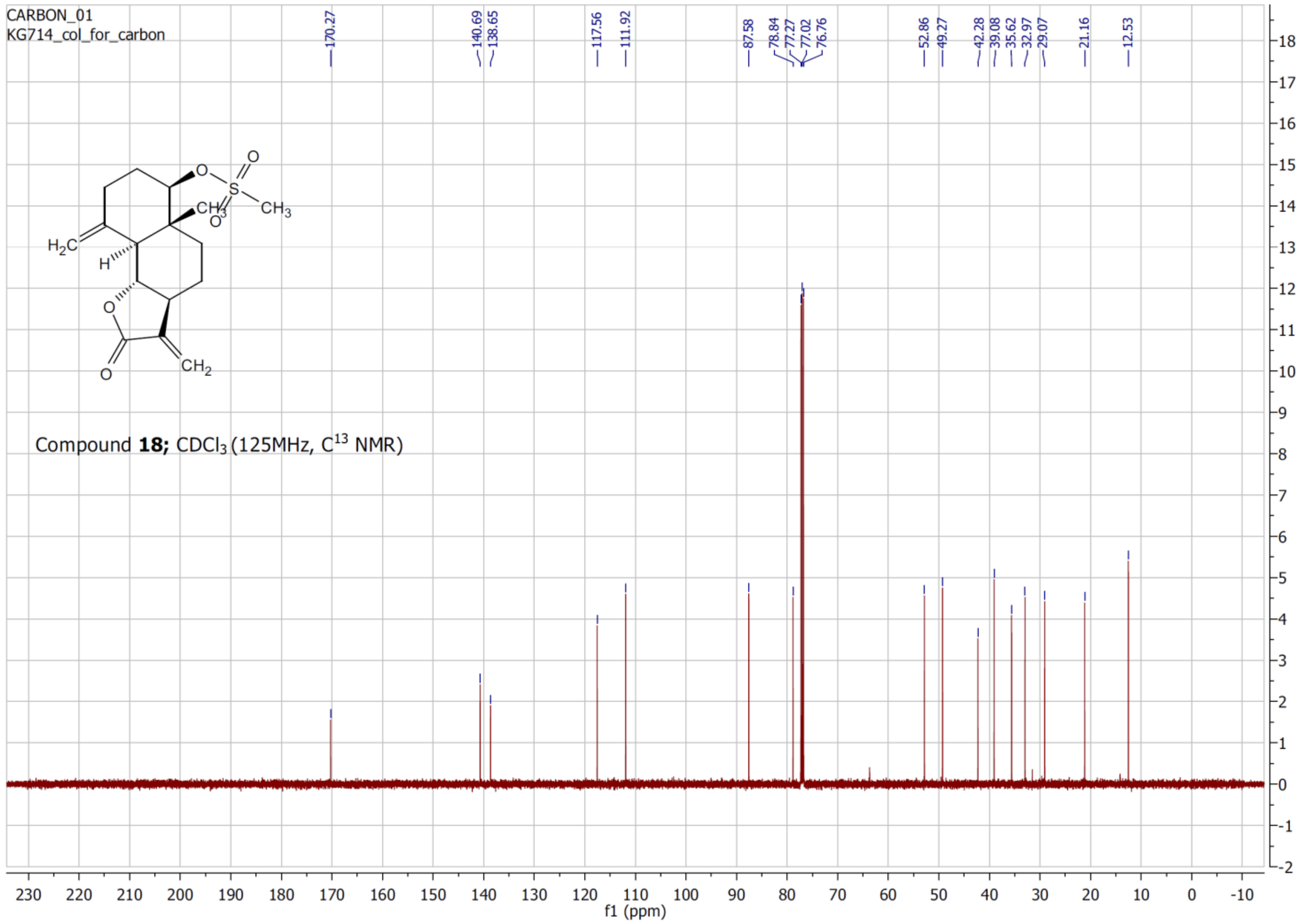
CARBON_01
KG714_col_for_carbon
170.27
140.69 138.65
117.56 111.92
87.58
78.84 77.27 77.02 76.76
52.86 49.27
42.28 39.08 35.62 32.97 29.07
21.16
12.53
Compound 18; CDCl3 (125MHz, C13 NMR)
f1 (ppm)

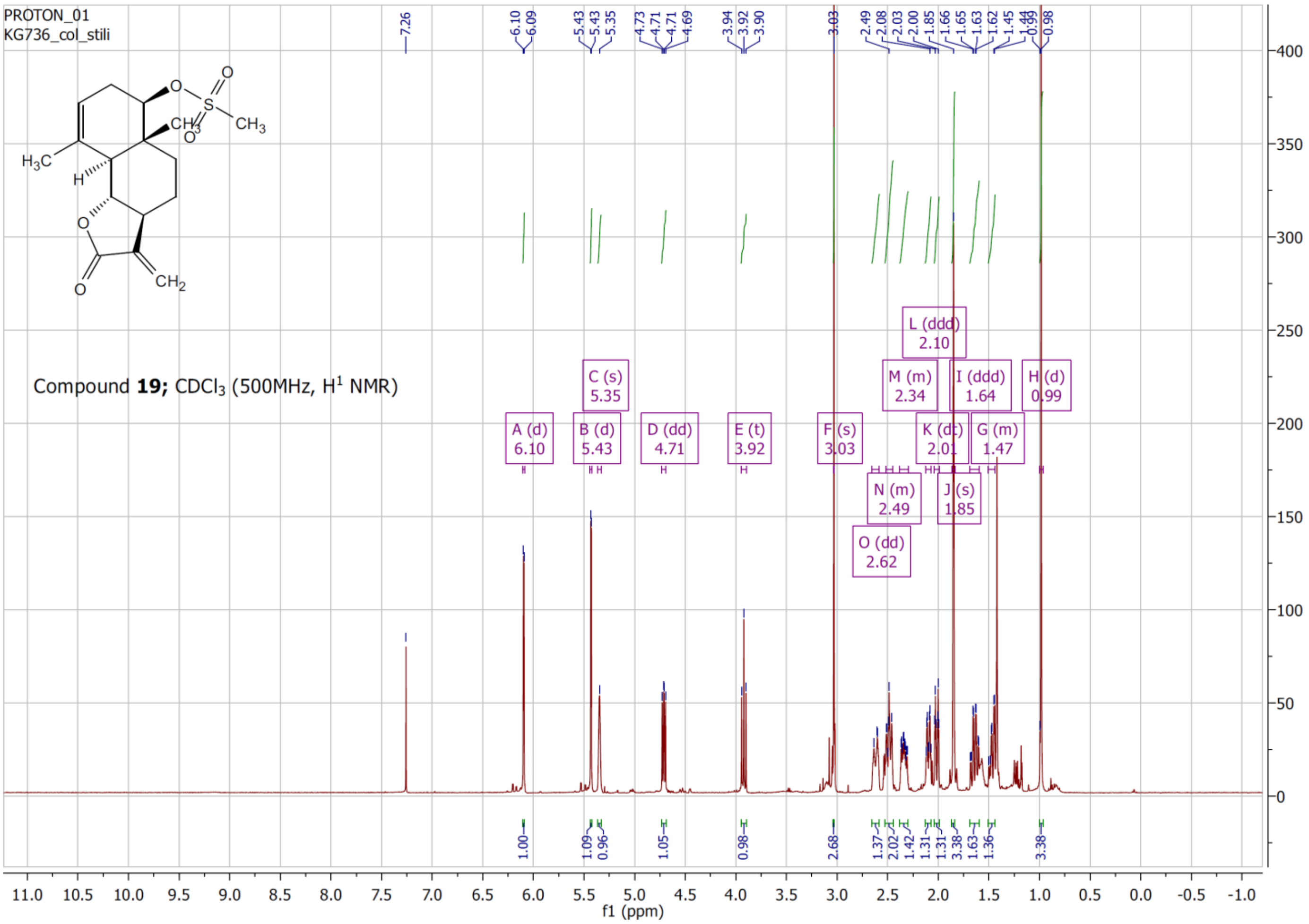
PROTON_01
KG736_col_stili
Compound 19; CDCl3 (500MHz, H1 NMR)
7.26
6.10
6.09
5.43
5.43
5.35
4.73
4.71
4.71
4.69
3.94
3.92
3.90
3.03
2.49
2.08
2.03
2.00
1.85
1.66
1.65
1.63
1.62
1.45
1.44
0.99
0.98
A (d) 6.10
B (d) 5.43
C (s) 5.35
D (dd) 4.71
E (t) 3.92
F (s) 3.03
G (m) 1.47
H (d) 0.99
I (ddd) 1.64
J (s) 1.85
K (dt) 2.01
L (ddd) 2.10
M (m) 2.34
N (m) 2.49
O (dd) 2.62
1.00
1.09
0.96
1.05
0.98
2.68
1.37
2.02
1.42
1.31
1.31
3.38
1.63
1.36
3.38
f1 (ppm)

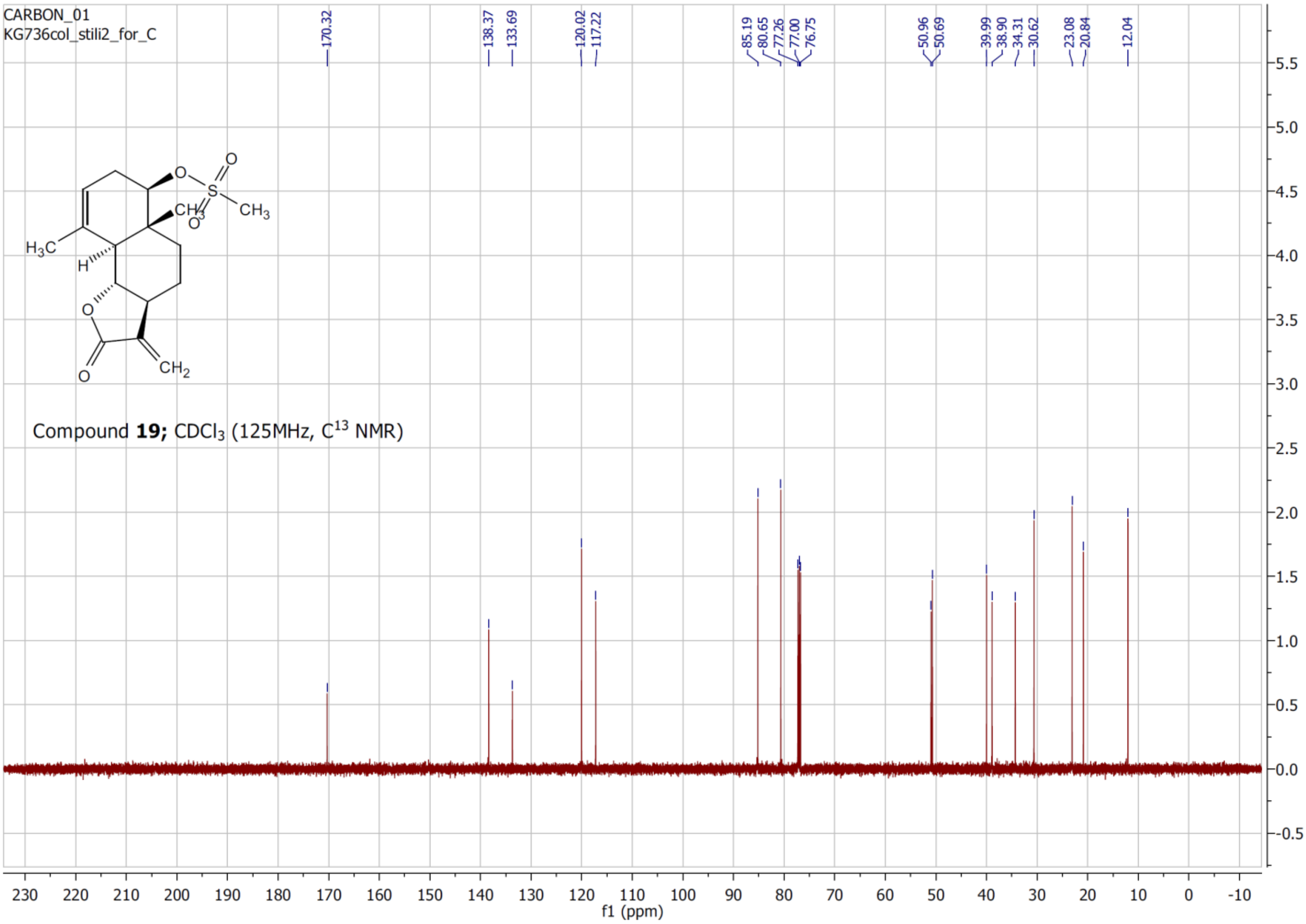
CARBON_01
KG736col_stili2_for_C
Compound 19; CDCl3 (125MHz, C13 NMR)
170.32
138.37
133.69
120.02
117.22
85.19
80.65
77.26
77.00
76.75
50.96
50.69
39.99
38.90
34.31
30.62
23.08
20.84
12.04
f1 (ppm)

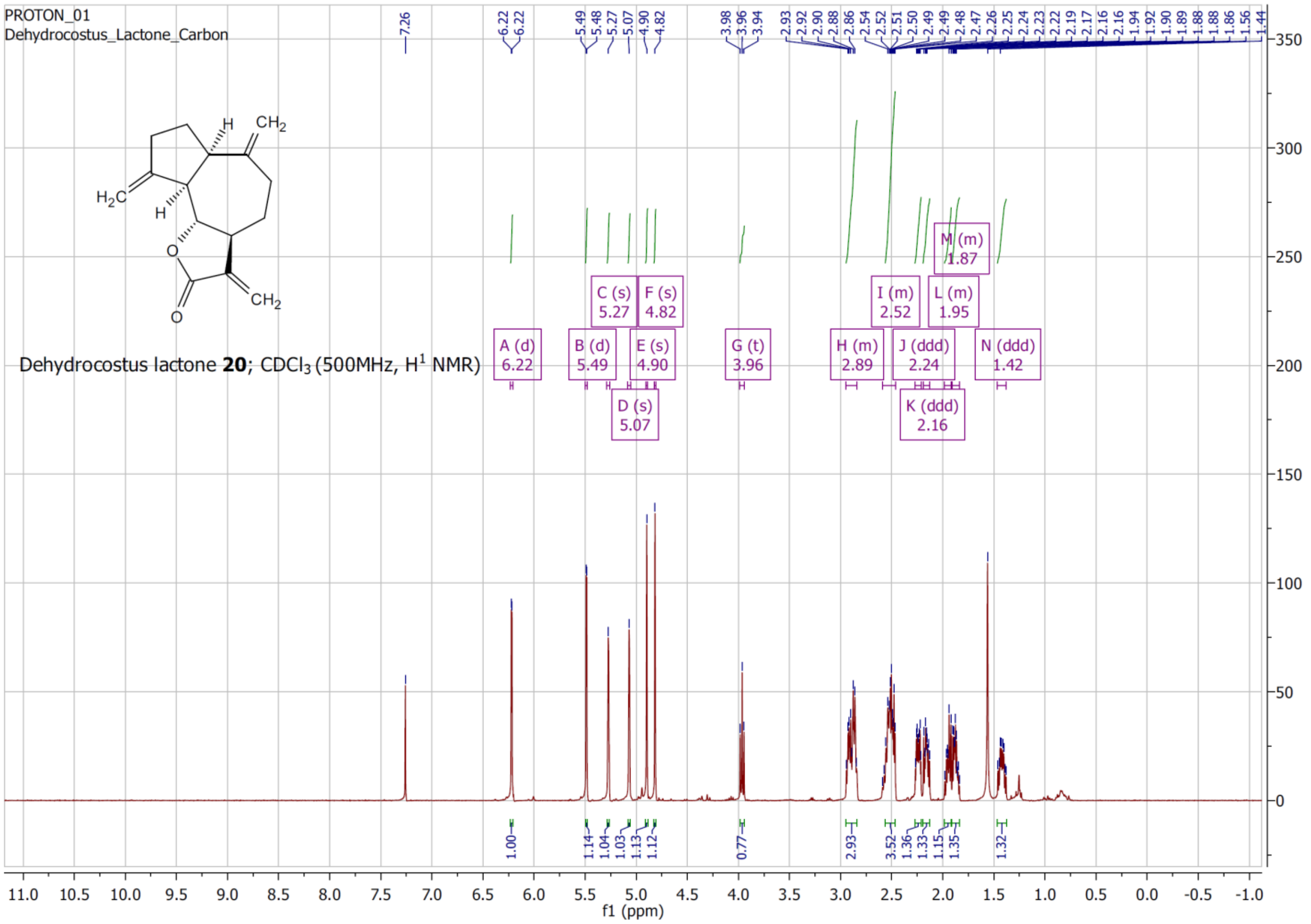
PROTON_01
Dehydrocostus_Lactone_Carbon
7.26
6.22
6.22
5.49
5.48
5.27
5.07
4.90
4.82
3.98
3.96
3.94
2.93
2.92
2.90
2.88
2.86
2.54
2.52
2.51
2.50
2.49
2.49
2.48
2.47
2.26
2.25
2.24
2.23
2.22
2.19
2.17
2.16
2.16
1.94
1.92
1.90
1.89
1.88
1.88
1.86
1.56
1.44
Dehydrocostus lactone 20; CDCl3 (500MHz, H1 NMR)
A (d) 6.22
B (d) 5.49
C (s) 5.27
D (s) 5.07
E (s) 4.90
F (s) 4.82
G (t) 3.96
H (m) 2.89
I (m) 2.52
J (ddd) 2.24
K (ddd) 2.16
L (m) 1.95
M (m) 1.87
N (ddd) 1.42
1.00
1.14
1.04
1.03
1.13
1.12
0.77
2.93
3.52
1.36
1.33
1.15
1.35
1.32
f1 (ppm)

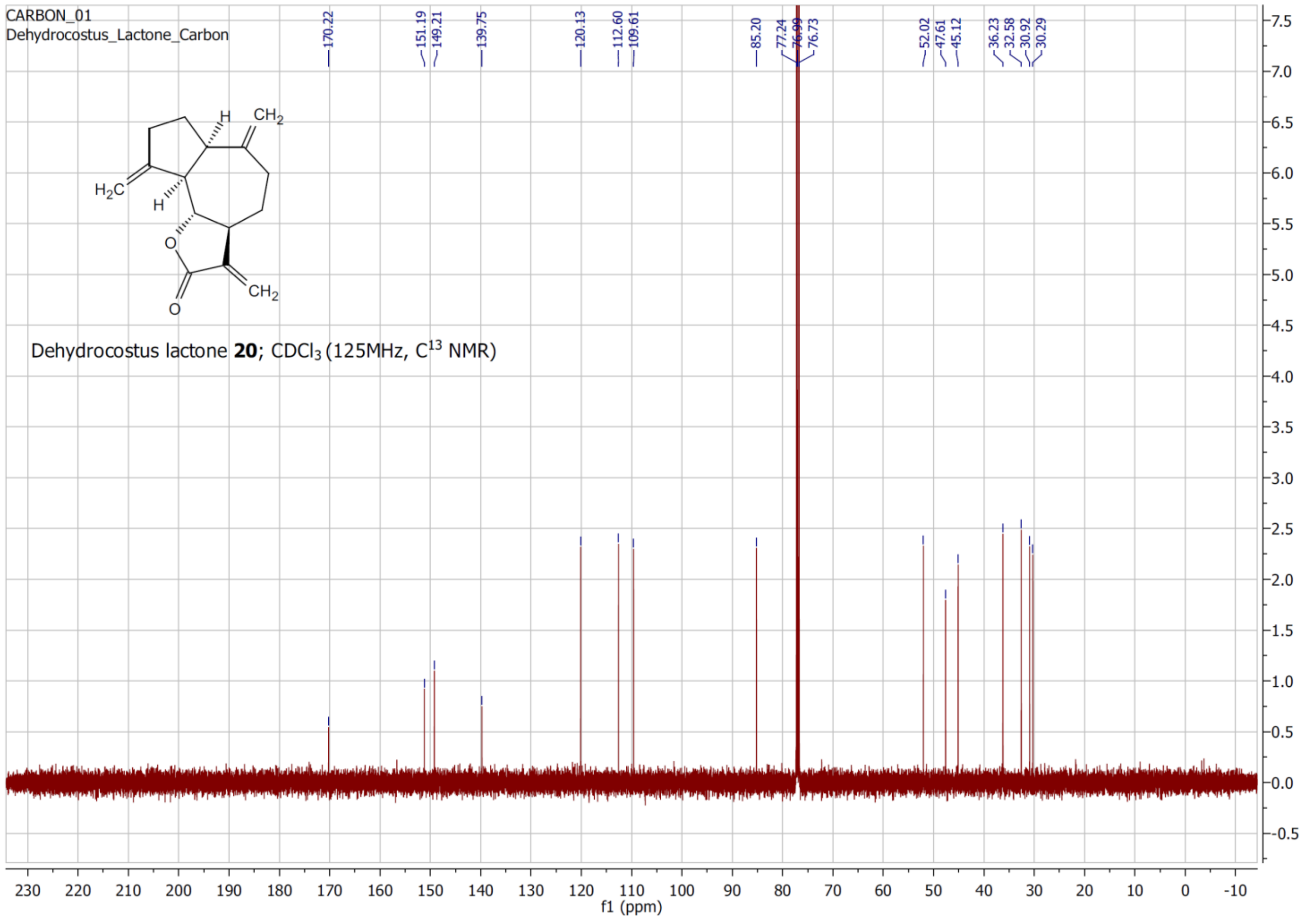
CARBON_01
Dehydrocostus_Lactone_Carbon
170.22
151.19
149.21
139.75
120.13
112.60
109.61
85.20
77.24
76.99
76.73
52.02
47.61
45.12
36.23
32.58
30.92
30.29
Dehydrocostus lactone 20; CDCl3 (125MHz, C13 NMR)
f1 (ppm)

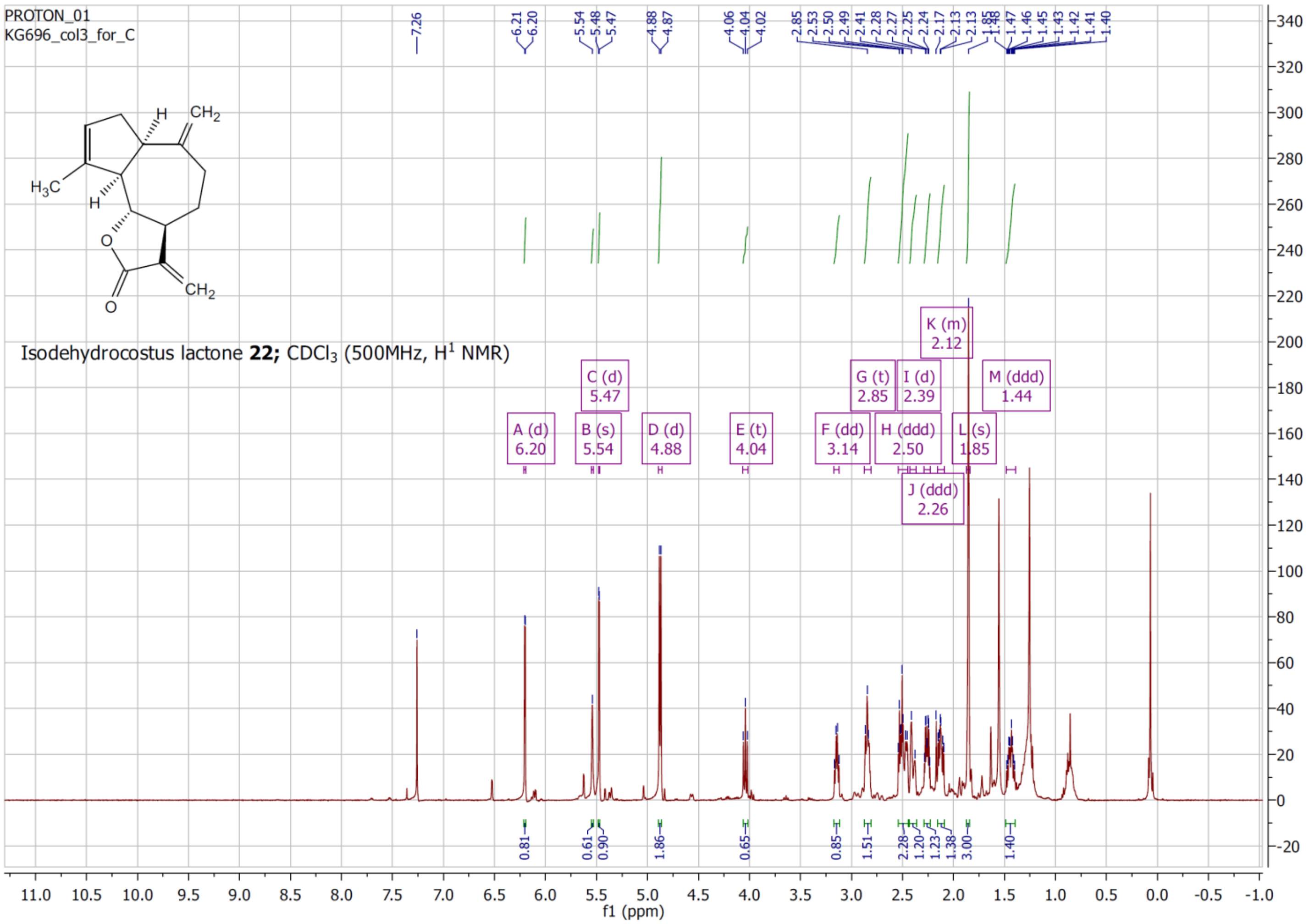
PROTON_01
KG696_col3_for_C
Isodehydrocostus lactone **22**; $CDCl_3$ (500MHz, H^1 NMR)
7.26
6.21
6.20
5.54
5.48
5.47
4.88
4.87
4.06
4.04
4.02
A (d) 6.20
B (s) 5.54
C (d) 5.47
D (d) 4.88
E (t) 4.04
F (dd) 3.14
G (t) 2.85
H (ddd) 2.50
I (d) 2.39
J (ddd) 2.26
K (m) 2.12
L (s) 1.85
M (ddd) 1.44
0.81
0.61
0.90
1.86
0.65
0.85
1.51
2.28
1.20
1.23
1.38
3.00
1.40
f1 (ppm)

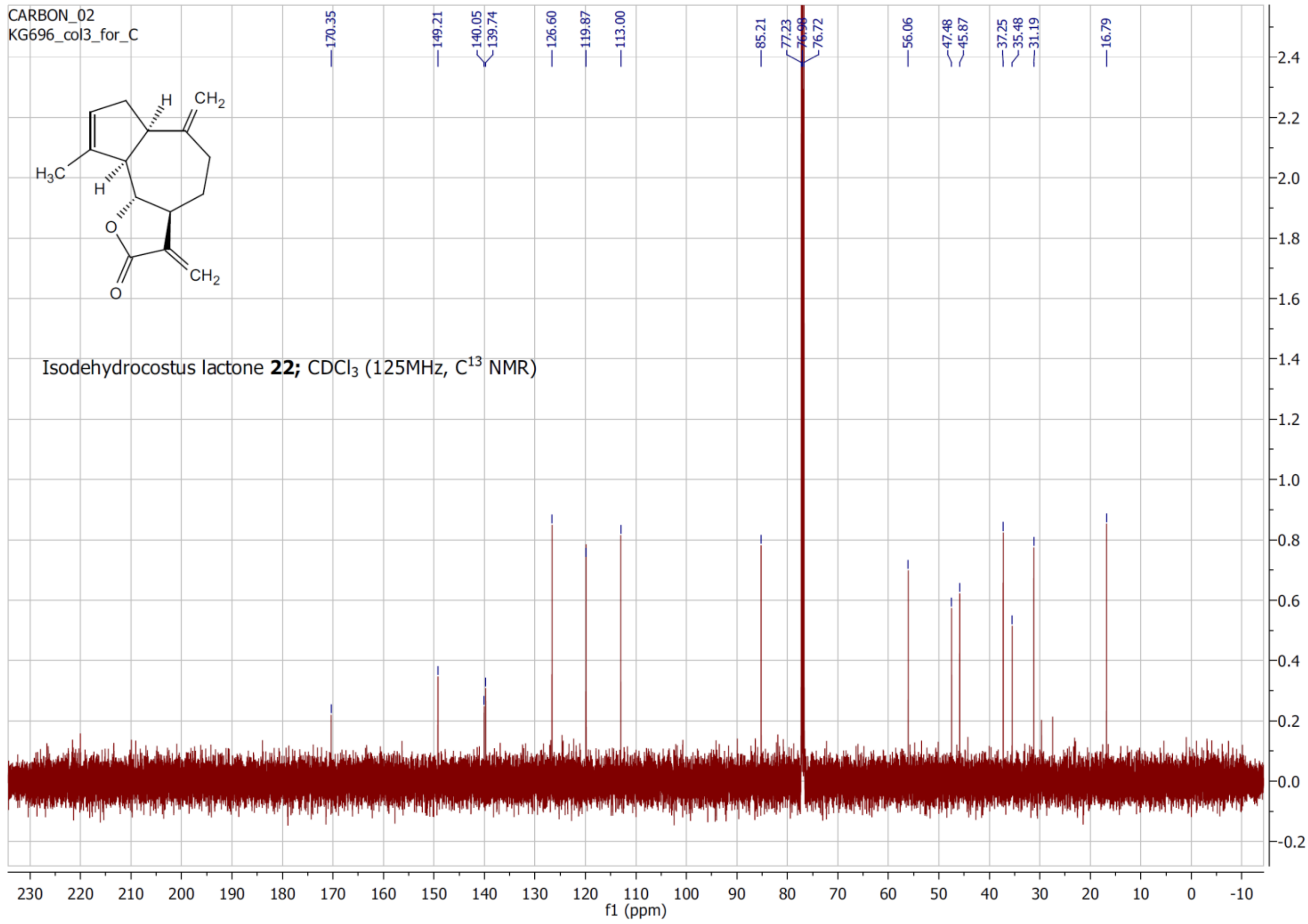
CARBON_02
KG696_col3_for_C
Isodehydrocostus lactone **22**; $CDCl_3$ (125MHz, C^{13} NMR)
170.35
149.21
140.05
139.74
126.60
119.87
113.00
85.21
77.23
76.98
76.72
56.06
47.48
45.87
37.25
35.48
31.19
16.79
f1 (ppm)

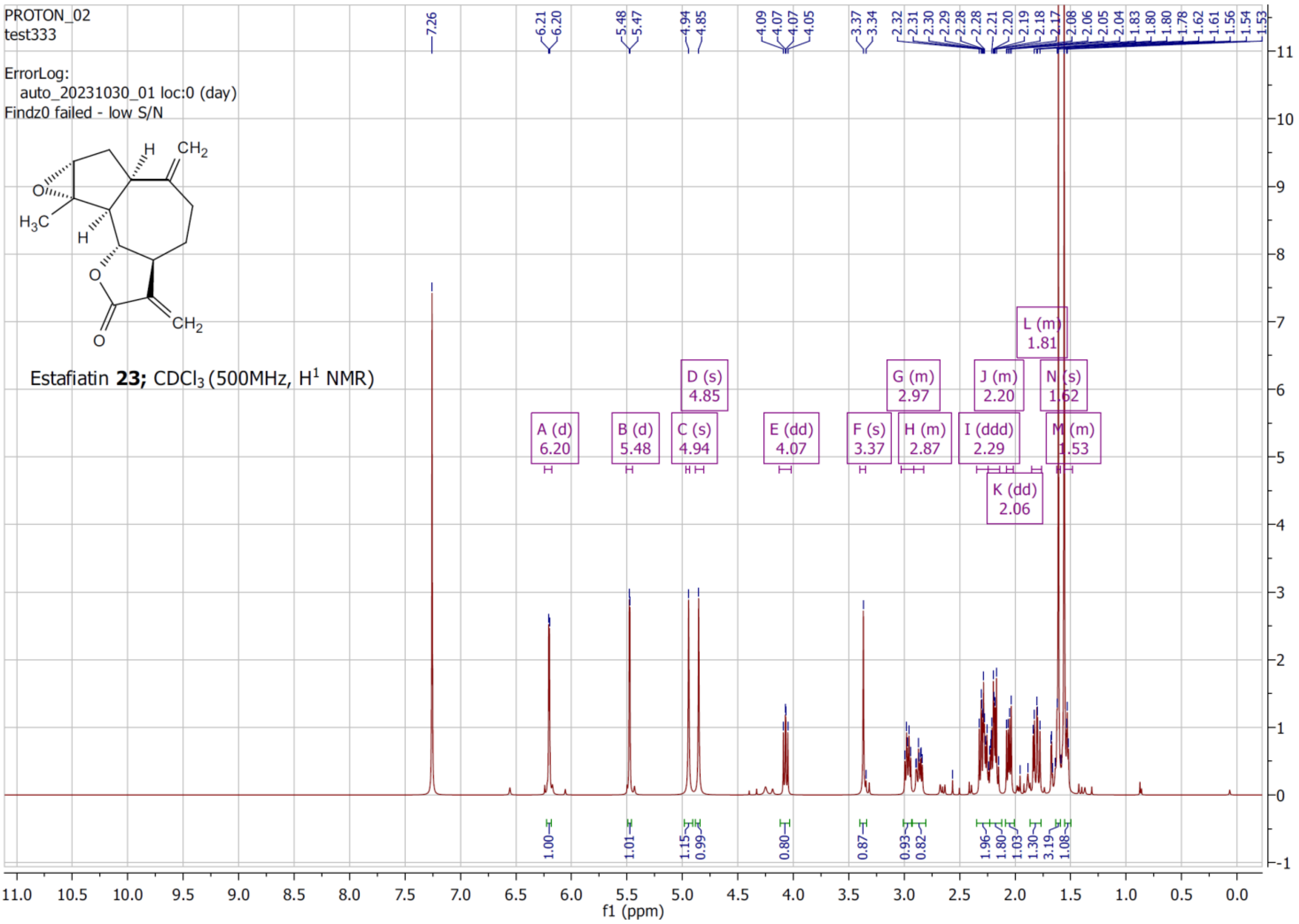

PROTON_02
test333
ErrorLog:
auto_20231030_01 loc:0 (day)
Findz0 failed - low S/N
Estafiatin 23; CDCl3 (500MHz, H1 NMR)

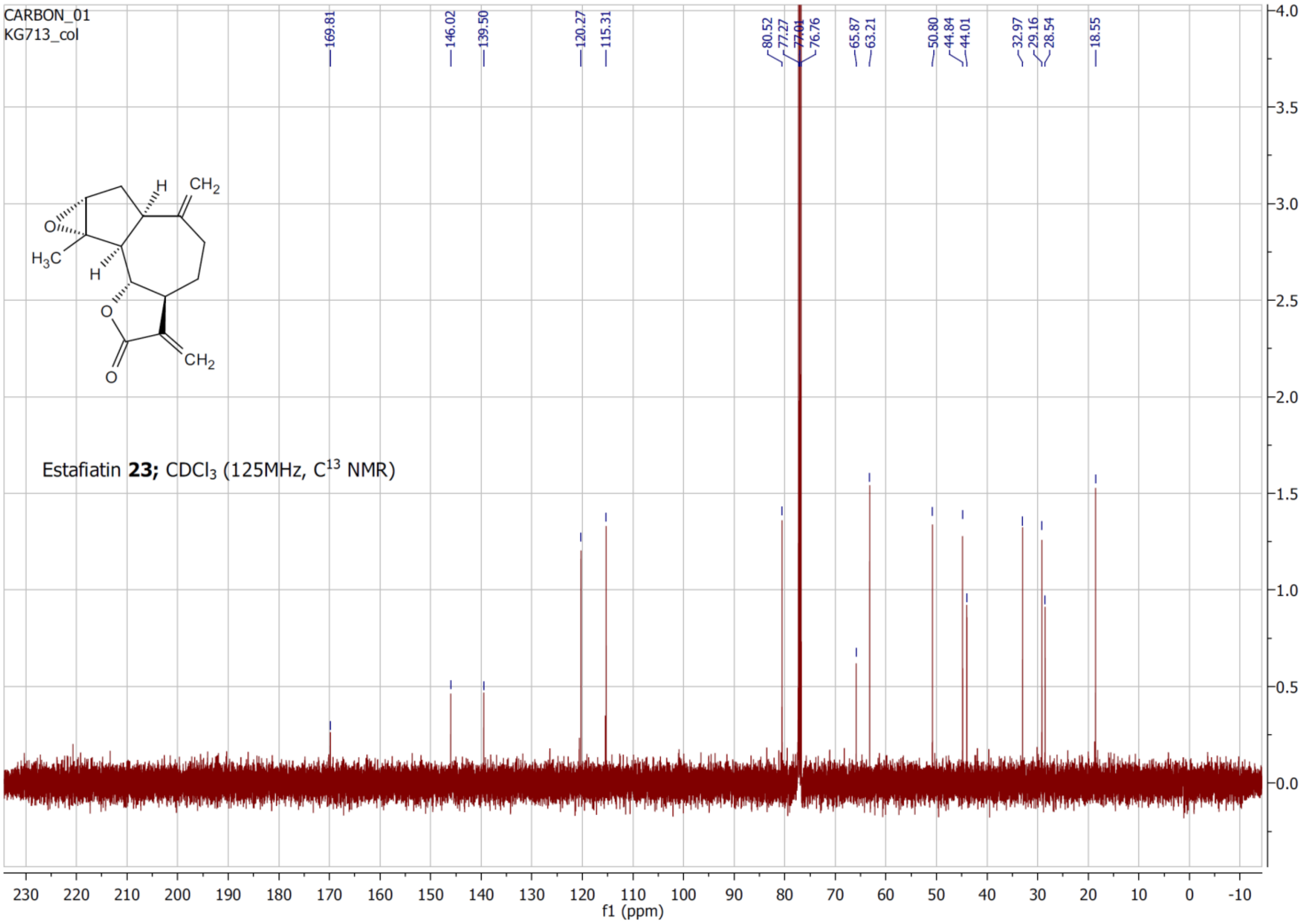

CARBON_01
KG713_col
Estafiatin 23; CDCl3 (125MHz, C13 NMR)

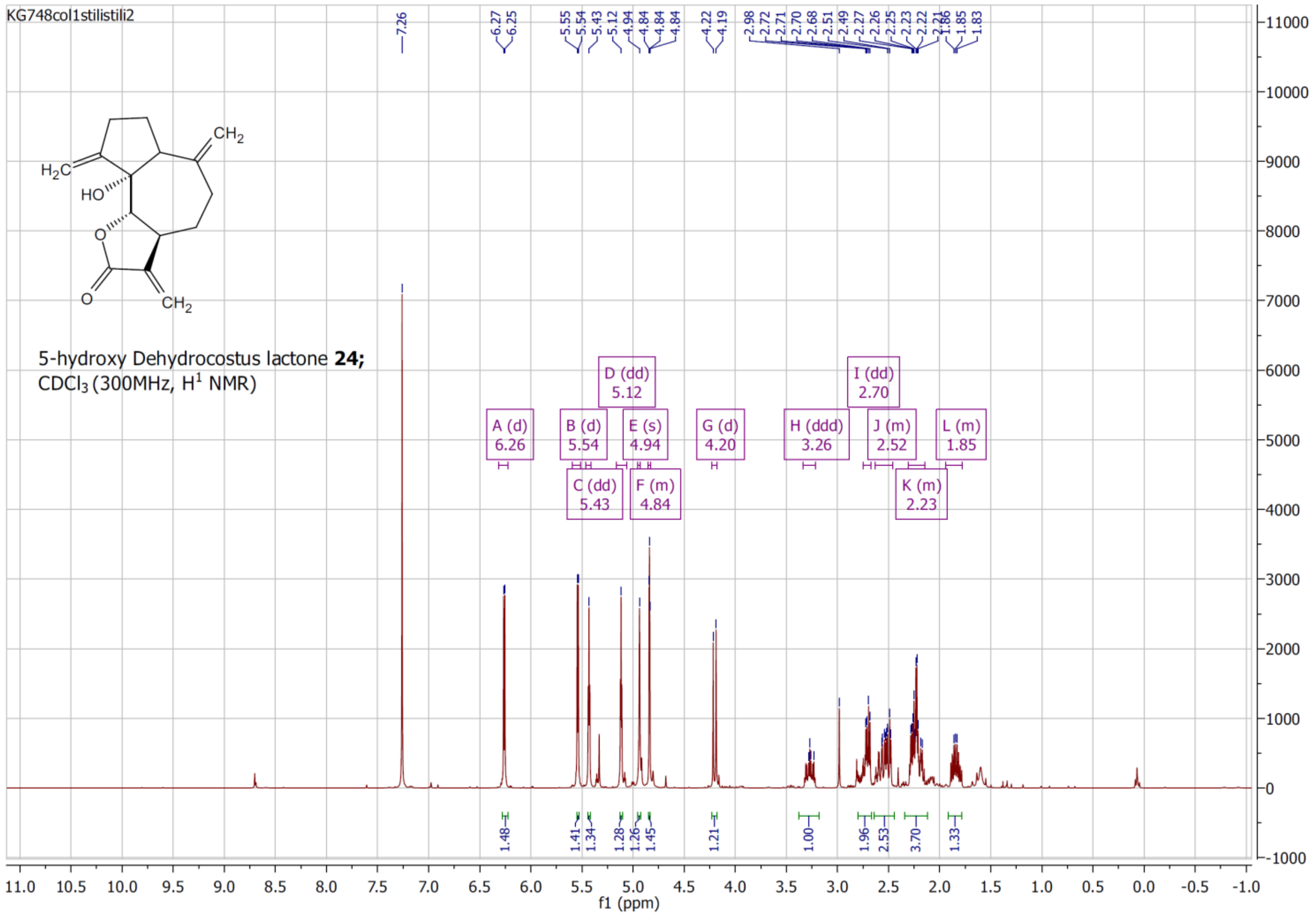
KG748col1stilistili2
7.26
6.27 6.25
5.55 5.54 5.43 5.12 4.94 4.84 4.84 4.84
4.22 4.19
2.98 2.72 2.71 2.70 2.68 2.51 2.49 2.27 2.26 2.25 2.23 2.22 2.21 1.86 1.85 1.83
CH_2
H_2C
HO
O
O
CH_2
5-hydroxy Dehydrocostus lactone **24;**
$CDCl_3$ (300MHz, H[1] NMR)
A (d) 6.26
B (d) 5.54
C (dd) 5.43
D (dd) 5.12
E (s) 4.94
F (m) 4.84
G (d) 4.20
H (ddd) 3.26
I (dd) 2.70
J (m) 2.52
K (m) 2.23
L (m) 1.85
1.48 1.41 1.34 1.28 1.26 1.45 1.21 1.00 1.96 2.53 3.70 1.33
11.0 10.5 10.0 9.5 9.0 8.5 8.0 7.5 7.0 6.5 6.0 5.5 5.0 4.5 4.0 3.5 3.0 2.5 2.0 1.5 1.0 0.5 0.0 -0.5 -1.0
f1 (ppm)
11000 10000 9000 8000 7000 6000 5000 4000 3000 2000 1000 0 -1000

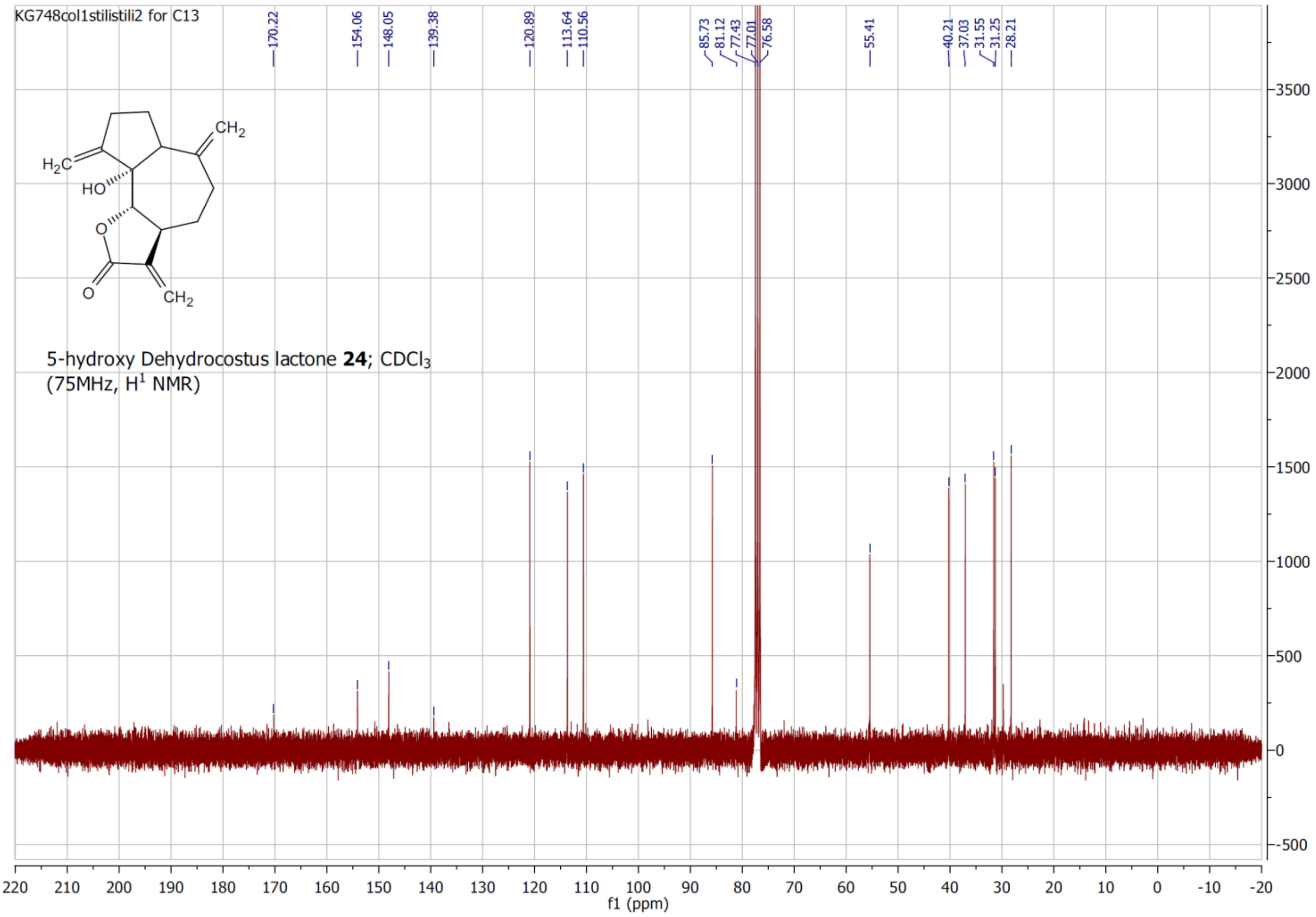
KG748col1stilistili2 for C13
170.22
154.06 148.05 139.38
120.89 113.64 110.56
85.73 81.12 77.43 77.01 76.58
55.41
40.21 37.03 31.55 31.25 28.21
CH_2
H_2C
HO
O
O
CH_2
5-hydroxy Dehydrocostus lactone **24**; $CDCl_3$
(75MHz, H[1] NMR)
220 210 200 190 180 170 160 150 140 130 120 110 100 90 80 70 60 50 40 30 20 10 0 -10 -20
f1 (ppm)
3500 3000 2500 2000 1500 1000 500 0 -500

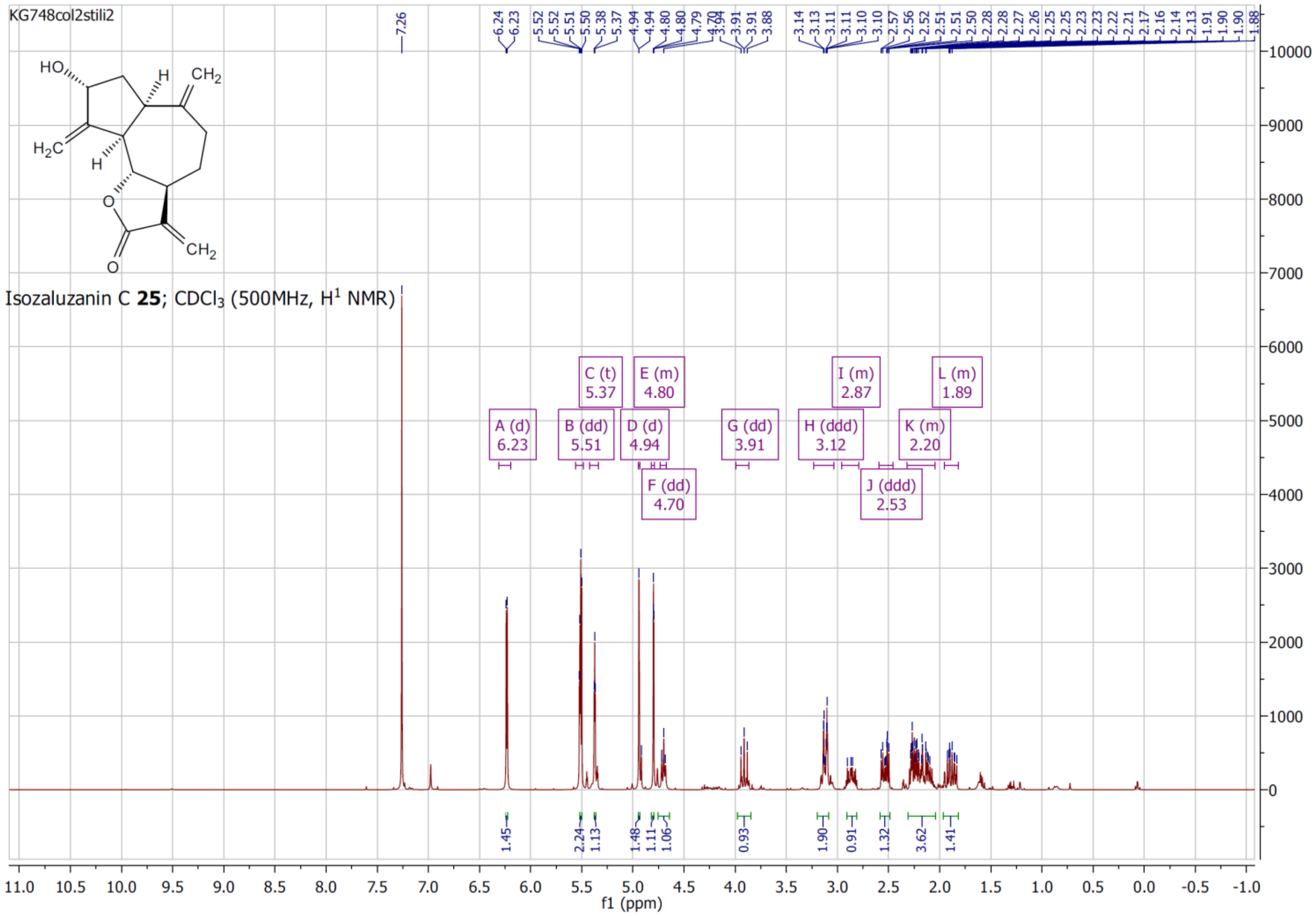
KG748col2stili2
Isozaluzanin C 25; CDCl3 (500MHz, H1 NMR)
f1 (ppm)

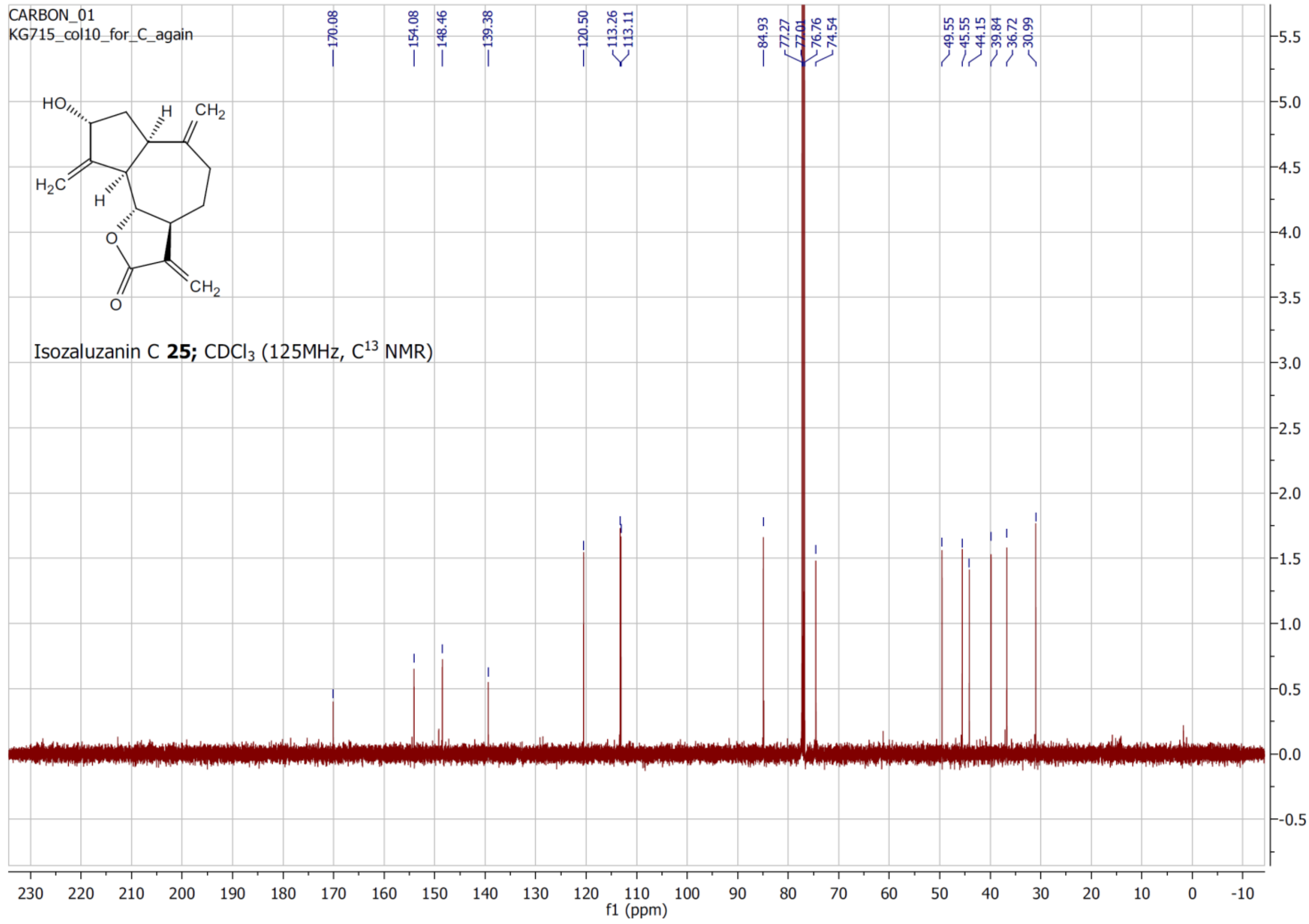
CARBON_01
KG715_col10_for_C_again
Isozaluzanin C 25; CDCl3 (125MHz, C13 NMR)
f1 (ppm)

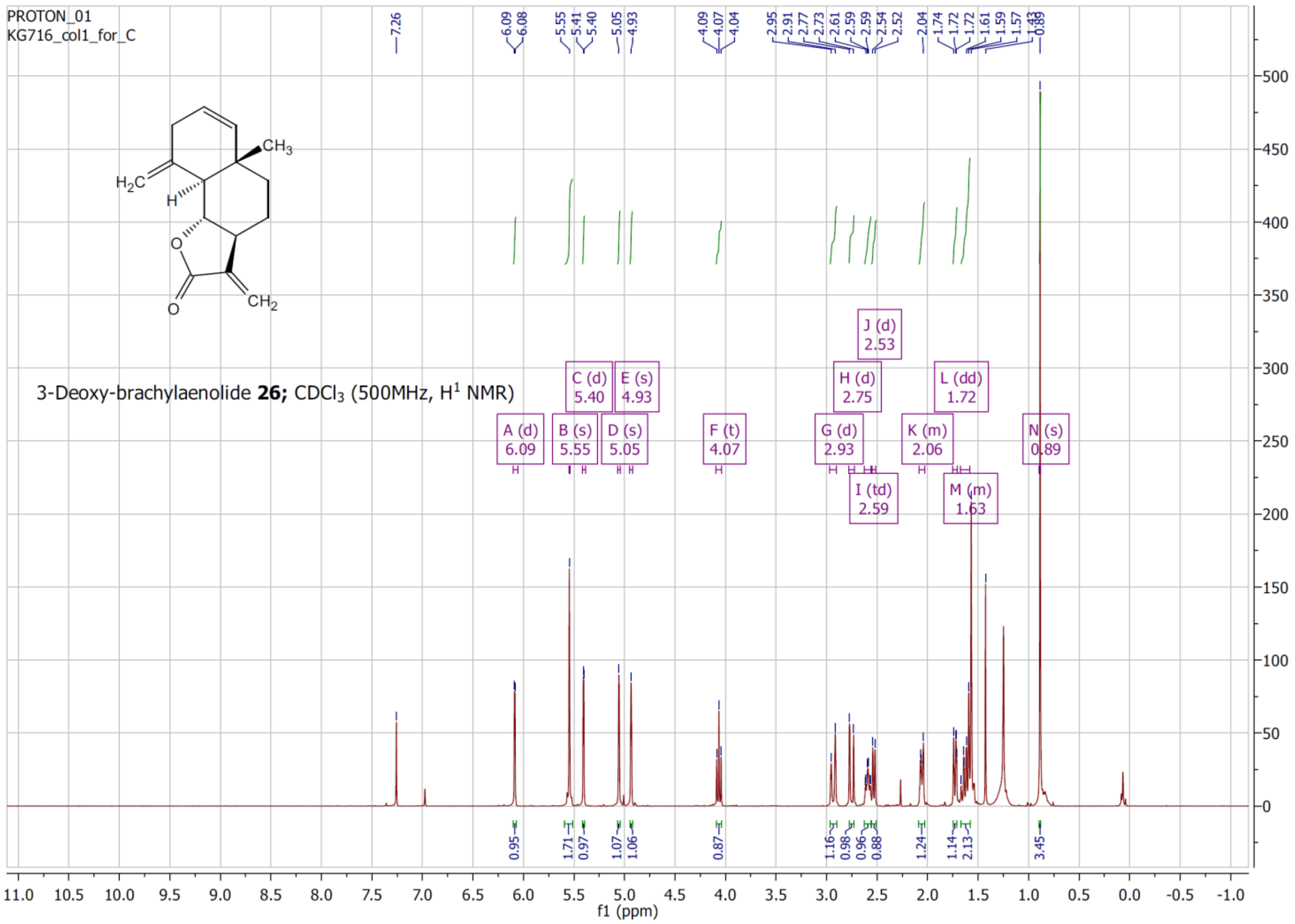
PROTON_01
KG716_col1_for_C
3-Deoxy-brachylaenolide 26; CDCl3 (500MHz, H1 NMR)
A (d) 6.09
B (s) 5.55
C (d) 5.40
D (s) 5.05
E (s) 4.93
F (t) 4.07
G (d) 2.93
H (d) 2.75
I (td) 2.59
J (d) 2.53
K (m) 2.06
L (dd) 1.72
M (m) 1.63
N (s) 0.89
f1 (ppm)

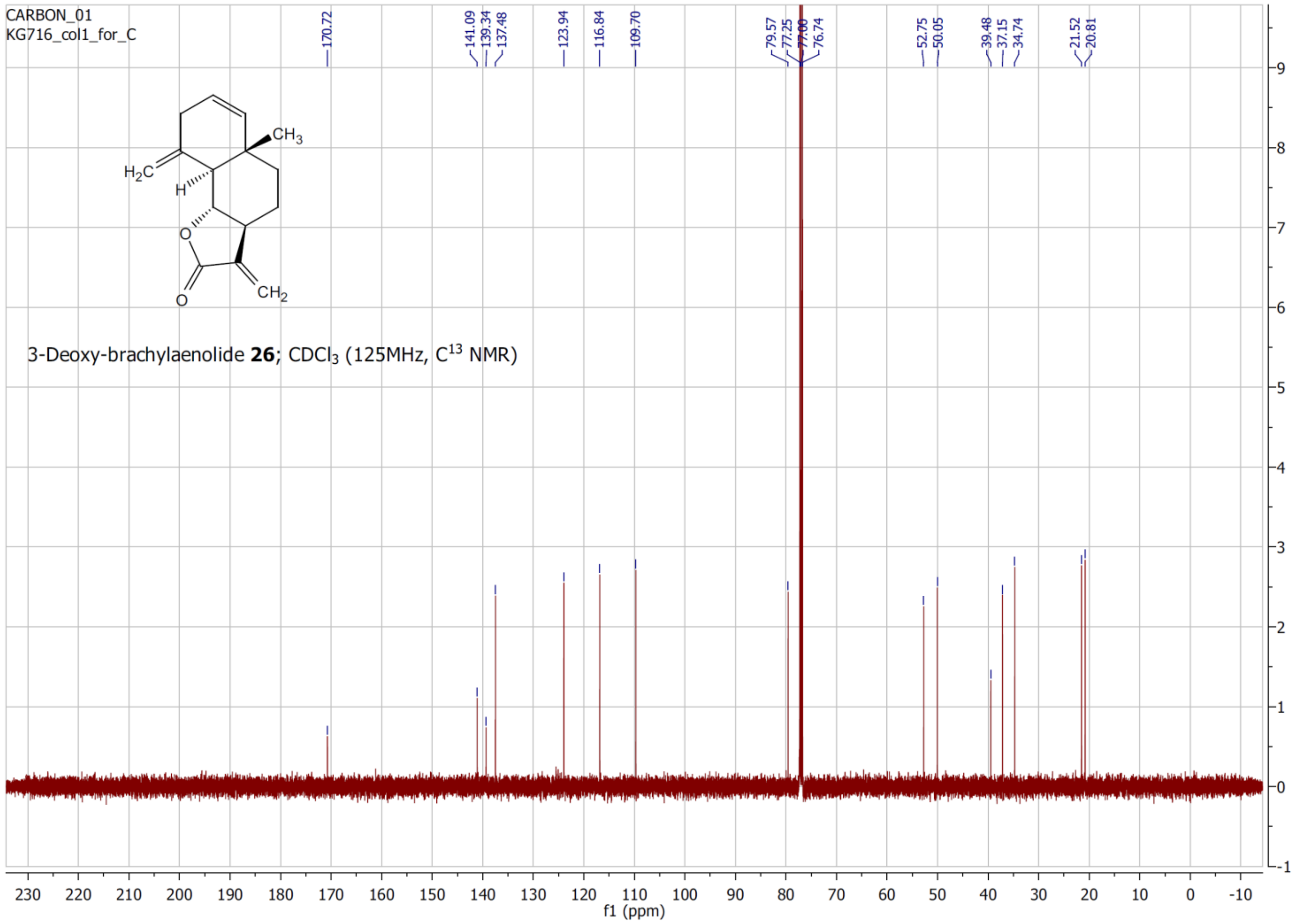
CARBON_01
KG716_col1_for_C
3-Deoxy-brachylaenolide 26; CDCl3 (125MHz, C13 NMR)
170.72
141.09
139.34
137.48
123.94
116.84
109.70
79.57
77.25
77.00
76.74
52.75
50.05
39.48
37.15
34.74
21.52
20.81
f1 (ppm)

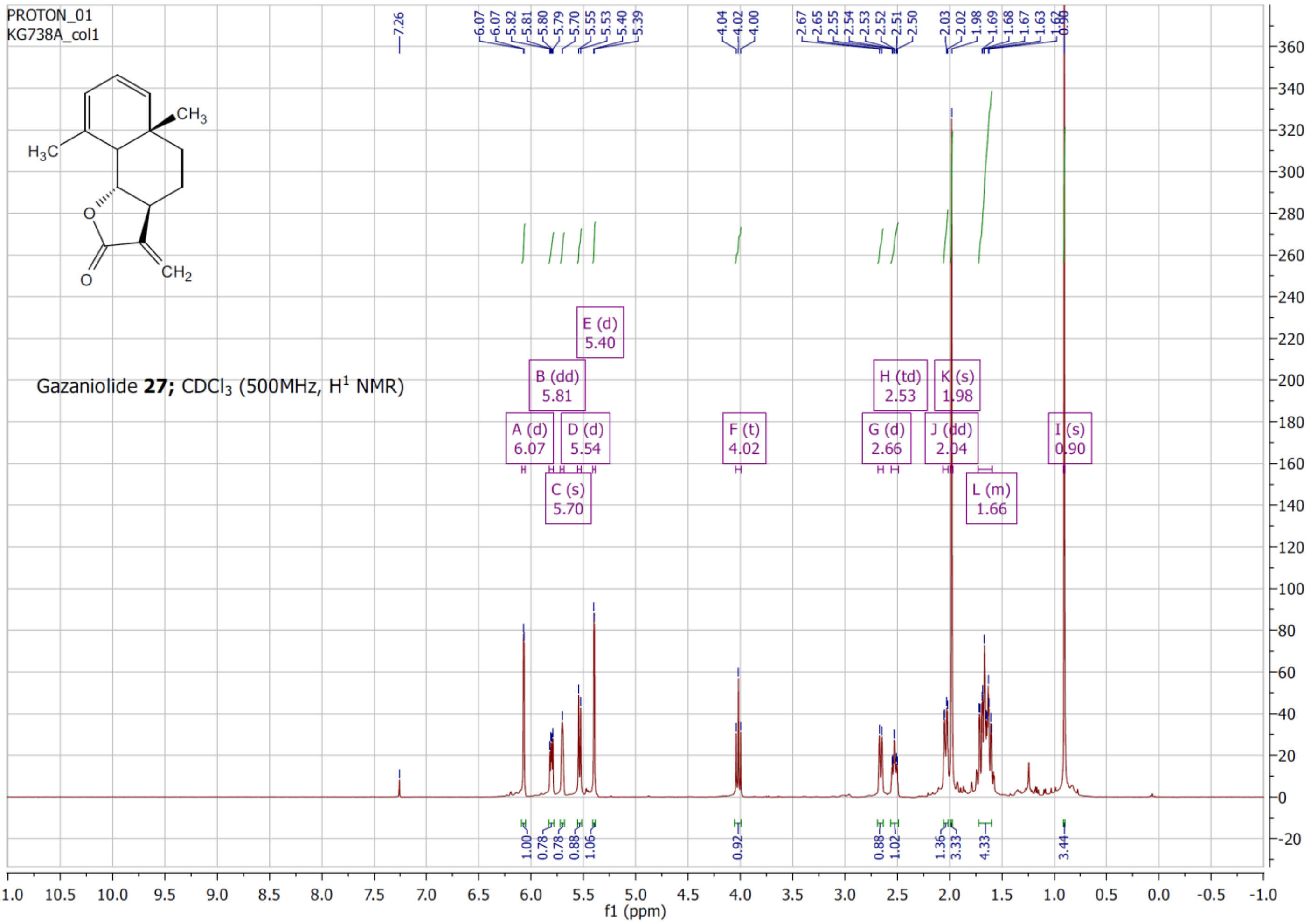
PROTON_01
KG738A_col1
Gazaniolide 27; CDCl3 (500MHz, H1 NMR)
A (d) 6.07
B (dd) 5.81
C (s) 5.70
D (d) 5.54
E (d) 5.40
F (t) 4.02
G (d) 2.66
H (td) 2.53
J (dd) 2.04
K (s) 1.98
L (m) 1.66
I (s) 0.90
f1 (ppm)

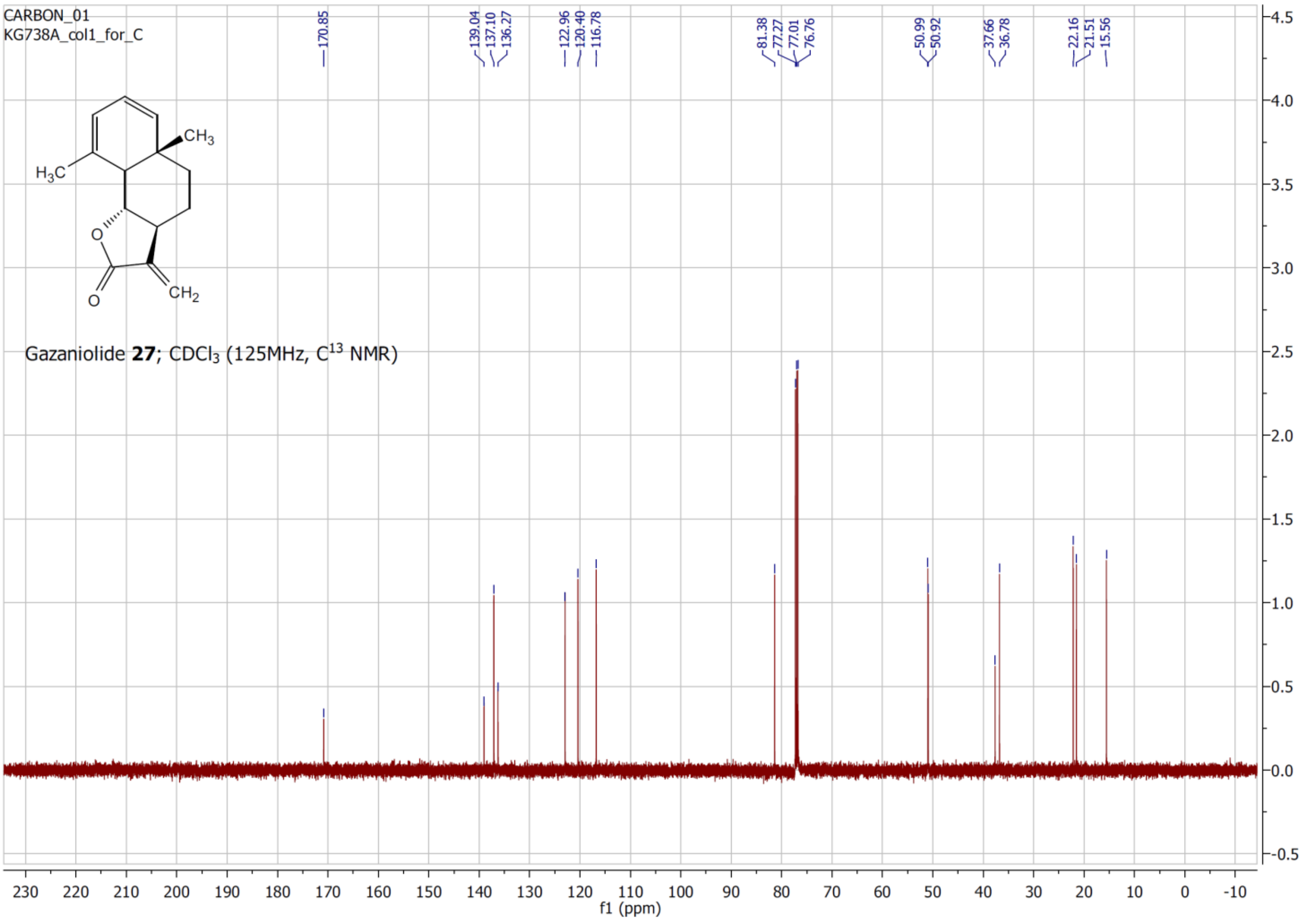
CARBON_01
KG738A_col1_for_C
Gazaniolide 27; CDCl3 (125MHz, C13 NMR)
170.85
139.04
137.10
136.27
122.96
120.40
116.78
81.38
77.27
77.01
76.76
50.99
50.92
37.66
36.78
22.16
21.51
15.56
f1 (ppm)

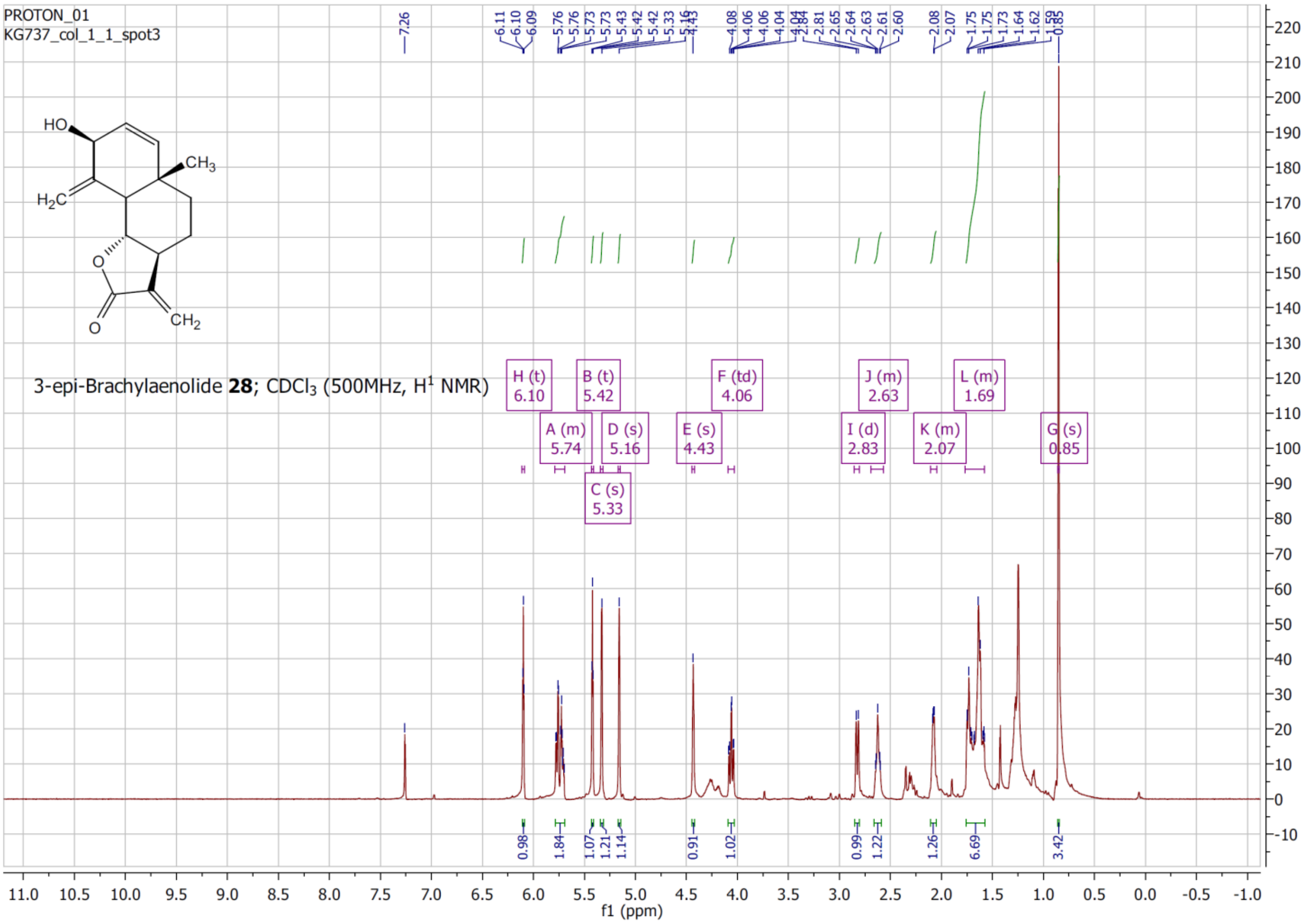
PROTON_01
KG737_col_1_1_spot3
HO
CH3
H2C
O
O
CH2
3-epi-Brachylaenolide 28; CDCl3 (500MHz, H1 NMR)
H (t) 6.10
B (t) 5.42
F (td) 4.06
J (m) 2.63
L (m) 1.69
A (m) 5.74
D (s) 5.16
E (s) 4.43
I (d) 2.83
K (m) 2.07
G (s) 0.85
C (s) 5.33
f1 (ppm)

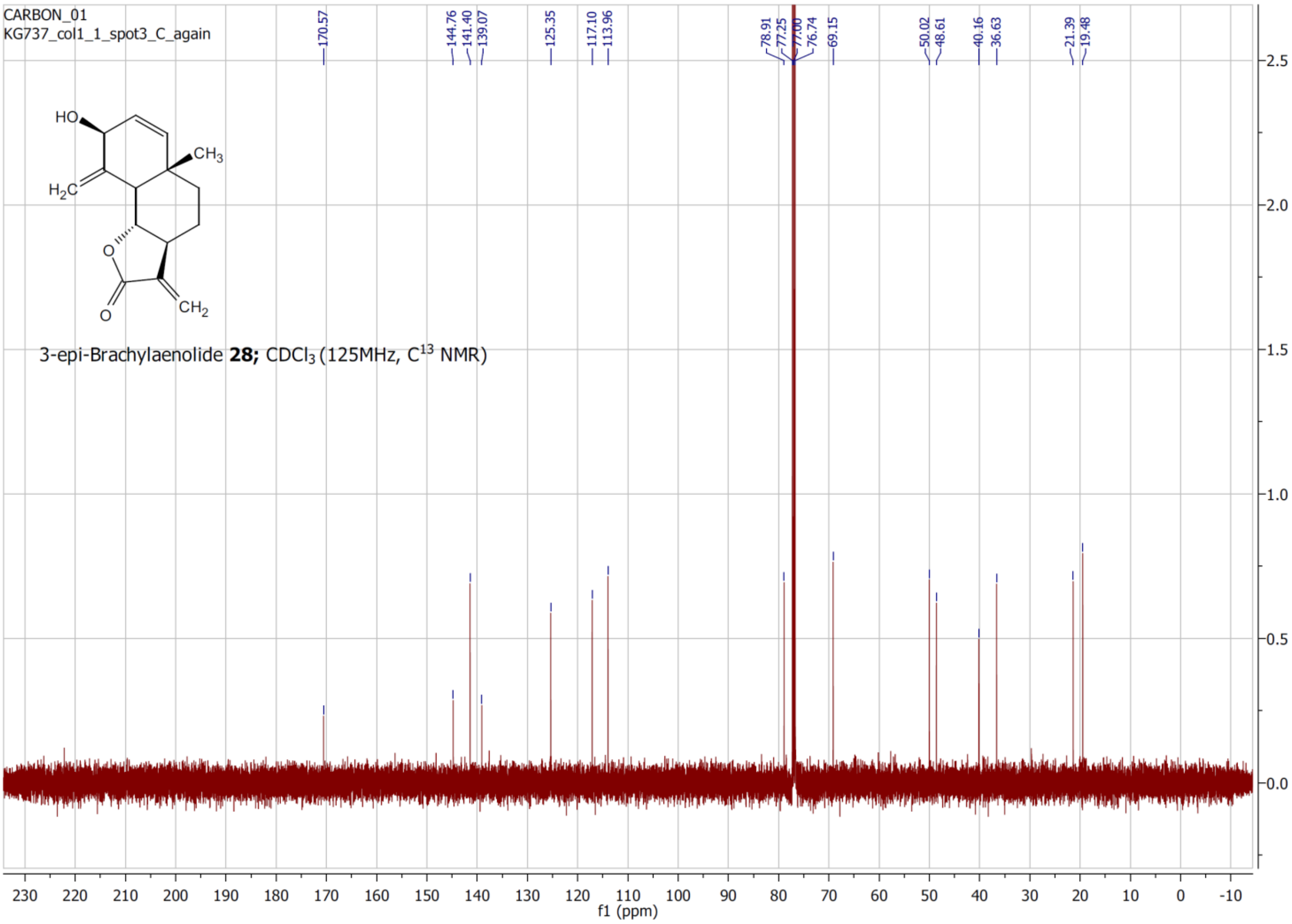
CARBON_01
KG737_col1_1_spot3_C_again
170.57
144.76
141.40
139.07
125.35
117.10
113.96
78.91
77.25
77.00
76.74
69.15
50.02
48.61
40.16
36.63
21.39
19.48
HO
CH3
H2C
O
O
CH2
3-epi-Brachylaenolide 28; CDCl3 (125MHz, C13 NMR)
f1 (ppm)

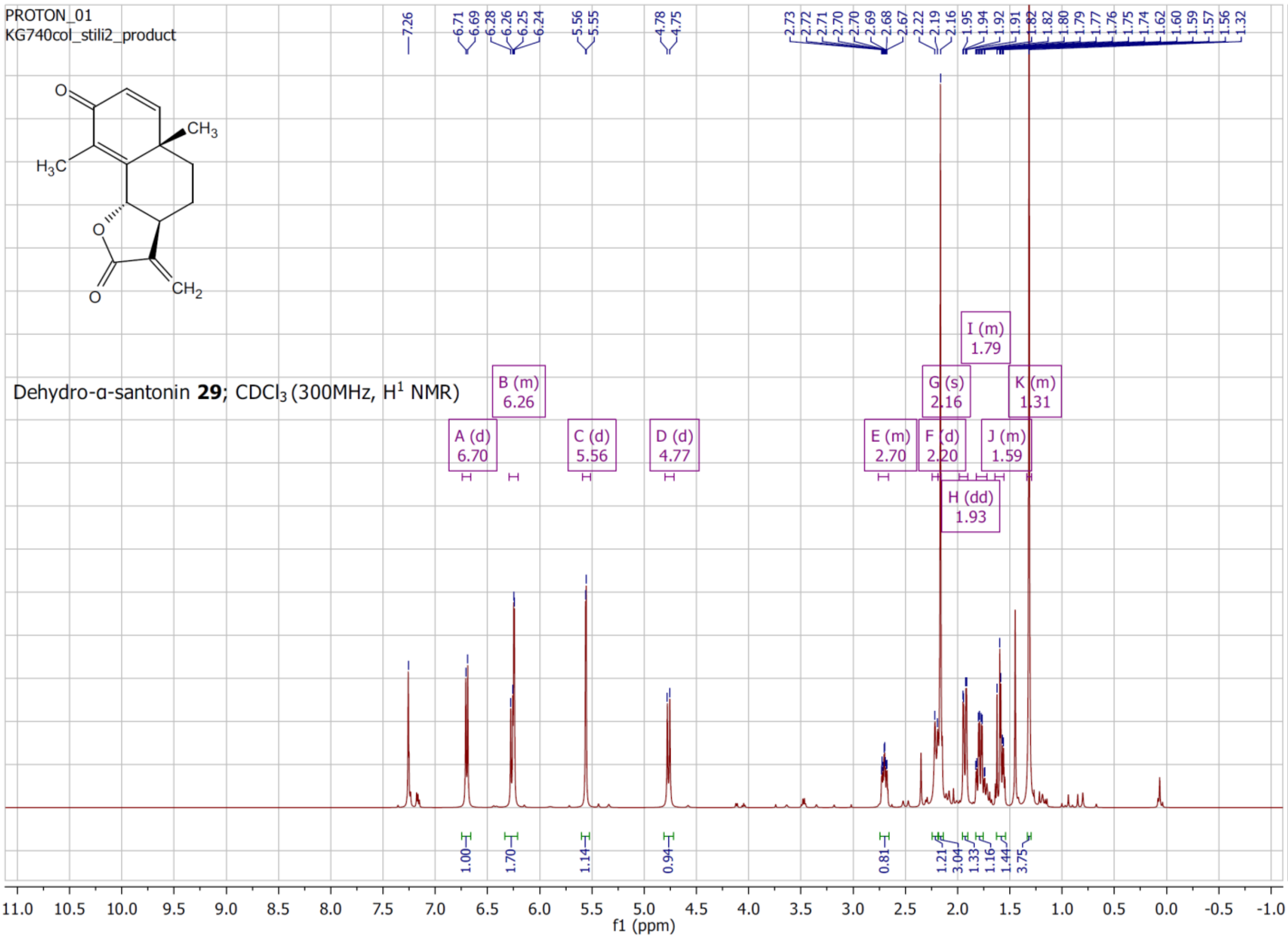
PROTON_01
KG740col_stili2_product
7.26
6.71
6.69
6.28
6.26
6.25
6.24
5.56
5.55
4.78
4.75
2.73
2.72
2.71
2.70
2.70
2.69
2.68
2.67
2.22
2.19
2.16
1.95
1.94
1.92
1.91
1.82
1.82
1.80
1.79
1.77
1.76
1.75
1.74
1.62
1.60
1.59
1.57
1.56
1.32
O
CH3
H3C
O
O
CH2
Dehydro-α-santonin 29; CDCl3 (300MHz, H1 NMR)
A (d) 6.70
B (m) 6.26
C (d) 5.56
D (d) 4.77
E (m) 2.70
F (d) 2.20
G (s) 2.16
H (dd) 1.93
I (m) 1.79
J (m) 1.59
K (m) 1.31
1.00
1.70
1.14
0.94
0.81
1.21
3.04
1.33
1.16
1.44
3.75
f1 (ppm)

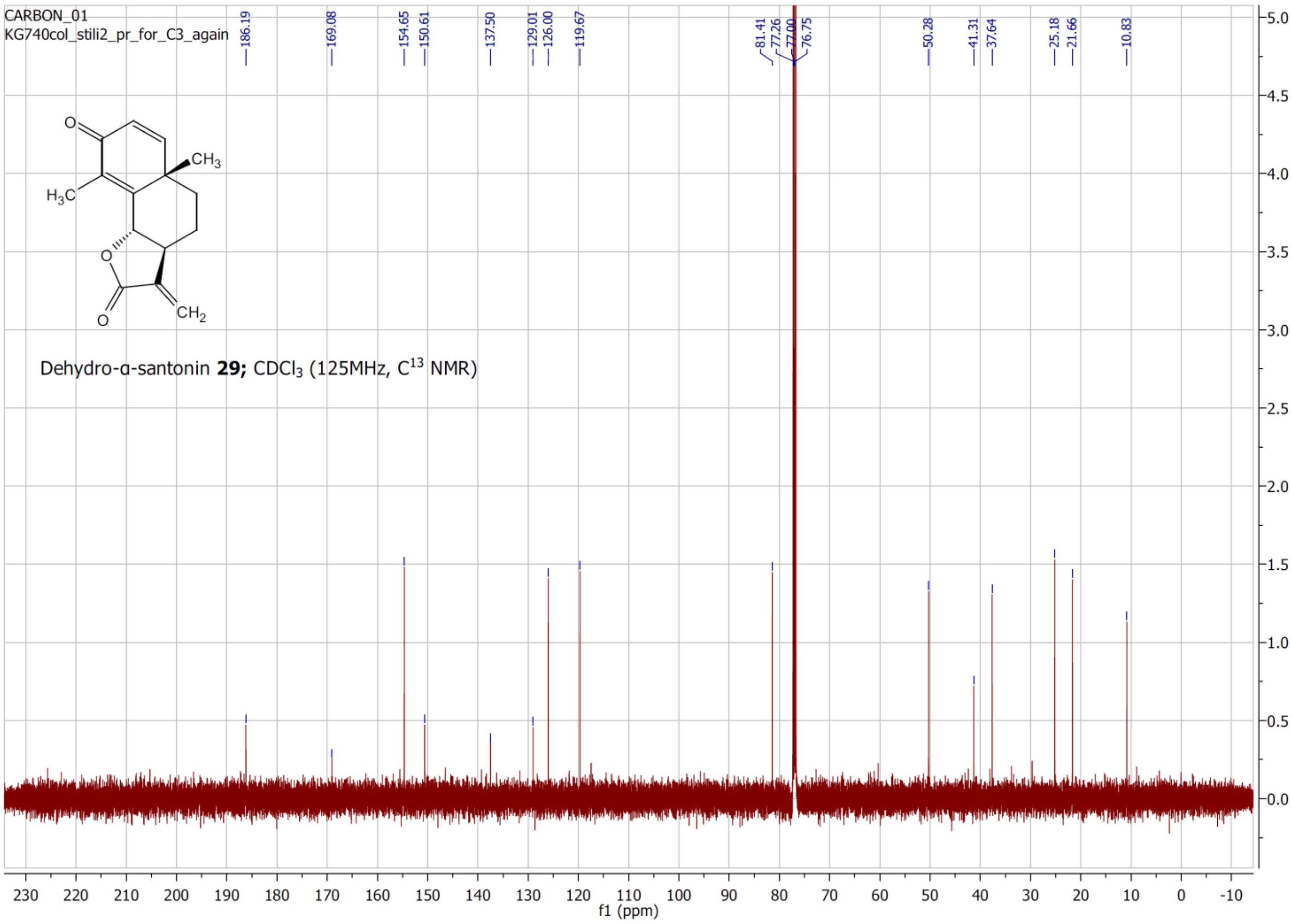
CARBON_01
KG740col_stili2_pr_for_C3_again
186.19
169.08
154.65
150.61
137.50
129.01
126.00
119.67
81.41
77.26
77.00
76.75
50.28
41.31
37.64
25.18
21.66
10.83
O
CH3
H3C
O
O
CH2
Dehydro-α-santonin 29; CDCl3 (125MHz, C13 NMR)
f1 (ppm)

8. References

[30] C. Vergne, J. Appenzeller, C. Ratinaud, M-T. Martin, C. Debitus, A. Zaparucha, A. Al-Mourabit, *Org. Letters* **2008**, *10*, 493-496.

[31] S. Chen, M. S. Hossain, F. Jr W. Foss, *ACS Sustainable Chem. Eng*. **2013**, *1*, 1048-1051.

[32] J. Y. Choi, E. H. Choi, H. W. Jung, J.S. Oh, W. H. Lee, J.G. Lee, J. K. Son, Y. Kim, S. H. Lee, *Arch Pharm Res* **2008,** *31*, 294-299.

[33] C.J. Bethencourt-Estrella, N. Nocchi, A. López-Arencibia, D. S. Nicolás-Hernández, M. L. Souto, B. Suárez-Gómez, A. R. Díaz-Marrero, J. J. Fernández, J. Lorenzo-Morales, J. E. Piñero, *Pharmaceuticals* **2021**, *14*, 1095.

[34] M. Yang, J. Zhang, Y. Li, X. Han, K. Gao, J. Fang, *Archives of Biochemistry and Biophysics* **2016**, *607*, 20-26.

[35] M. Vasquez, L. Quijano, F. R. Fronczek, F. A. Macias, L. E. Urbatsch, P. B. Cox, N. H. Fischer, *Phytochemistry* **1990**, *29*, 561-565.

[36] V. P. Sülsen, E. F. Lizarraga, O. G. Elso, N. Cerny, A. S. Alberti, A. E. Bivona, E. L. Malchiodi, S. I. Cazorla, C. A. N. Catalán, *Molecules* **2019**, *24*, 1209.

[37] F. A. Macías, R. F. Velasco, D. Castellano, J. C. G. Galindo, *J. Agric. Food Chem* **2005**, *53*, 3530–3539.

[38] F. F.P. Arantes, L. C.A. Barbosa, E. S. Alvarenga, A. J. Demuner, D. P. Bezerra, J. R.O. Ferreira, L. V. Costa-Lotufo, C. Pessoa, M. O. Moraes, *European Journal of Medicinal Chemistry* **2009**, *44*, 3739–3745.
